# Abiraterone Acetate Complexes with Biometals: Synthesis, Characterization in Solid and Solution, and the Nature of Chemical Bonding

**DOI:** 10.3390/pharmaceutics15092180

**Published:** 2023-08-23

**Authors:** Petr Buikin, Anna Vologzhanina, Roman Novikov, Pavel Dorovatovskii, Alexander Korlyukov

**Affiliations:** 1 A. N. Nesmeyanov Institute of Organoelement Compounds, RAS, 119334 Moscow, Russia; peterzzz@mail.ru; 2 N. S. Kurnakov Institute of General and Inorganic Chemistry, RAS, 119991 Moscow, Russia; 3 N. D. Zelinsky Institute of Organic Chemistry, RAS, 119991 Moscow, Russia; novikovfff@bk.ru; 4 Kurchatov Institute, National Research Center, 123182 Moscow, Russia; paulgemini@mail.ru

**Keywords:** active pharmaceutical ingredient, drug design, density functional calculations, QTAIM, NMR studies, metallopharmaceuticals, X-ray diffraction

## Abstract

Abiraterone acetate (AbirAc) is the most used steroidal therapeutic agent for treatment of prostate cancer. The mainly hydrophobic molecular surface of AbirAc results in its poor solubility and plays an important role for retention of abiraterone in the cavity of the receptor formed by peptide chains and heme fragments. In order to evaluate the hydrolytic stability of AbirAc, to modify its solubility by formation of new solid forms and to model bonding of this medication with the heme, a series of d-metal complexes with AbirAc was obtained. AbirAc remains stable in water, acetonitrile, tetrahydrofuran, and ethanol, and readily interacts with dications as a terminal ligand to create discrete complexes, including [FePC(AbirAc)_2_] and [ZnTPP(AbirAc)] (H_2_PC = phthalocyanine and H_2_TPP = 5,10,15,20-tetraphenylporphyrine) models for ligand–receptor bonding. In reactions with silver(I) nitrate, AbirAc acts as a bridge ligand. Energies of chemical bonding between AbirAc and these cations vary from 97 to 235 kJ mol^−1^ and exceed those between metal atoms and water molecules. This can be indicative of the ability of abiraterone to replace solvent molecules in the coordination sphere of biometals in living cells, although the model [ZnTPP] complex remains stable in CDCl_3_, CD_2_Cl_2_, and 1,1,2,2-tetrachloroethane-*d_2_* solvents and decomposes in polar dimethylsulfoxide-*d_6_* and methanol-*d_4_* solvents, as follows from the ^1^H DOSY spectra. Dynamics of its behavior in 1,1,2,2-tetrachloroethane-*d_2_* were studied by ROESY and NMR spectra.

## 1. Introduction

The number of medications based on active pharmaceutical ingredients (API) in the form of salts and complexes has increased in recent decades, both in the total number and in the number of cations used [1,2,3]. This growth is related to ability of cations to tune physical chemical properties of an API, such as solubility or tabletability [4,5,6,7,8], and increased selectivity of their supramolecular binding with biological macromolecules and/or bioactivity [9,10], as well as to the existence of metallopharmaceuticals [11,12,13,14], metallodrugs, and bioMOFs with controlled release of API [15,16,17,18,19,20]. The Orange Book of Approved Drug Products with Therapeutic Equivalence Evaluations [21] contains information about salts of sodium, potassium, calcium, magnesium, iron, platinum, silver, and zinc. Other medications contain bismuth [22], lithium [23], gold [24], and other metal ions. The majority of these salts contain biometals, either present in the human body in relatively large amounts (sodium, potassium, calcium), or known as essential trace metals found in metalloproteins and enzymes (magnesium, zinc, iron).

The complexes of an API with the latter elements as well as with the other biometals present in enzymes (copper, manganese, cobalt, etc.) can also be used as model systems for analysis of chemical bonding between the API and cofactors in living systems [25]. These complexes give valuable information about the number of API molecules able to interact with the cation, the stereochemistry of its coordination environment, and the functional groups involved in chemical bonding [26]. Using density functional (DFT) calculations, data about charge density redistribution and energies of API–metal bonding can be obtained as well [27,28]. Finally, such complexes can be used in the first stage of DFT simulation of metal–ligand interactions in the biological environment [29].

**Scheme 1:**
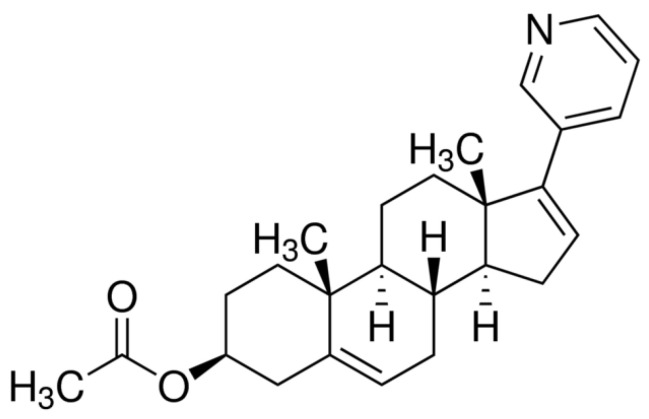
Molecular structure of AbirAc.

Abiraterone acetate (AbirAc, Scheme 1), commercially available as Zytiga and Yonsa, is the most used API for the treatment of prostate cancer. Abiraterone acetate is known to be hydrolyzed by pancreatic cholesterol esterase to afford pure abiraterone, AbirOH, which inhibits the biosynthesis of androgens [30]. Abiraterone in its stable crystalline form is not druggable due to its extremely low solubility in aqueous media (<0.5 μg/mL) [31]. Abiraterone acetate is even less soluble in water so Zytiga contains it in micronized form [32]. Moreover, it represents the largest food effect of all marketed drugs dependent on the fat content of the food [33]. Thus, attempts to find more soluble and less food-dependent forms of abiraterone are still ongoing. The search for novel solid forms of AbirAc with enhanced solubility included co-crystallization of AbirAc with some co-formers [34,35] and formation of (HAbirOH)Hal · H_2_O salts (Hal = Cl^–^ [36]; Br^–^ [37]). Based on numerous X-ray diffraction data of ligand (AbirOH): receptor (human cytochrome P450 17A1 or CYP17A1) complexes, the most specific feature of their binding is coordination between the iron atom of the heme fragment and the nitrogen atom N_Py_ of the pyridine-3-yl ring of abiraterone [38,39,40,41,42]. The steroid moiety of abiraterone forms numerous hydrophobic interactions, while the hydroxyl group can take part in H-bonding. The AbirAc is a neutral ditopic molecule, potentially able to act as a terminal ligand or a linker to bioMOFs. Hence, we attested its potential to obtain metallopharmaceuticals based on such biometals as Fe(II), Co(II), Cu(II), Ni(II), and Zn(II), as well as two 5d transition metals, Ag(I) and Cd(II).

## 2. Materials and Methods

### 2.1. General Procedures

All reagents and solvents were purchased from Sigma Aldrich (Merck LLC, Moscow, Russia) and used as supplied (analytical grade of purity). See Supporting Information for synthetic details and spectra. ^1^H and ^13^C NMR spectra were recorded on a 300 MHz (300.1, 75.5 MHz, respectively) and 400 MHz (400.1, 100.6 MHz, respectively) Bruker Avance spectrometers (Bruker BioSpin GMBH, Rheinstetten, Germany) in CDCl_3_, CD_2_Cl_2_, TCE-*d*_2_ (1,1,2,2-tetrachloroethane), DMSO-*d*_6_, and methanol-*d*_4_ solutions using 0.05% Me_4_Si as the external or internal standard. Determinations of structures and stereochemistry of obtained compounds and assignments of ^1^H, ^13^C signals were conducted with the aid of 2D COSY, TOCSY, NOESY, ROESY, edited-HSQC, and HMBC spectra, as well as using diffusion-ordered NMR studies (DOSY-LED). The powder X-ray diffraction patterns of all the samples were obtained in reflection mode. The measurements were performed with a Bruker D8 Advance diffractometer (Bragg-Brentano geometry, Bruker AXS, Inc., Madison, WI, USA) equipped with motorized slits and a LynxEye 1D position-sensitive detector (CuKa, Ni-filter). The measurement range was 2Ω = 3–60°. Rietveld full-profile X-ray analysis of the patterns of crystalline substances was conducted using TOPAS 4.2 software [43] and is provided on Figures S1–S9 (Supporting Information). The background, profile, preferred orientation, scale factor(s), and unit cell parameters were refined. The preferred orientation was taken into account with the spherical harmonics approach [44].

### 2.2. Crystallographic Details

The intensities of reflections for **1**, **2**, **4**–**6**, and **11**–**14** were measured with a Bruker APEX DUO diffractometer using MoKα (λ = 0.71073 Å) radiation (Bruker AXS, Inc., Madison, WI, USA). X-ray diffraction data for **3**, **7**–**10** were collected by the “Belok/XSA” beamline of the Kurchatov Synchrotron Radiation Source [45,46]. Diffraction patterns were collected using a 1-axis MarDTB goniometer equipped with a Rayonix SX165 CCD 2D positional sensitive CCD detector (λ = 0.745 Å, φ-scanning in 1.0° steps) in direct geometry with the detector plane perpendicular to the beam. For each data set, ~120 diffraction frames were collected at 100 K. The data were indexed and integrated by XDS (ver. 2023) software suite [47]. Crystal structures were solved by the dual-space algorithm [48] and refined by the full-matrix least squares against F^2^. Non-hydrogen atoms were refined in anisotropic approximation except disordered solvent molecules in **4**. Hydrogen atoms were included in the refinement by the riding model with U_iso_(H) = 1.5 U_eq_(X) for methyl and hydroxo groups, and with 1.2 U_eq_(X) for the other atoms. Contribution of highly disordered solvent molecules to the structure factors of **14** was taken into account using solvent mask procedure of OLEX2 [49]. Complex **6** is a twin which was integrated using CELL_NOW and TWINABS algorithms, and refined using the BASF/HKLF 5 combination of instructions. All calculations were conducted using the SHELXTL ver. 2018/3 [50] and OLEX2 [49] program packages.

Experimental details and the results of these refinements are listed in Tables S1 and S2 (Supporting Information). Crystallographic information files are available from the Cambridge Crystallographic Data Center upon request (https://ccdc.cam.ac.uk/structure, (accessed on 22 August 2023) deposition numbers are 2215031–2215044).

### 2.3. DFT Calculations

Preliminary optimization of molecular geometry of complexes **1**, **2**, **5**, **7**–**10**, **12**, and **13** was carried out using the Q-Chem program (PBE/6-31G(d)) [51]. Thereafter, the final optimization, frequencies calculations, and electron density calculation were performed with the Orca 5.1 program [52] and PBE0/def2-TZVP method/basis set combination. The calculated electron density function for the complexes studied was analyzed with the QTAIM approach using Multiwfn software, ver. 3.8 [53]. Coordinates of atoms for optimized complexes are given as Supporting Information.

## 3. Results

### 3.1. Synthesis

Our studies were initiated by investigating the reaction between abiraterone acetate and transition metal salts (Table 1). Previously, AbirAc was found to be soluble in ethanol (EtOH; at r.t. mole fraction solubility S × 10^5^ = 1106.45(2)), tetrahydrofuran (THF; S × 10^5^ > 10,000), acetonitrile (CH_3_CN; S × 10^5^ = 209.76(7)), and acetone (S × 10^5^ = 1806.39(6)) [31]. We attested these solvents as well as diethyl ether (Et_2_O) and dichloromethane (CH_2_Cl_2_) for complexation reactions. The majority of salts are insoluble in Et_2_O, CH_2_Cl_2_, and acetone (denoted with a down arrow in Table 1). Despite CoCl_2_·2H_2_O, Fe(acac)_3_, and Fe(AcO)_2_ (acac^–^ = acetylacetonate; AcO^–^ = acetate) formation with AbirAc transparent solutions, no complexes precipitated from these mixtures (denoted with a dash). During interaction of CuCl_2_·2H_2_O and AbirAc, only powders precipitated which could not be characterized using single crystal XRD. A total of 14 new complexes were obtained and characterized by ^1^H NMR and powder and single-crystal X-ray structural analysis. A total of 13 of these complexes contained abiraterone acetate coordinated by a metal atom. No hydrolytic elimination was observed in reaction conditions applied despite the slightly acidic nature of some solutions.

### 3.2. Molecular and Crystal Structures

**Figure 1:**
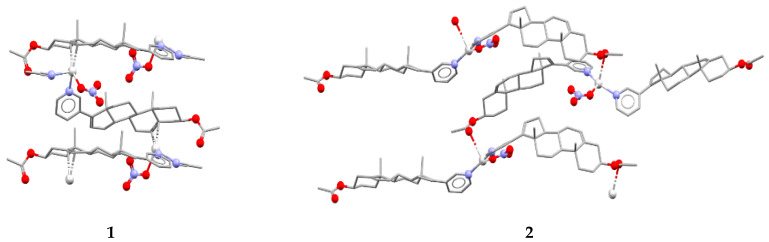
Fragment of 1D coordination polymers in **1** and **2**.

In reactions with silver(I) atoms in acetonitrile and ethanol, AbirAc acts as a linker to form 1D coordination polymers [Ag(AbirAc)(CH_3_CN)]NO_3_ (**1**) and [Ag(AbirAc)_2_]NO_3_ (**2**), respectively (Figure 1). In both cases, a Ag–N_Py_ bond as long as 2.157(6)–2.265(3) Å occurs. In addition, in (**1**), a double bond of steroid fragment of AbirAc is coordinated by a silver(I) atom through a π-bond (r(Ag–C) = 2.363(4)–2.475(4) Å); and in (**2**), one of two symmetrically independent AbirAc molecules forms an Ag–O bond with a carbonyl fragment (r(Ag–O) = 2.857(7) Å), while another AbirAc is terminal. In (**1**), a silver(I) atom beside an AbirAc coordinates a terminal nitrate-anion and an acetonitrile molecule to form a pseudo-trigonal pyramidal AgN_2_OC_2_ coordination polyhedron with a metal atom 0.19(1) Å shifted from the plane formed by two carbon atoms, an oxygen atom, and a nitrogen atom to the nitrogen atom of the acetonitrile molecule. In (**2**), a silver(I) atom is 0.10(1) Å shifted from the base of an AgN_2_O_2_ trigonal pyramid to an apical carbonyl oxygen atom.

**Figure 2:**
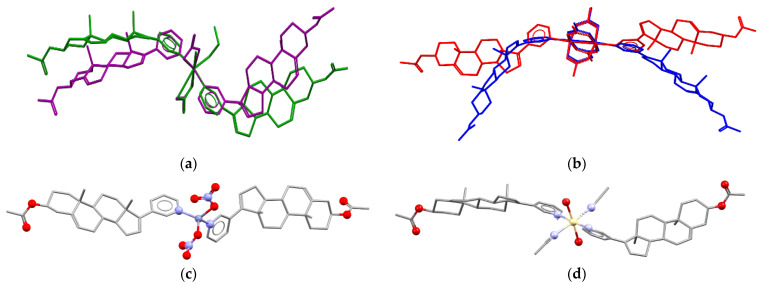
(**a**) Conformations of complexes [Cu(AbirAc)_2_(NO_3_)_2_] (violet) in solid **3** and [Cu(AbirAc)_2_(NO_3_)(H_2_O)(EtOH)]^+^ (green) in solid **4** with overlaid CuN_2_(NO_3_) fragments. (**b**) Conformations of [Cu_2_(AbirAc)_2_(AcO)_4_] complexes in **5** and **6** with overlaid [Cu_2_(AcO)_4_] units. Molecular structures of (**c**) **9** and (**d**) **10**. H atoms are omitted.

The molar ratio Ag(I):AbirAc in the reaction mixtures was the same; however, it varies in complexes (**1**) and (**2**). In copper(II)-containing mixtures, the molar ratio of reagents affected the Cu(II):AbirAc ratio in resulting complexes. For Cu(NO_3_)_2_:AbirAc = 1:1 solutions, isostructural complexes (**3**) and (**4**) were obtained where Cu(II):AbirAc ratio is equal to 1:2. In solid (**3**), [Cu(AbirAc)_2_(NO_3_)_2_] and [Cu(AbirAc)_2_(NO_3_)(H_2_O)(CH_3_CN)]^+^ complexes co-crystallize in 1:1 ratio, while in (**4**), [Cu(AbirAc)_2_(NO_3_)(H_2_O)(EtOH)]^+^ and [Cu(AbirAc)_2_(NO_3_)(H_2_O)_2_]^+^ complexes were found to have a ratio of 0.33:0.67 (Figure 2a). When the Cu(OAc)_2_:AbirAc ratio was taken as 2:1 in an attempt to obtain an AbirAc bridge between copper(II) atoms, complexes [Cu_2_(AbirAc)_2_(AcO)_4_] (**5**) and [Cu_2_(AbirAc)_2_(AcO)_4_]·2THF (**6**) based on binuclear “lantern” [Cu_2_(AcO)_4_] units precipitated as green plates (Figure 2b). For all these complexes, Cu(II)–N_Py_ bond lengths were equal to 1.979(5)–2.198(6) Å.

The reaction of Co(II), Ni(II), Zn(II) and Cd(II) nitrates with AbirAc in a 1:1 ratio in acetonitrile affords isostructural [M(AbirAc)_2_(CH_3_CN)_2_(H_2_O)_2_](NO_3_)_2_ (M = Co(II), **7**; M = Ni(II), **8** and M = Cd(II), **10**) and [Zn(AbirAc)_2_(NO_3_)_2_] (**9**) with terminal AbirAc, anions and solvent molecules, and metal atoms in, respectively, octahedral and tetrahedral environments (Figure 2c,d). Unfortunately, the only iron(II) complex with AbirAc was obtained by reaction of FeBr_3_ with AbirAc in acidic media and had the composition (AbirAcH)[FeBr_4_] (**11**). With the absence of HBr, single crystals of (HAbirOH)Br · H_2_O and colloid Fe(OH)_3_ precipitated from the same FeBr_3_:AbirAc = 1:1 mixture, and the structure of this salt has been published elsewhere [37]. The recrystallization of AbirAc from 2M and concentrated 48% HBr affords only the starting AbirAc. Thus, the presence of iron(III) salt abiraterone acetate can be hydrolyzed to AbirOH or HAbirOH^+^.

**Figure 3:**
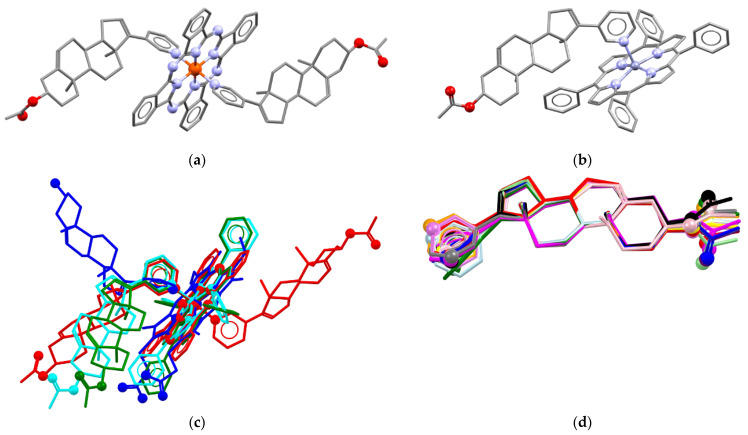
Molecular structures of (**a**) **12** and (**b**) **13**. (**c**) Comparison of molecular conformations in [FePC(AbirAc)_2_] (red), [ZnTPP(AbirAc)_2_] (**13**, cyan**; 14**, green) with [FePP(AbirOH)] (H_2_PP = protoporphyrine) in PDB 3ruk [38] (blue) with overlaid N_4_ equatorial planes. (**d**) Molecular conformations of AbirAc in **1** (red), **2** (orange), **3** (yellow), **4** (light-green), **5** (green), **6** (light-blue), **7** (cyan), **8** (blue), **9** (violet), **10** (purple), **11** (magenta), **12** (pink), **13** (grey), and **14** (black). Overlaid carbon atoms belong to olefin and methyl groups. Non-carbon atoms are depicted as spheres. H atoms are omitted.

In order to obtain an iron(II) complex with AbirAc, iron(II) phthalocyaninate (FePC) was also attested as complexing agent. The 2:1 mixture in acetonitrile afforded violet needle single crystals which were characterized by means of X-ray diffraction. The complex has the composition [FePC(AbirAc)_2_] (**12**), where two AbirAc molecules interact with the iron(II) atom through the nitrogen atoms of their heterocycles, and the metal atom realizes the octahedral coordination (Figure 3a). The AbirAc ligand with methyl groups directed from the PC ring is situated ca. 57° to the [FePC] plane, and the angle between the AbirAc involved in C-H…PC intramolecular bonding and the [FePC] plane is equal to 40°. Thus, not only Fe-N bond lengths, but also the mutual orientation of the iron(II) phthalocyaninate plane and steroid fragment in the complex closely resemble those observed in AbirOH:receptor complexes where the corresponding angle is close to 60° (Figure 3c). Finally, zinc(II) tetraphenylporphyrinate was allowed to react with AbirAc. Both reagents are soluble in alcohols and CH_2_Cl_2_ [31,54,55]. Dark-violet needles of the [ZnTPP(AbirAc)] (**13**) and [ZnTPP(AbirAc)]·CH_3_OH (**14**) compositions precipitated from ethanol and methanol, respectively. Zinc(II) in **13** and **14** is 0.306(1) and 0.228(4) Å shifted from the base of a square pyramid formed by TPP nitrogen atoms with a pyridyl nitrogen atom in the vertex (Figure 3b). Methyl groups and ring C of the steroid core of AbirAc take part in H…H bonding with a phenyl ring of TPP to form the angle between the AbirAc and the TPP plane close to that in **12**. The presence of intramolecular bonding between the AbirAc molecule and the TPP fragment in the solution was additionally confirmed by ^1^H NMR spectroscopy (see below).

Thus, abiraterone acetate can form complexes with transition metals through the nitrogen atom of the pyridine ring. Silver(I) atoms are able also to coordinate through acetate or olefin groups. Conformation of the AbirAc group in the studied solids is very rigid with only the pyridine ring and acetate fragments freely rotating along single bonds (Figure 3d).

Despite the presence of bulky AbirAc, coordinated water molecules as well as solvate methanol and pyridinium proton are able to take part in H-bonding. Parameters of H-bonds are listed in Table 2. In **3**, **4**, **7**, **8**, and **10**, the H-bonds occur between water molecules and uncoordinated nitrate anions. Solid **11** contains N1—H1···O2 bonds between pyridinium and acetate groups. In crystals of **14**, H-bonding between methanol and acetate groups can be seen. However, in all complexes but **11**, analysis of H-bonding and H-bonded architectures is prohibited by strong disorder of the nitrate anions, solvent, and AbirAc molecules.

### 3.3. Bond Energies

In general, EtOH, THF, and CH_3_CN solvent molecules used in the synthesis are able to act as ligands towards metal ions, and in solutions an excess of these molecules towards reagents is always present. Thus, the fact that AbirAc was coordinated by cations upon crystallization from these solvents can be indicative that the energy of the M–N_Py_ bond is similar or lower than that of M–N_Solv_ and M–O_Solv_ bonds. In order to check this assumption PBE0/Def2-TZVP calculations of isolated complexes **1**–**2**, **5**, **7**–**10**, **12**, and **13** were performed. Analysis of charge distribution within the Bader ‘Atoms in Molecules’ approach [56] revealed all expected bond critical points (bcp), including Ag-C in **1** and Ag-O=C in **2**. Bond energies estimated using the empirical correlation proposed by Espinosa, Mollins, and Lecomte (EML) [57], although having significant limitations [58], can nevertheless be used for semi-qualitative evaluation of the strength of intermolecular interactions (Table 3). Particularly, E_int_ estimated for Fe–PC and Zn–TPP binding using this correlation (−865.1 and −561.6 kJ mol^−1^) are close to calculated values (−946.5 and −629.1 kJ mol^−1^) given in Ref. [59]. The value of E(M-N_Py_) of −233.7…−235.5 and −160.1…−162.8 kJ mol^−1^ in **7** and **12** can also be compared with that of −248.3 estimated by Rodgers, Stanley, and Amunugama [60] and that of −139.9 kJ mol^−1^ calculated for [FePP(Py)_2_] by Liao and Scheiner [61].

Despite having a different charge, E_int_ for the bond between silver(I) and AbirAc is close to that for copper(II), cadmium(II), and zinc(II) atoms. E_int_(M–N_Py_) for the other transition metals decreases in the range of Ni(II) (c.a. –150 kJ mol^−1^)-Fe(II) (c.a. –160 kJ mol^−1^)-Co(II) (c.a. –235 kJ mol^−1^). For heteroligand complexes **7**, **8**, and **10**, E_int_ values fall from M–O_aqua_ to M–N_CH3CN_ and to M–N_Py_. The latter values are also lower than E_int_ for M–O_NO3_ bonds; thus, it is not surprising that we obtained the target complexes from metal nitrates, and that the majority of complexes realized the 2:1 ratio of AbirAc and cation.

**Figure 4:**
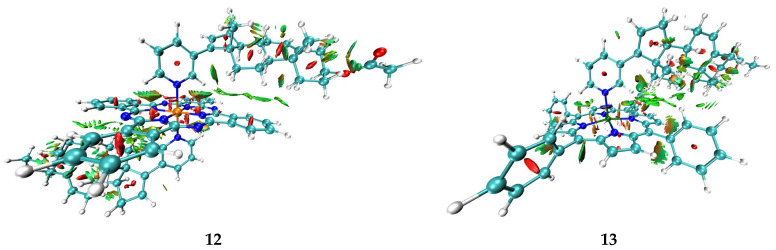
Isosurfaces of the RDG function (s(**r**) = 0.3) of **12** and **13**, colored according to λ_2_ values (−0.02–0.04 a.u.).

Taking into account data about the connectivity of biometals in proteins and enzymes, as well as the abovementioned regularities in E_int_ for metal–solvent and metal–abiraterone bonds, one can assume that AbirOH in the absence of steric hindrances can potentially replace water molecules in the coordination sphere of not only iron porphyrin but also of other biometals. At the same time, as the energy of interactions with heme iron is higher than that with other transition metals except for cobalt(II), it remains the main target of AbirOH in living cells. In addition, the abiraterone complexes with heme porphyrin as compared with ions connected with water and amino acids only are probably additionally stabilized with intramolecular bonding. Particularly, for complexes **12**–**14** few other bcp corresponding to intramolecular AbirAc…PC and AbirAc…TPP interactions were found. In Figure 4, intramolecular bonding in this complex is visualized by means of the NCI (non-covalent interaction) method [62]. This method relies on two functions: dimensionless reduced density gradient (RDG, s(**r**)) and signλ_2_ρ(**r**), which is a product of λ_2_ eigenvector by electron density at a given point. NCI analysis has shown that phthalocyanine and porphyrin moieties are bonded with abiraterone by a number of C-H…π interactions and C-H…N weak bonds (Figure 4). The isosurfaces of the RDG function that correspond to the former type of interactions can be described as shapeless fuzzy regions where the values of the signλ_2_ρ(**r**) function are close to zero. Such character of bonding corresponds to van der Waals interactions. In the case of C-H…N weak bonds, the isosurfaces of RDG function are partly characterized by positive values of thesignλ_2_ρ(**r**) function, which is indicative to some extent of steric repulsion between the pyridine fragment of the abiraterone moiety and the phthalocyanine or porphyrin systems.

### 3.4. Structure in Solution

Finally, peculiarities of chemical bonding of AbirAc and zinc(II) 5,10,15,20-tetraphenylporphyrinate in solutions and the potential for drug release of a complex were studied in detail, with the example of complex **13**, using NMR spectroscopy known to be sensitive to molecular dynamics in solution (Figures S10–S52, Supporting Information). The complex and coordination of pyridine nitrogen by zinc(II) atoms were found to remain stable in solutions of low-polar solvents, for example, in organochlorides. In polar solvents such as DMSO, AbirAc fully splits away from the coordination sphere of zinc(II) and is replaced by solvent molecules which are present in solution in large numbers and ensure equilibrium during AbirAc release. The dynamics of drug release in polar solvents could not be estimated due to the speed of the process. The existence a of stable [ZnTPP(AbirAc)] complex, as well as its decomposition into two separate AbirAc and [ZnTPP] complexes, was confirmed by a series of independent NMR techniques.

**Figure 5:**
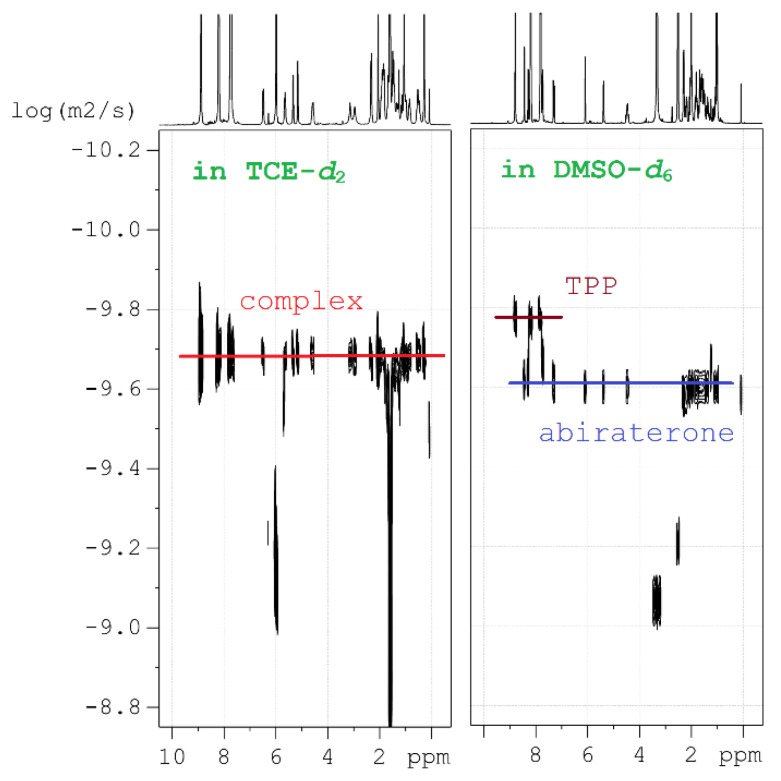
Diffusion NMR studies: representation of two fundamentally different 2D ^1^H DOSY spectra in 1,1,2,2-tetrachloroethane-*d*_2_ (TCE-*d*_2_) and DMSO-*d*_6_, one of which contains complex **14**, and the other containing free molecules of [ZnTPP] and AbirAc.

**Figure 6:**
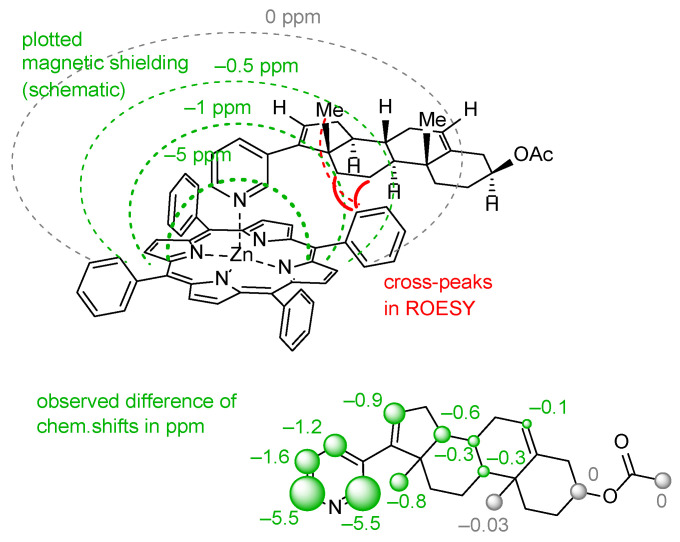
Schematic representation of the magnetic shielding of aromatic π-system in [ZnTPP] framework in complex **14**, and its impact on the chemical shifts of signals in the abiraterone molecule.

**Figure 7:**
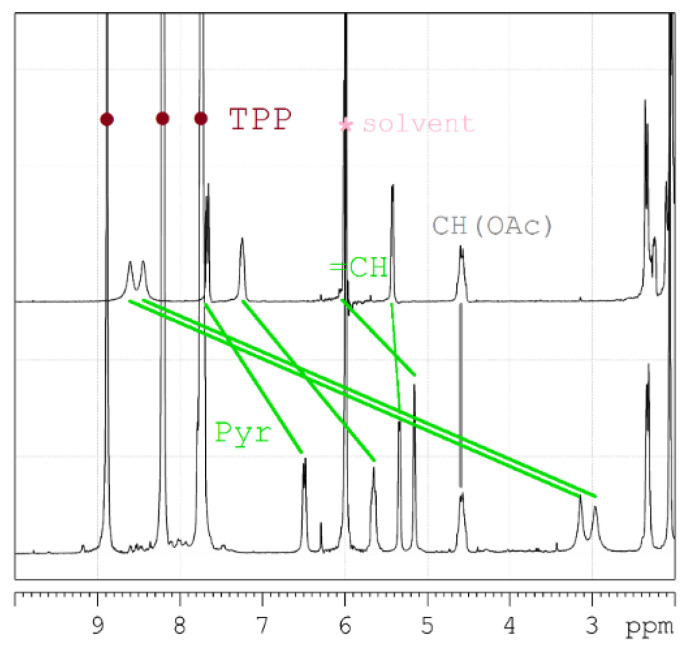
Downfield region of the ^1^H NMR spectra to demonstrate clearly the shift of the abiraterone acetate signals due to coordination with [ZnTPP] (AbirAc = top; [ZnTPP(AbirAc)] = bottom; solvent = 1,1,2,2-tetrachloroethane-*d*_2_; assignment = TOCSY, HSQC, HMBC).

(1) Diffusion-ordered NMR spectroscopy (DOSY) was applied to estimate the dynamics of molecular motion in the solutions and to estimate their molecular mass (MW). This method is well suited to analyze chemical bonding, because in low-polar solvents the [ZnTPP(AbirAc)] complex moves as a whole and has one diffusion coefficient (Figure 5). In polar solvents, AbirAc and [ZnTPP] act as two independent molecules with different diffusion coefficients because of varying size and mass (Figure 5). For complex **14**, the diffusion coefficient was compared with those of its individual components sensitive to MW increase and diffusion-slowing accompanying the complex formation (see ESI).

(2) Another method is analysis of NOE interactions between protons of abiraterone acetate and tetraphenylporphyrinate which can be found only for the [ZnTPP(AcirAc)] complex. Taking the high molecular mass of the complex as well as the relatively free rotation of abiraterone acetate along the Zn-N coordination bond, ROESY spectra should be analyzed, while intensities of NOE interactions are rather low (Figure 6).

(3) The third, less common, approach is analysis of variation in chemical shifts of signals in ^1^H NMR spectra due to the effect of strong ring electronic currents in the extended π-system of porphyrin and four phenyl rings when the AbirAc molecule partially enters the anisotropy cone of the [ZnTPP] upon coordination with zinc(II) [63]. The values of the magnetic shielding for the closest to TPP protons achieve as much as −5.5 ppm and gradually decrease to zero for the farthest atoms (Figure 6 and Figure 7). At the same time the signals corresponding to the phenyl and porphyrin protons remain unchanged upon coordination of AbirAc. This approach gives the most detailed structural information about the mutual disposition of AbirAc and [ZnTPP] in solution, because it allows detection of the closest protons for these two molecules, while the ring currents of the aromatic π-system have almost no effect on the other side of AbirAc. In solution, disposition of AbirAc towards the [ZnTPP] differs from that in a solid state. The molecule is nearly 90° shifted and free rotation dynamic is hindered (Figure 7).

## 4. Conclusions

In summary, self-assembly of abiraterone acetate with transition metal salts was studied, and its first complexes with transition metals were obtained. Energies of coordination bonds for nine complexes were estimated from the theoretical charge density of isolated molecules. The bond between a metal ion and the nitrogen atom of the 3-pyridyl ring of abiraterone acetate is stronger than bonds with solvent (acetonitrile or water) molecules and anions (nitrate or acetate) for a given cation. Thus, it is not surprising that abiraterone acetate readily replaces solvent molecules in the coordination sphere of metal ions by the nitrogen atom of the pyridyl ring upon crystallization. The strength of M–N(pyridyl) bond complexes increases in the following manner: E(Ag^I^–N)≈E(Cu^II^–N) ≈ E(Cd^II^–N) ≈ E(Zn^II^–N) < E(Ni^II^–N) < E(Fe^II^–N) < E(Co^II^–N). The bonding in phthalocyaninates and porphyrinates can be additionally supported by weak intramolecular interactions between the steroid fragment of abiraterone and the π-system of the macrocycle. Nevertheless, a zinc(II) porphyrinate-containing complex with abiraterone acetate remains stable only in low-polar solvents, while drug release was observed in polar solvents which makes this family of compounds prospective as metallopharmaceuticals.

## Figures and Tables

**Table 1:** Reaction mixtures attested for complex formation.

	CH_3_CN	THF	Et_2_O	EtOH	CH_2_Cl_2_	Acetone
AgNO_3_	**1** ^a^	–	–	**2** ^a^	↓	↓
Cu(NO_3_)_2_·6H_2_O	**3** ^c^	↓	↓	**4** ^c^	↓	–
Cu(AcO)_2_·H_2_O	**5** ^b^	**6** ^b^	↓	↓	↓	↓
CuCl_2_·2H_2_O	–	–	↓	↓	↓	↓
Co(NO_3_)_2_·6H_2_O	**7** ^c^	–	–	–	–	–
CoCl_2_·2H_2_O	–	–	↓	↓	↓	–
Ni(NO_3_)_2_·6H_2_O	**8** ^c^	–	↓	↓	↓	–
Zn(NO_3_)_2_·6H_2_O	**9** ^c^	–	–	–	–	–
Cd(NO_3_)_2_·4H_2_O	**10** ^c^	–	–	–	–	–
Fe(AcO)_2_	↓	–	–	–	↓	↓
FeBr_3_	–	–	–	**11** ^b^	–	–
FePC	**12** ^c^	↓	↓	–	–	↓
ZnTPP	↓	↓	↓	**13**, **14** ^d^	–	↓

Conditions: ^a^ 0.05 mmol AbirAc, 0.10 mmol AgNO_3_; ^b^ 0.05 mmol AbirAc, 0.05 mmol Cu(OAc)_2_·H_2_O, FeBr_3_; ^c^ 0.05 mmol AbirAc, 0.025 mmol d-metal substrate (Cu(II), Co(II), Ni(II), Zn(II), Cd(II), Fe(II)); ^d^ 0.05 mmol AbirAc, 0.05 mmol ZnTPP in EtOH (**13**) or MeOH (**14**). Notations: insoluble salts are denoted with a down arrow; a dash denotes the mixture where no complexes were obtained. H_2_PC = phthalocyanine and H_2_TPP = 5,10,15,20-tetraphenylporphyrine.

**Table 2:** H-bond parameters (Å, °) for solid **3**, **4**, **7**, **8**, **10**, **11**, and **14**.

Cmpd	*D*—H···*A*	*D*—H	H···*A*	*D*···*A*	*D*—H···*A*
**3**	O14—H14A···O11 ^i^	0.87	2.001	2.795(9)	152
	O14—H14B···O8 ^i^	0.86	2.026	2.761(8)	143
**4**	O1—H1A···O8	0.93	1.729	2.631(12)	164
	O1—H1B···O4 ^ii^	0.93	1.895	2.756(11)	153
	O2—H2···O11 ^i^	0.85	1.739	2.54(6)	157
	O3—H3A···O7	0.85	2.093	2.79(3)	139
	O3—H3B···O11 ^i^	0.85	1.910	2.75(3)	167
	O7—H7A···O8	0.85	2.287	3.12(3)	168
	O7—H7B··O13 ^iii^	0.85	2.147	2.78(3)	131
**7**	O1—H1A···O5A	0.87	1.928	2.785(5)	170
	O1—H1B···O4A ^iv^	0.87	1.826	2.729(9)	174
	O2—H2A···O5 ^iii^	0.85	1.984	2.771(9)	153
	O2—H2B···O4 ^v^	0.85	2.169	2.884(8)	142
**8**	O3—H3A···O7	0.81	2.163	2.90(1)	153
	O3—H3B···O6 ^vi^	0.83	2.000	2.78(1)	157
	O4—H4A···O6A ^vii^	0.85	1.970	2.75(1)	168
	O4—H4B···O7A ^ix^	0.85	1.908	2.79(1)	163
**10**	O1—H1B···O3 ^x^	0.86	2.339	2.780(8)	157
	O1—H1C···O5A	0.86	1.972	2.927(7)	126
	O2—H2A···O3A ^xi^	0.88	2.012	2.863(7)	161
	O2—H2B···O5A ^xii^	0.88	1.874	2.733(8)	163
**11**	N1—H1···O2 ^xiii^	0.88	1.906	2.668(9)	144
**14**	O3—H3A···O2 ^xiv^	0.84	2.068	2.81(4)	147

Symmetry transformations used: (i). *x*, −1 + *y*, *z*; (ii). *x*, 1 + *y*, *z*; (iii). *x*, *y*, −1 + *z*; (iv). ½ + *x*, 3/2 − *y*, 1 − *z*; (v). ½ + *x*, 3/2 − *y*, −*z*; (vi). 2 − *x*, ½ + *y*, 3/2 − *z*; (v). 1 − *x*, ½ + *y*, 3/2 − *z*; (ix). −1 + *x*, *y*, *z*; (x). −1 − *x*, ½ + *y*, 1/2 − *z*; (xi). −*x*, ½ + *y*, 1/2 − *z*; (xii). 1 + *x*, *y*, *z*; (xiii). −1/2 + *x*, 3/2 − *y*, 1 − *z*; (xiv). 1 − *x*, −1/2 + *y*, 2 − *z*.

**Table 3:** Energies of coordination bonds (kJ mol^−1^) in selected complexes ^a^.

Compd.	M	E(M–N_Py_)	E(M–N_CH3CN_)	E(M–O_An_)	E(M–O_aqua_)
**1**	Ag(I)	−109.3	−111.6	−63.7…−86.1	
**2**	Ag(I)	−97.3…−97.5		−56.5	
**5**	Cu(II)	−108.3…−108.9		−114.8…−212.7	
**7**	Co(II)	−233.7…−235.5	−211.2…−212.9		−59.3…−60.8
**8**	Ni(II)	−148.5…−149.0	−132.6…−134.2		−105.3…−106.2
**9**	Zn(II)	−122.9…−123.3		−98.2…−110.8	
**10**	Cd(II)	−100.9…−101.4	−64.4…−66.4		−56.8…−61.7
**12**	Fe(II)	−160.1…−162.8			
**13**	Zn(II)	−97.3			

^a^ Energies were estimated using formula suggested by Espinosa, Mollins, and Lecomte as E_int_ ≈ ½·V(**r**) [57], where V(**r**) is potential energy density at bcp. and N_Py_, N_Solv_, O_An_, and O_Solv_ denote atoms which belong to pyridine (Py), solvent (Solv), or anion (An) moieties.

## Data Availability

Compounds, spectra, and XRD data are available from authors.

## Supplementary Materials

The following supporting information can be downloaded at: https://www.mdpi.com/article/10.3390/pharmaceutics15092180/s1: Synthetic details and spectral data; Figures S1–S9: Rietveld refinement of powder XRD patterns; Figures S10–S52: NMR spectra; Tables S1 and S2: Crystallographic data and structure refinement details for **1**–**14**; OUT files for complexes **1, 2, 5, 7**–**10, 12,** and **13**. Crystallographic information files are available from the Cambridge Crystallographic Data Center upon request (https://ccdc.cam.ac.uk/structure, accessed on 22 August 2023; deposition numbers are 2215031–2215044).
