# Supplementary material for: Abiraterone Acetate Complexes with Biometals: Synthesis, Characterization in Solid and Solution, and the Nature of Chemical Bonding
Source: Pharmaceutics. 2023 Aug 23;15(9):2180. doi: 10.3390/pharmaceutics15092180 (PMC10535913; doi:10.3390/pharmaceutics15092180)
Supplement: Supplementary file 1 [file pharmaceutics-15-02180-s001.zip › pharmaceutics-2519258-supplementary.pdf]

Electronic Supporting Information for

Chemical bonding of abiraterone acetate with biometals

Petr A. Buikin,[a,b] Anna V. Vologzhanina,[a] Roman A. Novikov,[c] Pavel V. Dorovatovskii[d] and Alexander A. Korlyukov *[a]

[a.] *A. N. Nesmeyanov Institute of Organoelement Compounds, RAS. 119334 Moscow, Russia. E-mail: alex@xrlab.ineos.ac.ru*
[b.] *N. S. Kurnakov Institute of General and Inorganic Chemistry, RAS. 119991 Moscow, Russia.*
[c.] *N. D. Zelinsky Institute of Organic Chemistry, RAS. 119991 Moscow, Russia.*
[d.] *Kurchatov Institute, National Research Center, 123182 Moscow, Russia*

1. Synthesis	2-3
2. Powder X-ray diffraction data	4-8
3. Single crystal XRD data	9-11
4. Resulting OUT files for complexes **1**, **2**, **5**, **7-10**, **12**, **13**	12-37
5. NMR studies	38-61
6. References	62

1. Synthesis

Synthesis of [Ag(μ-AbirAc)(CH_3CN)(NO_3)]$_n$ (1). A solution of $AgNO_3$ (17 mg, 0.10 mmol) in 1 ml of acetonitrile was added to a solution of abiraterone acetate (20 mg, 0.051 mmol) in 1 ml of acetonitrile. Colorless needle crystals of **1** precipitated after 3 days of standing on air at r.t., were filtered off and dried in air. Yield: 28 mg (76% referred to pure substance). According to XRD data, precipitate contains 18% of $AgNO_3$. Details of the Rietveld refinement (Fig. S1): *P2₁2₁2₁*, a = 9.4676(2) Å, b = 14.1155(3) Å, c = 19.9466(5) Å, V = 2665.65(11) Å^3, R_{bragg} = 6.77%, R_{exp}: 2.72%, R_{wp}: 8.44%, R_p: 6.53%, GOF: 3.10.

Synthesis of [Ag(μ-AbirAc)$_2$(NO_3)] (2). A solution of $AgNO_3$ (17 mg, 0.10 mmol) in 1 ml of ethanol was added to a solution of abiraterone acetate (20 mg, 0.051 mmol) in 1 ml of ethanol. Colorless needle crystals of **2** precipitated after 3 days of standing on air at r.t., were filtered off and dried in air. Yield: 20 mg (82%). According to XRD data, precipitate contains 1.3% of $AgNO_3$. Details of the Rietveld refinement (Fig. S2): *P2₁*, a = 8.0008(6) Å, b = 9.7164(3) Å, c = 30.4075(8) Å, β = 93.844(6)°, V = 2358.5(2) Å^3, R_{bragg} = 12.87%, R_{exp}: 3.43%, R_{wp}: 11.66%, R_p: 8.06%, GOF: 3.40.

Synthesis of [Cu(AbirAc)$_2$(NO_3)$_2$][Cu(AbirAc)$_2$(NO_3)(CH_3CN)(H_2O)]NO_3·3CH_3CN (3). A solution of $Cu(NO_3)_2·6H_2O$ (7.5 mg, 0.025 mmol) in 1 ml of acetonitrile was added to a solution of abiraterone acetate (20 mg, 0.051 mmol) in 1 ml of acetonitrile. Light-blue needle crystals of **3** precipitated after 2 weeks of standing on air at r.t., were filtered off and dried in air. The precipitate contains trace amounts of crystals 3.

Synthesis of [Cu(AbirAc)$_2$(NO_3) (EtOH)(H_2O)]$_{0.33}$[Cu(AbirAc)$_2$(NO_3)(H_2O)$_2$]$_{0.67}$(NO_3) · 1.33H_2O (4). A solution of $Cu(NO_3)_2·6H_2O$ (7.5 mg, 0.025 mmol) in 1 ml of ethanol was added to a solution of abiraterone acetate (20 mg, 0.051 mmol) in 1 ml of ethanol. Blue needle crystals of **4** precipitated after 1 week of standing on air at r.t., were filtered off and dried in air. Yield: 21 mg (82%). According to XRD data precipitate contains only **4**. Details of the Rietveld refinement (Fig. S3): *P2₁*, a = 20.2112(7) Å, b = 6.2331(6) Å, c = 20.8075(9) Å, β = 97.606(3)°, V = 2598.3(3) Å^3, R_{bragg} = 9.53%, R_{exp}: 2.81%, R_{wp}: 10.37%, R_p: 7.39%, GOF: 3.69.

Synthesis of [Cu_2(AbirAc)$_2$(OAc)$_4$] (5). A solution of $Cu(OAc)_2·H_2O$ (10 mg, 0.051 mmol) in 1 ml of acetonitrile was added to a solution of abiraterone acetate (20 mg, 0.051 mmol) in 1 ml of acetonitrile. Green plate crystals of 5 precipitated after 1 hour of standing on air at r.t., were filtered off and dried in air. Yield: 25 mg (85%). According to XRD data, precipitate contains only **5**. Details of the Rietveld refinement (Fig. S4): *P2₁2₁2₁*, a = 7.4465(9) Å, b = 13.2072(14) Å, c = 59.2117(12) Å, V = 5823.3(9) Å^3, R_{bragg} = 19.36%, R_{exp}: 3.40%, R_{wp}: 12.33%, R_p: 7.37%, GOF: 3.63.

Synthesis of [Cu_2(AbirAc)$_2$(OAc)$_4$]·2THF (6). A solution of $Cu(OAc)_2·H_2O$ (10 mg, 0.051 mmol) in 0.5 ml of tetrahydrofuran was added to a solution of abiraterone acetate (20 mg, 0.051 mmol) in 0.5 ml of tetrahydrofuran. Green precipitate obtained after 2 weeks of standing on air at r.t., was filtered off and dried in air. Precipitate contains single crystals of **6**. According to XRD data, it consists of crystalline and amorphous reaction products. The crystalline phase has the lower volume than the corresponding single-crystal structure that can be indicative of partial or full desolvation of the reaction product at r.t. Details of the Pawley refinement (Fig. S5): *P2₁*, a = 41.961(3) Å, b = 9.7196(3) Å, c = 8.0979(4) Å, β = 93.515(5)°, V = 3296.4(3) Å^3, R_{bragg} = 0.52%, R_{exp}: 0.88%, R_{wp}: 3.87%, R_p: 2.19%, GOF: 4.42.

Synthesis of [Co(AbirAc)$_2$(CH_3CN)$_2$(H_2O)$_2$](NO_3)$_2$ (7). A solution of $Co(NO_3)_2·6H_2O$ (7.4 mg, 0.025 mmol) in 1 ml of acetonitrile was added to a solution of abiraterone acetate (20 mg, 0.051 mmol) in 1 ml of acetonitrile. Pale-pink plate crystals of **7** precipitated within 1 hour, were filtered off and dried in air. Yield: 24 mg. According to XRD data, precipitate contains some unidentified impurities. Details of the Rietveld refinement (Fig. S6): *P2₁2₁2₁*, a = 10.5163(5) Å, b = 42.263(2) Å, c = 6.1357(15) Å, V = 2727.0(7) Å^3, R_{bragg} = 8.44%, R_{exp}: 1.63%, R_{wp}: 8.56%, R_p: 4.79%, GOF: 5.25.

Synthesis of [Ni(AbirAc)$_2$(CH_3CN)$_2$(H_2O)$_2$](NO_3)$_2$ (8). A solution of $Ni(NO_3)_2·6H_2O$ (7.3 mg, 0.025 mmol) in 1 ml of acetonitrile was added to a solution of abiraterone acetate (20 mg, 0.051 mmol) in 1 ml of acetonitrile. Pale-blue needle crystals of **8** precipitated within 1 hour, were filtered off and dried in air. Yield: 23 mg. According to XRD data, precipitate contains some unidentified impurities. Details of the

Rietveld refinement (Fig. S7): $P2_12_12_1$, a = 6.1482(3) Å, b = 10.4962(4) Å, c = 42.1476(17) Å, V = 2719.9(2) Å^3, R_{bragg} = 8.34%, R_{exp}: 0.82%, R_{wp}: 7.17%, R_p: 4.88%, GOF: 8.78.

Synthesis of [Zn(AbirAc)$_2$(NO$_3$)$_2$] (9). A solution of $Zn(NO_3)_2·6H_2O$ (7.5 mg, 0.025 mmol) in 1 ml of acetonitrile was added to a solution of abiraterone acetate (20 mg, 0.051 mmol) in 1 ml of acetonitrile. Colorless plate crystals of **9** precipitated within 2 days, were filtered off and dried in air. According to powder XRD data, precipitate contains other crystalline phases besides **9**, one of those was $Zn(OH)(NO_3)(H_2O)$ (COD 1529912) (Fig. S8).

Synthesis of [Cd(AbirAc)$_2$(CH$_3$CN)$_2$(H$_2$O)$_2$](NO$_3$)$_2$ (10). A solution of $Cd(NO_3)_2·4H_2O$ (7.8 mg, 0.025 mmol) in 1 ml of acetonitrile was added to a solution of abiraterone acetate (20 mg, 0.051 mmol) in 1 ml of acetonitrile. Distilled water was dropwise added to a solution until its blurring. After 1 hour colorless precipitate was filtered off and dried in air. The precipitate contains trace amounts of crystals 10.

Synthesis of (AbirAcH)[FeBr$_4$] (11). A solution of $FeBr_3$ (15 mg, 0.051 mmol) in 1 ml of ethanol was added to a solution of abiraterone acetate (20 mg, 0.051 mmol) in 1 ml of ethanol. 0.10 ml of 10% HBr was dropwise added to a solution. Dark-red needle crystals of **11** precipitated within 4 days, were filtered off and dried in air. The precipitate contains trace amounts of crystals **11**.

Synthesis of [FePC(AbirAc)$_2$] (12). A solution of iron(II) phthalocyaninate (14 mg, 0.025 mmol) in 1 ml of acetonitrile was added to a solution of abiraterone acetate (20 mg, 0.051 mmol) in 1 ml of acetonitrile. Dark-violet needle crystals of **12** precipitated within 1 day, were filtered off and dried in air. Yield: 27 mg. According to XRD data, precipitate contains some unidentified impurities. Details of the Rietveld refinement (Fig. S9): *P1*, a = 9.3029(6) Å, b = 9.5883(5) Å, c = 21.1586(9) Å, V = 1715.48(18) Å^3, R_{bragg} = 6.48%, R_{exp}: 3.52%, R_{wp}: 7.53%, R_p: 5.11%, GOF: 2.14.

Synthesis of [ZnTPP(AbirAc)] (13). A solution of zinc(II) tetraphenylporphyrinate (34 mg, 0.050 mmol) in 1 ml of acetonitrile was added to a solution of abiraterone acetate (20 mg, 0.051 mmol) in 1 ml of acetonitrile. Dark-violet needle crystals of **13** precipitated within 2 days, were filtered off and dried in air.

Synthesis of [ZnTPP(AbirAc)]·MeOH (14). A solution of zinc(II) tetraphenylporphyrinate (34 mg, 0.050 mmol) in 1 ml of methanol was added to a solution of abiraterone acetate (20 mg, 0.051 mmol) in 1 ml of methanol. Dark-violet needle crystals of **14** precipitated within 2 days, were filtered off and dried in air.

2. Powder X-ray diffraction data

The powder X-ray diffraction patterns of all the samples were obtained in reflection mode. The measurements were performed with a Bruker D8 Advance diffractometer (Bregg–Brentano geometry) equipped with motorized slits and a LynxEye 1D position-sensitive detector (CuKa, Ni-filter). The measurement range was 2Ω = 3–60°. Rietveld full-profile X-ray analysis of the patterns of crystalline substances was made using TOPAS 4.2 software.[S1] The background, profile, preferred orientation, scale factor(s) and unit cell parameters were refined. The preferred orientation was taken into account with the spherical harmonics approach.[S2]

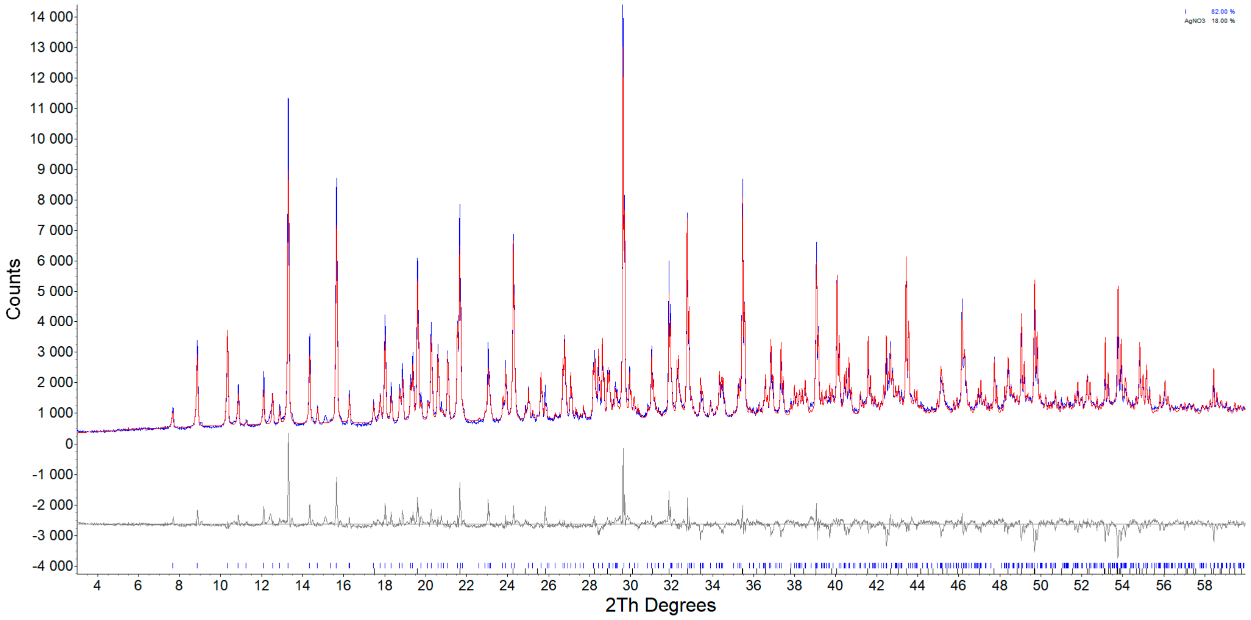

Figure S1. XRD pattern for sample of **1**. The blue line is the experimental pattern, the fuchsia line is the calculated pattern, and the grey line is the difference curve.

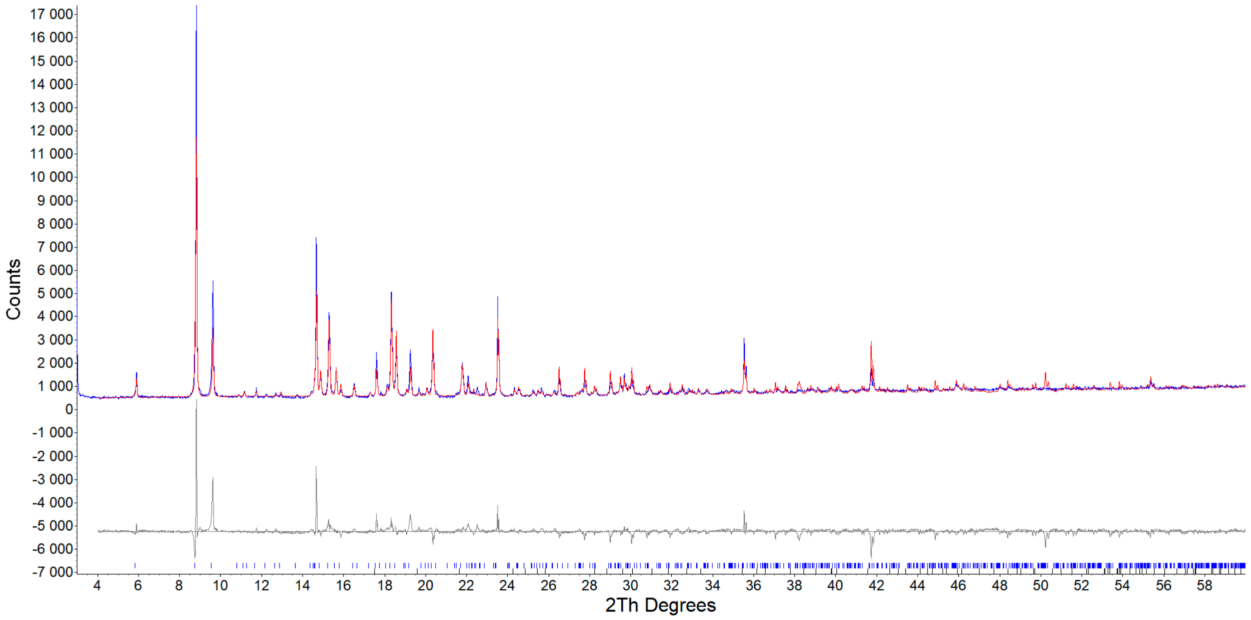

Figure S2. XRD pattern for sample of **2**. The blue line is the experimental pattern, the fuchsia line is the calculated pattern, and the grey line is the difference curve.

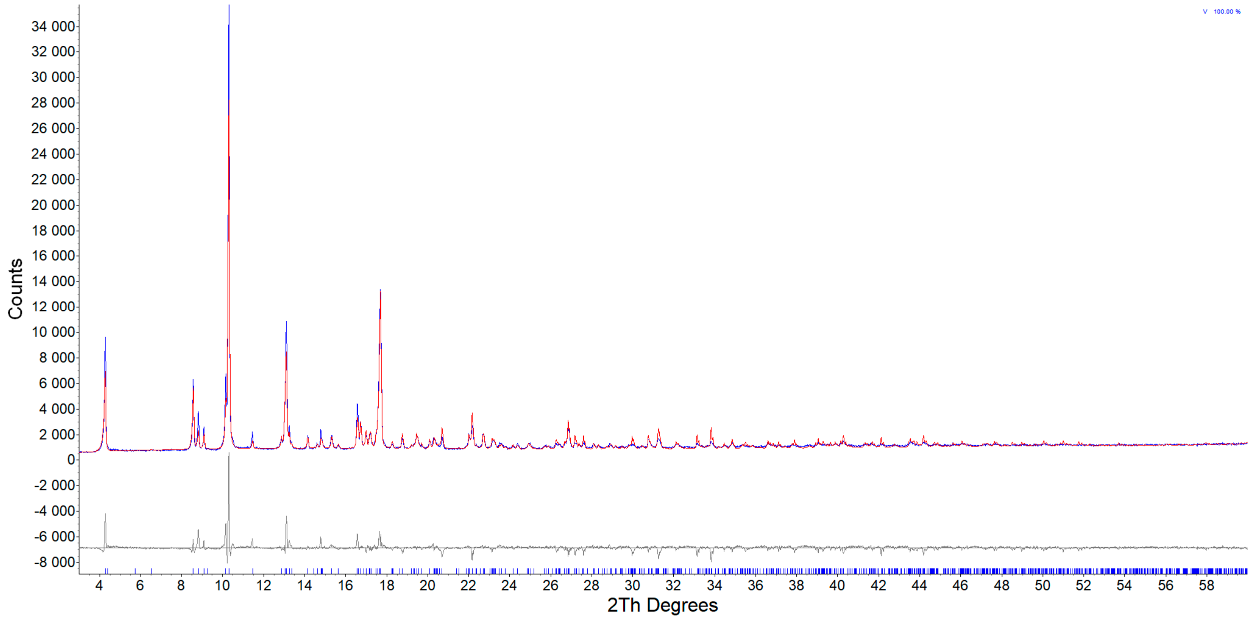

Figure S3. XRD pattern for sample of **4**. The blue line is the experimental pattern, the fuchsia line is the calculated pattern, and the grey line is the difference curve.

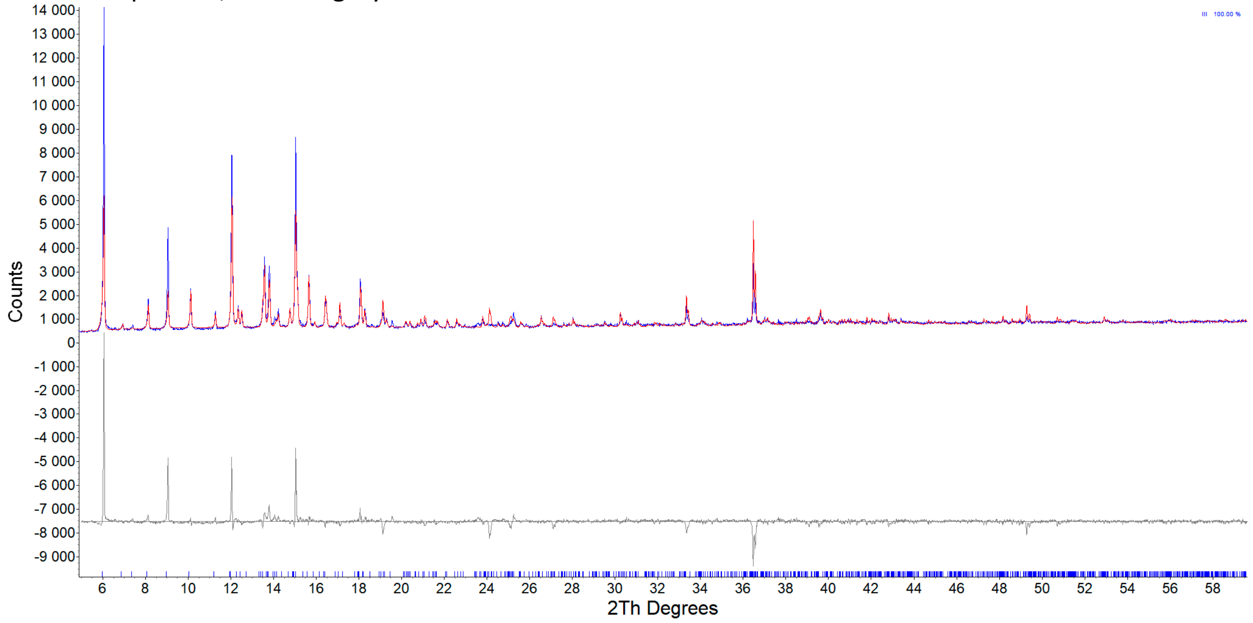

Figure S4. XRD pattern for sample of **5**. The blue line is the experimental pattern, the fuchsia line is the calculated pattern, and the grey line is the difference curve.

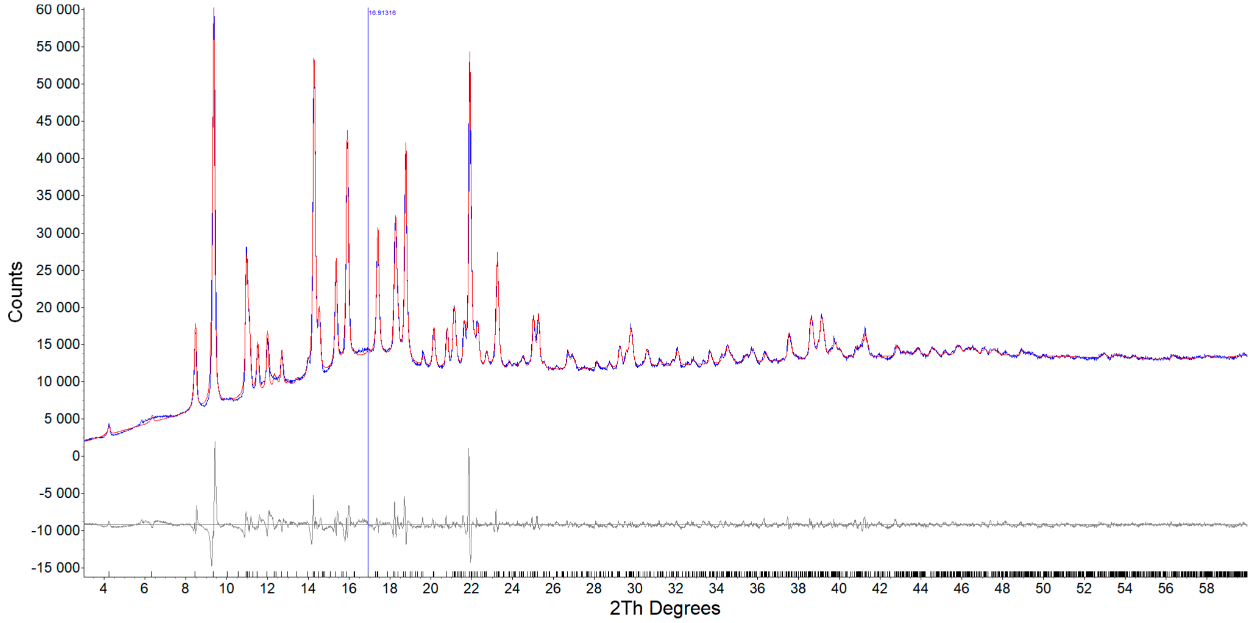

Figure S5. XRD pattern for sample of **6**. The blue line is the experimental pattern, the fuchsia line is the calculated pattern, and the grey line is the difference curve.

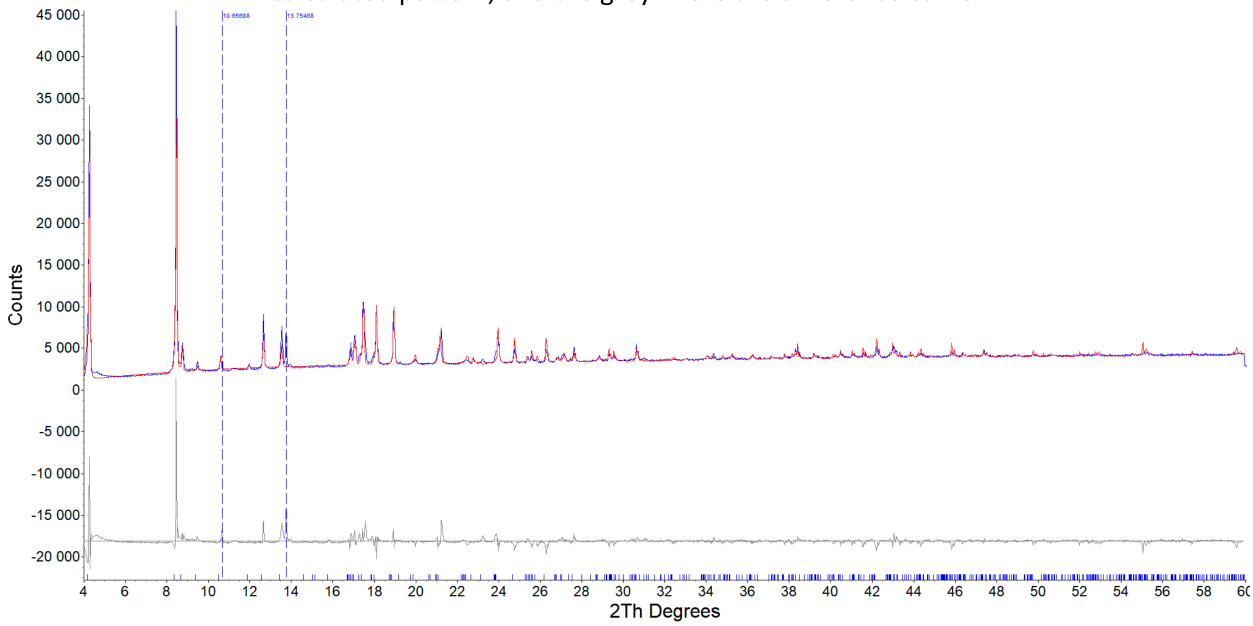

Figure S6. XRD pattern for sample of **7**. The blue line is the experimental pattern, the fuchsia line is the calculated pattern, and the grey line is the difference curve.

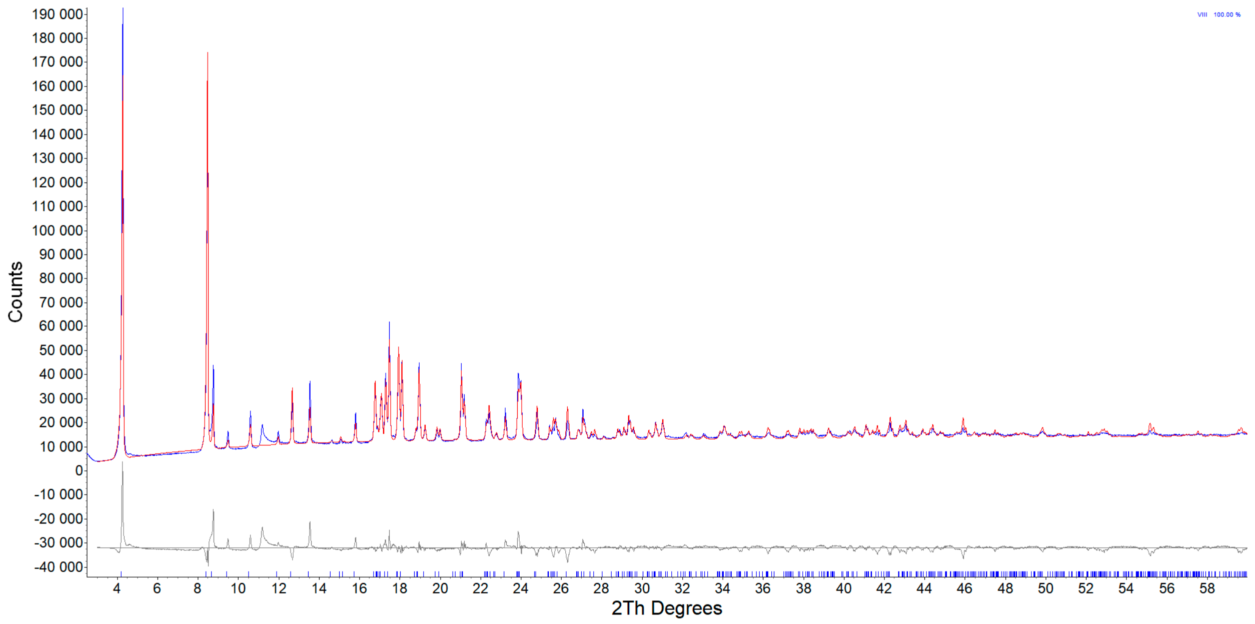

Figure S7. XRD pattern for sample of **8**. The blue line is the experimental pattern, the fuchsia line is the calculated pattern, and the grey line is the difference curve.

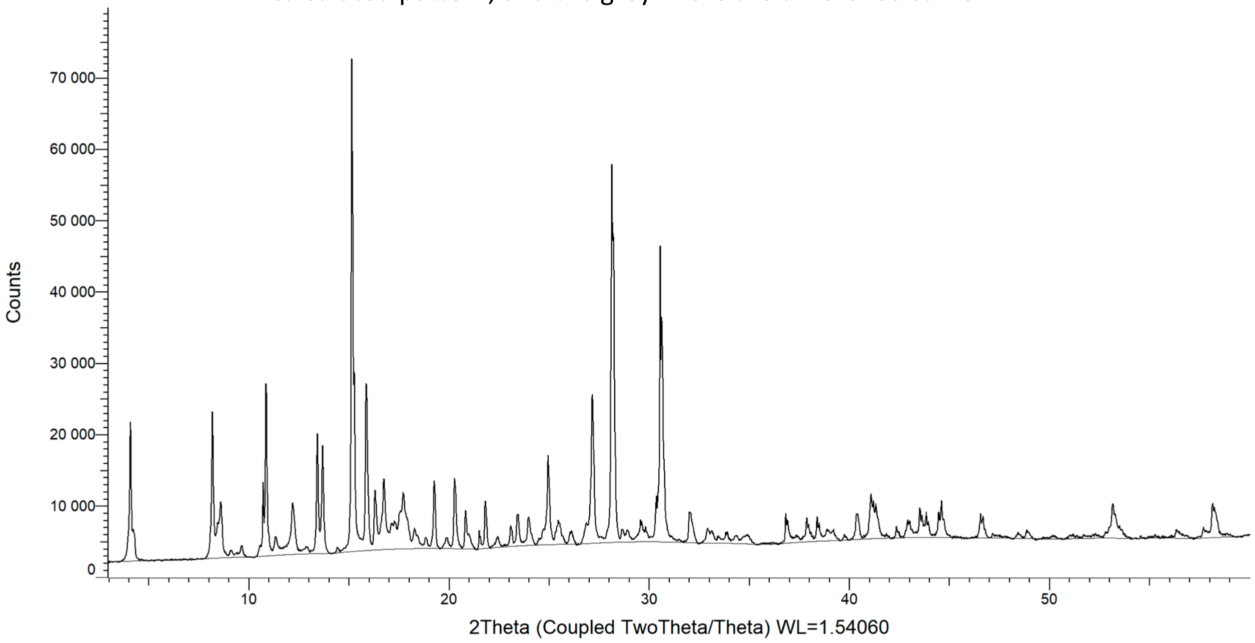

Figure S8. XRD pattern for sample of **9**.

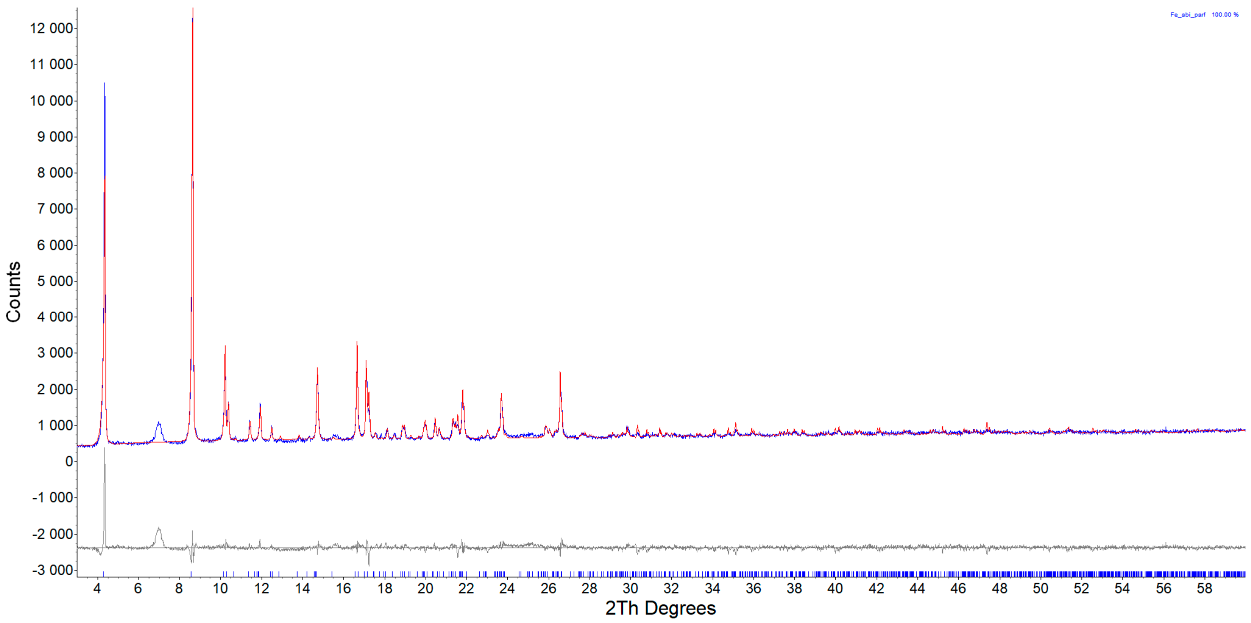

Figure S9. XRD pattern for sample of **12**. The blue line is the experimental pattern, the fuchsia line is the calculated pattern, and the grey line is the difference curve.

3. Single-crystal X-ray diffraction data

Experimental details and the results of these refinements are listed in Tables S1-S2.

Table S1. Crystallographic data and structure refinement details for **1 – 7**.

	**1**	**2**	**3**·$3CH_3CN$	**4**·$0.67H_2O$	**5**	**6**·$2C_4H_8O$	**7**
Formula	$C_{28}H_{36}AgN_3O_5$	$C_{52}H_{66}AgN_3O_7$	$C_{112}H_{146}Cu_2N_{12}O_{21}$	$C_{52.66}H_{72.66}CuN_4O_{12.67}$	$C_{60}H_{78}Cu_2N_2O_{12}$	$C_{68}H_{94}Cu_2N_2O_{14}$	$C_{56}H_{76}CoN_6O_{12}$
Fw	602.47	952.94	2123.48	1027.98	1146.32	1290.52	1084.15
Crystal system	Orthorhombic	Monoclinic	Monoclinic	Monoclinic	Orthorhombic	Monoclinic	Orthorhombic
Space group	$P2_12_12_1$	$P2_1$	$P2_1$	$P2_1$	$P2_12_12_1$	C2	$P2_12_12_1$
Z	4	2	1	2	4	2	2
a, Å	9.4165(14)	7.9668(17)	20.615(4)	20.016(9)	7.2968(2)	42.078(8)	10.322(2)
b, Å	13.7266(19)	9.534(3)	5.9310(12)	6.170(3)	13.2384(4)	9.7336(18)	42.253(8)
c, Å	20.027(2)	30.247(8)	21.667(4)	20.867(11)	58.2440(18)	8.0960(15)	6.0570(12)
, °	90	93.494(4)	95.51(3)	97.539(14)	90	93.618(6)	90
V, $Å^3$	2588.6(6)	2293.2(10)	2636.9(9)	2555(2)	5626.3(3)	3309.3(11)	2641.7(9)
d_{calc}, g cm^{-3}	1.546	1.380	1.337	1.336	1.353	1.295	1.363
, cm^{-1}	8.23	4.96	5.39	4.95	8.2	7.07	4.45
F(000)	1248	1004	1128	1094	2424	1372	1154
No measured rfls.	18638	23133	23296	11043	58743	6514	20322
No indep. rfls. [R_{int}]	7872[0.045]	13116 [0.073]	13627 [0.043]	8135 [0.106]	16724 [0.047]	6514 [0.113]	7217 [0.066]
No observed refls. [I>2 (I)]	6937	9348	11102	4420	13746	5062	6455
No parameters	338	574	771	644	695	394	399
R1	0.0366	0.0707	0.0595	0.0844	0.0417	0.0647	0.0605
wR2	0.0749	0.1353	0.1629	0.2102	0.0863	0.1640	0.1279
GOF	0.998	1.018	1.026	1.006	1.019	1.017	1.098
Residual density, e Å^{-3}	1.255/-0.572	0.987/-0.786	0.799/-0.964	0.628/-0.458	0.273/-0.272	0.532/-0.349	0.528/-0.454

Table S2. Crystallographic data and structure refinement details for **8 – 14**.

	**8**	**9**	**10**	**11**	**12**	**13**	**14·MeOH**
Formula	$C_{56}H_{76}N_6NiO_{12}$	$C_{52}H_{66}N_4O_{10}Zn$	$C_{56}H_{76}CdN_6O_{12}$	$C_{26}H_{34}Br_4FeNO_2$	$C_{84}H_{82}FeN_{10}O_4$	$C_{70}H_{61}N_5O2Zn$	$C_{71}H_{65}N_5O_3Zn$
Fw	1083.93	972.45	1137.62	768.03	1351.44	1069.60	1101.65
Crystal system	Orthorhombic	Monoclinic	Orthorhombic	Orthorhombic	Triclinic	Monoclinic	Monoclinic
Space group	$P2_12_12_1$	$P2_1$	$P2_12_12_1$	$P2_12_12_1$	P1	$P2_1$	$P2_1$
Z	2	2	2	4	1	2	2
a, Å	6.0510(12)	6.2140(12)	6.0810(12)	7.5016(9)	9.2491(11)	12.0929(3)	10.7755(10)
b, Å	10.311(2)	11.420(2)	10.385(2)	10.3861(13)	9.5269(11)	18.6912(5)	18.941(3)
c, Å	42.195(8)	33.266(7)	42.460(9)	37.016(5)	20.910(2)	13.2073(4)	14.6843(17)
a, °	90	90	90	90	98.988(4)	90	90
b, °	90	94.75(3)	90	90	93.394(4)	114.189(1)	108.845(4)
g, °	90	90	90	90	111.854(4)	90	90
V, $Å^3$	2632.6(9)	2352.6(8)	2681.4(9)	2884.0(6)	1675.2(3)	2723.15(13)	2836.3(6)
d_{calc}, g cm^{-3}	1.367	1.373	1.409	1.769	1.340	1.304	1.290
m, cm^{-1}	4.92	6.6	5.34	60.88	2.89	0.51	4.87
No measured rfls.	31275	22502	22947	36160	14903	47573	29479
No indep. rfls. [R_{int}]	7133 [0.055]	12933 [0.052]	7357 [0.055]	7781 [0.116]	12584 [0.038]	19033 [0.044]	11191 [0.113]
No observed refls. [I>2s(I)]	5923	11976	6941	5500	7173	16009	7206
R1	0.0595	0.0411	0.0401	0.0545	0.0585	0.0392	0.0723
wR2	0.1428	0.1034	0.0937	0.1031	0.1162	0.0870	0.1488
GOF	1.110	1.031	1.082	1.044	0.910	0.981	1.016
Residual density, e $Å^{-3}$	0.604/-0.578	0.550/-0.861	0.462/-0.724	0.570/-0.727	0.434/-0.581	0.649/-0.572	0.423/-0.611

4. Resulting OUT files for complexes 1, 2, 5, 7-10, 12, 13

1

CARTESIAN COORDINATES (ANGSTROEM)

Ag	7.923737	12.851101	5.734958
O	6.253568	9.335681	17.833991
O	4.755312	7.791972	18.481250
O	7.280183	14.481505	7.455403
O	7.549713	12.493693	8.240966
O	7.021958	14.110807	9.564660
N	6.738931	10.775498	5.434140
N	7.586913	13.893384	3.602346
N	7.270485	13.707820	8.443885
C	6.186025	10.305023	4.320506
H	6.298127	10.910679	3.427669
C	5.508038	9.096296	4.293866
H	5.048501	8.753188	3.375041
C	5.427529	8.347189	5.451704
H	4.889503	7.406409	5.453355
C	6.032988	8.805787	6.624206
C	6.653958	10.051814	6.547752
H	7.106521	10.500587	7.419137
C	6.053496	8.024077	7.860123
C	5.861881	6.698396	7.927674
H	5.667179	6.056778	7.075386
C	6.032522	6.165552	9.317742
H	7.019787	5.695913	9.434719
H	5.292320	5.405577	9.581420
C	5.900690	7.447588	10.132237
H	4.821604	7.660287	10.161966
C	6.491429	8.543899	9.228754
C	8.028188	8.540473	9.239986
H	8.418498	7.557930	8.965012
H	8.434259	8.803076	10.215851
H	8.422093	9.263826	8.525177
C	5.936334	9.867109	9.748870
H	4.844189	9.843689	9.659502
H	6.285947	10.735991	9.190084
C	6.343254	10.067350	11.208492
H	7.410288	10.296439	11.234735
H	5.848274	10.967887	11.578094
C	6.035475	8.893143	12.144480
H	4.943333	8.870337	12.281838
C	6.390443	7.517364	11.559634
H	7.477471	7.378748	11.580655
C	5.765472	6.433020	12.420826
H	4.689089	6.365826	12.202582
H	6.178827	5.453235	12.155845
C	5.968591	6.682103	13.875879
H	5.772728	5.852262	14.551164
C	6.639191	9.076563	13.556503

C	8.149705	9.340279	13.496941
H	8.369545	10.337601	13.112058
H	8.653215	8.607514	12.863277
H	8.609222	9.274653	14.482183
C	5.935729	10.267380	14.237347
H	6.211646	11.190309	13.721974
H	4.853414	10.145167	14.110160
C	6.243293	10.413226	15.718258
H	7.305573	10.623229	15.870991
H	5.687504	11.257342	16.136173
C	5.884476	9.148354	16.456384
H	4.807151	8.965097	16.415505
C	6.623051	7.958732	15.871765
H	7.691279	8.104356	16.074882
H	6.314212	7.042517	16.377405
C	6.386077	7.834817	14.393633
C	5.616991	8.588440	18.743871
C	6.127074	8.880295	20.122959
H	7.177693	8.590498	20.191506
H	5.543336	8.324875	20.852570
H	6.070933	9.950651	20.326790
C	7.323078	13.903579	2.485994
C	6.992187	13.902880	1.080029
H	7.542133	14.693349	0.567424
H	5.922468	14.072650	0.950186
H	7.268369	12.932275	0.664350
C	10.791272	12.231076	5.421743
O	10.231282	10.059383	2.403878
O	8.414092	11.130964	1.635287
N	10.957313	11.101515	14.908238
C	10.010008	11.634645	15.670236
H	9.869696	11.187932	16.651005
C	9.213957	12.692902	15.256473
H	8.435330	13.077869	15.904262
C	9.421630	13.225596	13.999461
H	8.795465	14.027960	13.626410
C	10.431503	12.707468	13.186090
C	11.150352	11.626429	13.704910
H	11.936582	11.167521	13.115392
C	10.698699	13.284009	11.869309
C	10.533926	14.576016	11.556552
H	10.238218	15.345766	12.260099
C	10.821642	14.856458	10.111443
H	11.818229	15.300831	9.978554
H	10.099727	15.547355	9.669107
C	10.714825	13.445257	9.542507
H	9.637226	13.239279	9.526729
C	11.258531	12.539892	10.659799
C	12.791221	12.565075	10.742616
H	13.155875	13.587038	10.862321
H	13.261928	12.144048	9.853243
H	13.146796	11.991087	11.600417
C	10.739415	11.136472	10.375200

H	9.646432	11.149371	10.442848
H	11.097426	10.409339	11.110309
C	11.160229	10.685775	8.975361
H	12.243218	10.533846	8.962878
H	10.718829	9.704899	8.785161
C	10.756959	11.658026	7.861051
H	9.659971	11.704991	7.861224
C	11.217831	13.093462	8.157476
H	12.313087	13.137854	8.128694
C	10.670052	14.074233	7.127037
H	9.687499	14.447334	7.439581
H	11.306035	14.965958	7.086090
C	10.557561	13.508422	5.752174
H	10.330923	14.212362	4.952835
C	11.161374	11.176501	6.449995
C	12.667723	10.903430	6.350379
H	12.950297	10.047549	6.966542
H	13.244527	11.769250	6.680004
H	12.972443	10.680748	5.326730
C	10.376429	9.892142	6.128436
H	10.716167	9.084551	6.779861
H	9.324467	10.068961	6.373658
C	10.469720	9.434109	4.682950
H	11.496546	9.155491	4.425930
H	9.851857	8.544409	4.533929
C	10.012732	10.528339	3.750781
H	8.945726	10.721689	3.876970
C	10.795868	11.806999	3.977219
H	11.827551	11.623500	3.652407
H	10.395825	12.609618	3.353778
C	9.381443	10.431096	1.452931
C	9.789029	9.877915	0.121673
H	10.680678	10.405702	-0.224938
H	8.985307	10.016019	-0.597343
H	10.048808	8.822838	0.208831

2

CARTESIAN COORDINATES (ANGSTROEM)

Ag	-11.237553	-20.008924	-9.669650
O	-17.230822	-19.628449	-23.432870
O	-1.779651	-20.033558	3.646065
O	-3.193451	-18.291224	3.547186
O	-15.239854	-19.058864	-9.751546
N	-14.058706	-19.337497	-9.727628
O	-19.341037	-19.526056	-22.670053
O	-13.656805	-20.440100	-9.267379
N	-10.756689	-20.436012	-7.454250
N	-10.545025	-19.880333	-11.867034
O	-13.190376	-18.532860	-10.165939
C	-8.350872	-19.750799	-2.526164

H	-8.538457	-20.786984	-2.221197
H	-9.324805	-19.272681	-2.671450
C	-11.252703	-19.037560	-14.007175
C	-14.456228	-17.817675	-17.789751
H	-13.907408	-16.981942	-18.239851
C	-12.479744	-18.424046	-16.282185
C	-14.215095	-19.094627	-18.608723
H	-14.917835	-19.834897	-18.197335
C	-7.597578	-19.016599	-1.415640
H	-8.165458	-19.117634	-0.487622
H	-7.595486	-17.949734	-1.647128
C	-11.702146	-20.969698	-5.340581
H	-12.587183	-21.196590	-4.759660
C	-7.387943	-18.321037	-4.351666
H	-7.021428	-17.620483	-3.601362
H	-8.359918	-17.958681	-4.696856
H	-6.702970	-18.291096	-5.201305
C	-10.046470	-19.470024	-14.553965
H	-9.830425	-19.309171	-15.601628
C	-6.158719	-19.496015	-1.184357
H	-6.220658	-20.512765	-0.767290
C	-9.109979	-20.096501	-13.747839
H	-8.165468	-20.436757	-14.153876
C	-9.331109	-20.647617	-5.530345
C	-13.970578	-18.057430	-16.378308
H	-14.499746	-18.955398	-16.025409
C	-5.407703	-18.651406	-0.126635
C	-12.804461	-19.670566	-18.432902
H	-12.089707	-19.065054	-18.994442
H	-12.763779	-20.667310	-18.879262
C	-12.320953	-19.766967	-16.985383
H	-12.903047	-20.518506	-16.440926
H	-11.284371	-20.116090	-16.986412
C	-14.611086	-18.915242	-20.094420
C	-15.937023	-17.476856	-17.808504
H	-16.472500	-18.132978	-17.106677
H	-16.095225	-16.460333	-17.432355
C	-6.062400	-18.897554	1.247658
H	-7.071778	-18.479080	1.248550
H	-6.170376	-19.980089	1.384450
C	-12.302541	-18.364890	-14.768063
C	-3.888295	-18.897076	2.446353
H	-3.911516	-19.978076	2.611013
C	-5.351357	-19.645055	-2.482836
H	-5.115866	-18.652302	-2.884442
C	-9.392051	-20.279600	-12.408668
H	-8.683068	-20.762505	-11.745948
C	-3.955609	-19.088951	-0.043106
C	-14.177731	-17.027666	-15.272316
H	-13.915792	-16.008268	-15.588383
H	-15.205885	-16.987187	-14.904919
C	-13.216162	-17.536513	-14.240857
H	-13.262492	-17.243884	-13.197894

C	-11.818953	-20.719071	-6.697020
H	-12.776460	-20.735759	-7.208311
C	-16.727023	-19.649706	-22.087815
H	-17.420669	-20.230236	-21.473074
C	-4.045562	-20.365044	-2.189687
H	-4.237007	-21.444135	-2.093623
H	-3.362083	-20.266997	-3.040259
C	-5.627159	-20.838972	-4.835129
H	-5.076835	-20.027391	-5.331531
H	-4.943868	-21.689121	-4.771879
C	-10.451942	-20.933746	-4.751713
H	-10.338727	-21.147501	-3.696666
C	-7.529611	-19.749996	-3.808504
C	-5.286827	-18.331536	2.425918
H	-5.229441	-17.239882	2.368669
H	-5.797729	-18.572539	3.361872
C	-11.445711	-19.290521	-12.645569
H	-12.370349	-19.002714	-12.156598
C	-3.170939	-18.602097	1.141868
H	-2.182541	-19.063704	1.147865
H	-3.025988	-17.515995	1.085708
C	-15.954395	-18.213353	-20.196025
C	-16.628152	-18.238369	-21.538522
H	-16.060541	-17.642737	-22.264429
H	-17.623612	-17.798533	-21.462753
C	-15.369196	-20.306625	-22.123616
H	-14.732110	-19.768723	-22.832902
H	-15.462919	-21.330249	-22.495983
C	-13.563859	-18.098930	-20.863614
H	-13.327411	-17.171575	-20.338028
H	-13.923289	-17.822815	-21.855128
H	-12.639044	-18.662934	-21.001564
C	-14.752696	-20.310845	-20.734167
H	-15.375605	-20.926062	-20.074288
H	-13.772195	-20.791451	-20.777581
C	-18.560816	-19.559293	-23.583639
C	-5.454783	-17.154330	-0.459831
H	-5.139819	-16.972199	-1.489228
H	-4.787139	-16.582750	0.185202
H	-6.458425	-16.745207	-0.327230
C	-6.186894	-20.416406	-3.479228
H	-6.459709	-21.359636	-2.981574
C	-2.150548	-18.965000	4.052697
C	-3.379613	-19.868971	-0.953930
H	-2.345389	-20.167238	-0.798801
C	-16.536474	-17.606806	-19.164919
H	-17.518582	-17.164078	-19.313714
C	-11.572703	-17.354192	-16.907175
H	-11.714627	-17.265869	-17.984563
H	-10.519367	-17.586299	-16.734887
H	-11.764423	-16.375871	-16.462681
C	-9.556726	-20.396252	-6.882274
H	-8.719133	-20.138950	-7.521776

C	-1.521472	-18.204749	5.180067
H	-2.270312	-17.969185	5.937900
H	-1.127059	-17.257311	4.807215
H	-0.718062	-18.794645	5.613599
C	-6.896164	-21.152090	-5.574502
H	-6.927473	-21.742426	-6.483717
C	-7.968918	-20.605141	-4.992564
C	-18.930955	-19.531490	-25.035466
H	-18.515901	-18.635789	-25.501930
H	-20.013282	-19.533069	-25.135414
H	-18.501910	-20.393726	-25.548423

5

CARTESIAN COORDINATES (ANGSTROEM)

Cu	3.175773	4.825470	18.607284
Cu	2.464789	4.640649	16.120512
O	5.080690	4.803220	17.727251
O	3.096484	6.732977	18.373305
O	1.076082	4.813876	18.863150
O	3.191694	2.902994	18.636580
O	3.166786	2.673797	16.407258
O	0.734343	4.109717	16.763329
O	1.952438	6.647014	16.446480
O	4.255751	5.180114	15.676029
O	9.059730	13.323793	29.596727
O	11.134720	13.221422	28.742732
O	4.028818	9.758746	2.059831
O	5.740396	11.130158	2.545026
N	3.610112	4.966161	20.730412
N	1.891741	4.355189	14.043395
C	3.770019	3.865286	21.462524
H	3.757600	2.929647	20.916183
C	3.938359	3.921775	22.833311
H	4.074548	3.009621	23.400795
C	3.917730	5.153999	23.462866
H	4.030042	5.208774	24.537430
C	3.732196	6.313974	22.712674
C	3.604255	6.146709	21.330929
H	3.501582	7.003497	20.674824
C	3.675950	7.654468	23.292937
C	3.002166	8.690345	22.773568
H	2.378297	8.636930	21.888880
C	3.168590	9.941233	23.585706
H	2.293511	10.120047	24.226062
H	3.292989	10.835358	22.969342
C	4.417006	9.586181	24.387803
H	5.249007	9.690937	23.675186
C	4.295636	8.071502	24.623439
C	3.292825	7.737032	25.737946
H	3.623120	8.093394	26.713829
H	3.143131	6.658726	25.818622

H	2.318741	8.183262	25.529876
C	5.693126	7.571901	24.966460
H	6.347815	7.743181	24.105043
H	5.711182	6.496081	25.161716
C	6.233293	8.304716	26.194947
H	5.681424	7.960022	27.072131
H	7.268828	7.995268	26.355496
C	6.170911	9.835278	26.115776
H	6.899843	10.146501	25.352120
C	4.818538	10.367658	25.618148
H	4.062263	10.243254	26.401913
C	4.942047	11.850911	25.310832
H	5.444907	11.982482	24.341339
H	3.948191	12.294065	25.184568
C	5.689982	12.593748	26.362773
H	5.624051	13.679303	26.338747
C	6.413426	12.025460	27.324036
C	6.615258	10.523374	27.429068
C	5.825429	10.001889	28.637400
H	6.009022	10.608137	29.524678
H	6.105140	8.975906	28.884838
H	4.750937	10.030316	28.446033
C	8.118471	10.254559	27.639207
H	8.272615	9.190801	27.834911
H	8.641860	10.480402	26.702825
C	8.751897	11.065273	28.758115
H	8.306977	10.811538	29.725878
H	9.819400	10.844068	28.820895
C	8.552669	12.542105	28.503091
H	9.092387	12.832975	27.597586
C	7.080489	12.871144	28.371734
H	6.951354	13.929114	28.133854
H	6.614589	12.706450	29.350885
C	10.373558	13.586992	29.598280
C	10.754105	14.390037	30.804903
H	10.142457	15.291467	30.864925
H	11.807843	14.650987	30.751137
H	10.562388	13.806016	31.707327
C	0.713791	3.813405	13.739650
H	0.059554	3.607044	14.578388
C	0.354834	3.538907	12.433305
H	-0.612992	3.103943	12.217104
C	1.251415	3.818818	11.417055
H	0.988155	3.593327	10.392247
C	2.494699	4.370916	11.720567
C	2.745568	4.634206	13.070129
H	3.674023	5.099049	13.381639
C	3.508734	4.685961	10.716185
C	4.831467	4.693240	10.932725
H	5.303327	4.412352	11.867203
C	5.604611	5.096943	9.711429
H	6.027475	4.221303	9.199438
H	6.442583	5.760858	9.938845

C	4.504386	5.774747	8.899866
H	4.357557	6.751138	9.385855
C	3.236352	4.975838	9.243236
C	3.177574	3.633770	8.498415
H	3.082155	3.761956	7.420017
H	2.325697	3.038264	8.832250
H	4.077004	3.045317	8.688034
C	2.051716	5.862651	8.882063
H	2.080739	6.759551	9.510379
H	1.093948	5.373906	9.081470
C	2.109030	6.250697	7.404205
H	1.303141	6.960161	7.200805
H	1.882306	5.365187	6.806484
C	3.441994	6.855038	6.945045
H	3.530705	7.839832	7.428519
C	4.667989	6.063378	7.424725
H	4.741561	5.123118	6.865825
C	5.927572	6.871876	7.160945
H	6.028603	7.652128	7.929741
H	6.813447	6.237436	7.273926
C	5.933326	7.501768	5.811730
H	6.881330	7.901115	5.458736
C	4.871669	7.599190	5.015792
C	3.486420	7.126743	5.421873
C	3.133055	5.871947	4.612157
H	3.334614	6.013511	3.550158
H	2.075510	5.618074	4.708691
H	3.725316	5.015307	4.939807
C	2.483211	8.251933	5.097937
H	1.466274	7.891617	5.270587
H	2.651018	9.072012	5.805947
C	2.585143	8.799337	3.683571
H	2.342198	8.027098	2.946818
H	1.865109	9.609672	3.541992
C	3.982171	9.305453	3.422202
H	4.213068	10.154313	4.071901
C	5.003611	8.205822	3.647636
H	6.011534	8.600622	3.512029
H	4.842136	7.445092	2.873709
C	4.961526	10.670853	1.753093
C	4.896292	11.030632	0.299844
H	5.121437	10.149900	-0.304937
H	5.614483	11.818193	0.087056
H	3.888229	11.355696	0.038125
C	2.440107	7.243604	17.418900
C	2.225966	8.736286	17.519406
H	1.408690	8.911841	18.223107
H	1.954164	9.149113	16.550835
H	3.118416	9.221577	17.912394
C	0.365369	4.380705	17.944558
C	-1.103475	4.151722	18.217001
H	-1.674631	4.911999	17.680031
H	-1.311413	4.223962	19.281635

H	-1.405121	3.177455	17.832340
C	3.277223	2.237361	17.562715
C	3.562980	0.765009	17.748378
H	3.379718	0.220411	16.825356
H	2.956902	0.364275	18.560406
H	4.612728	0.655861	18.029982
C	5.192706	5.066300	16.520091
C	6.582476	5.254094	15.956882
H	7.295900	5.441975	16.755678
H	6.590040	6.066282	15.230961
H	6.861952	4.334793	15.436779

7

CARTESIAN COORDINATES (ANGSTROEM)

Co	15.879558	20.926957	10.410810
O	16.708810	20.877837	8.238561
H	17.416209	20.259680	8.029185
H	16.061123	20.777953	7.533670
O	15.024120	20.978607	12.579767
H	15.636143	21.103278	13.312089
H	14.270290	21.545315	12.771408
O	10.055754	34.384491	7.853241
O	9.532668	33.445574	5.883127
O	13.619846	9.383321	15.905231
O	13.753042	8.270644	13.961671
N	17.580471	20.238492	11.102388
C	17.579179	19.318984	12.072292
H	16.608210	19.021207	12.437094
C	18.736560	18.752044	12.602715
C	19.945956	19.184936	12.046526
H	20.878850	18.750948	12.384649
C	19.949486	20.144351	11.054678
H	20.877030	20.491169	10.617563
C	18.747224	20.656339	10.602828
H	18.705786	21.410301	9.827493
N	15.261744	19.119176	10.176624
N	16.506065	22.727855	10.655473
C	15.044360	11.174508	16.518790
H	15.668809	10.501818	17.109205
H	14.230865	11.507297	17.174557
C	18.165223	15.742729	14.747566
H	18.624679	15.161940	13.933624
C	12.347643	29.798281	4.885473
H	12.873896	30.511739	4.256722
C	14.425962	10.427366	15.355235
H	15.205707	9.970532	14.738288
C	15.176096	13.314510	15.095574
C	13.600337	11.365829	14.502866
H	13.174160	10.817026	13.661396
H	12.767284	11.747848	15.101753
C	11.163486	30.143537	5.384020

C	13.077439	24.176172	7.254377
C	12.088034	27.451073	5.719581
H	11.424220	27.080604	4.930500
C	17.227409	14.795030	15.458954
H	16.680122	15.328352	16.244263
C	12.000172	25.221274	6.975632
C	19.339294	16.396220	15.470707
H	20.173215	15.715670	15.654360
H	19.059329	16.815053	16.446748
C	9.147742	28.703180	5.531325
H	8.433569	28.210313	6.194214
H	9.452476	27.995392	4.757464
H	8.611753	29.508484	5.030111
C	10.155091	32.228222	6.301752
H	11.037701	32.481566	6.896532
C	12.994582	23.122449	8.257497
C	11.790342	22.628633	8.765492
H	10.848703	23.014975	8.401716
C	11.802792	21.633851	9.726296
H	10.879062	21.235379	10.125867
C	13.009324	21.138683	10.176252
H	13.060434	20.352482	10.917784
N	14.175385	21.612590	9.716526
C	14.161256	22.577789	8.796361
H	15.125084	22.956605	8.482636
C	13.019020	28.487854	5.111877
H	13.887760	28.632183	5.772332
H	13.429584	28.118152	4.165755
C	13.360225	8.339067	15.094988
C	10.358403	29.241755	6.304710
C	17.111268	12.505671	16.465396
H	17.538121	11.753858	17.123762
C	14.472111	12.500175	13.991598
H	13.861675	13.170302	13.379258
H	15.239796	12.078413	13.331714
C	17.992304	13.652229	16.106956
H	18.783652	13.306042	15.425736
H	18.513254	14.006823	17.002728
C	19.685258	17.486792	14.507894
H	20.630683	18.017117	14.536739
C	9.874820	30.077319	7.507715
H	9.180925	29.482796	8.107349
H	10.739595	30.293251	8.146643
C	10.989534	24.610528	5.994101
H	10.432654	23.790037	6.452553
H	11.488316	24.210399	5.109864
H	10.257610	25.343654	5.659248
C	13.892238	25.518169	5.497243
H	13.506599	25.219191	4.513573
H	14.820590	26.063446	5.312867
C	16.610016	16.295587	12.934880
H	17.273258	15.801321	12.215485
H	16.046980	17.056285	12.381921

C	15.617964	15.289789	13.521256
H	14.848403	15.839385	14.068277
H	15.099344	14.788412	12.699390
C	10.561540	31.478376	5.049992
H	9.667712	31.352305	4.427695
H	11.269868	32.071397	4.468558
C	18.680933	17.746984	13.655690
C	10.479779	27.081468	7.681768
H	10.066180	27.583554	8.558493
H	9.619313	26.713204	7.119643
C	14.135542	14.128035	15.876544
H	13.494841	13.477511	16.470918
H	13.480443	14.693773	15.209556
H	14.611929	14.822862	16.571233
C	8.864912	35.667884	6.207857
H	9.312255	35.945677	5.252304
H	8.942400	36.490268	6.913955
H	7.814271	35.438214	6.019584
C	11.299455	25.880813	8.156736
H	12.056135	26.207247	8.880124
H	10.633533	25.191172	8.685578
C	14.083655	24.322094	6.377338
H	14.921596	23.640121	6.270704
C	9.210907	31.392522	7.136762
H	8.290474	31.223247	6.568968
H	8.939792	31.938430	8.042289
C	14.928744	18.031406	10.045825
C	12.872280	26.305285	6.316693
H	13.451711	26.739233	7.146539
C	17.458740	16.913967	14.039732
C	14.508898	16.661199	9.889017
H	15.386132	16.017011	9.805810
H	13.896916	16.559938	8.990887
H	13.927003	16.355104	10.760566
C	15.857268	12.347284	16.050232
C	16.857599	23.806191	10.811700
C	9.550389	34.461593	6.765868
C	16.237970	14.223614	14.432817
H	16.838704	13.563576	13.789393
C	16.595143	17.742930	15.001470
H	15.779377	17.160218	15.427004
H	16.143240	18.598152	14.489970
H	17.191452	18.132789	15.827622
C	11.255003	28.098099	6.835799
H	11.988225	28.578738	7.501603
C	12.541623	7.304776	15.798702
H	13.100044	6.911543	16.650384
H	12.301676	6.499180	15.110037
H	11.626931	7.752833	16.190130
C	17.296160	25.166775	10.999444
H	18.105210	25.392791	10.302256
H	17.655383	25.303130	12.021150
H	16.464096	25.849421	10.816657

8

CARTESIAN COORDINATES (ANGSTROEM)

Ni	3.004851	4.974178	31.502108
O	-2.006740	6.620277	19.498901
O	-0.072606	6.247772	18.424830
O	5.018199	4.328162	31.196054
H	5.144506	3.645256	30.528449
H	5.655775	5.023192	31.000551
O	0.986127	5.615187	31.795422
H	0.323008	4.920255	31.867884
H	0.820301	6.231233	32.516889
N	2.985470	5.811274	29.590277
C	0.793176	2.012501	29.269173
C	1.322496	0.801648	29.729187
H	0.964691	-0.131859	29.311830
C	2.314895	0.797773	30.689156
C	2.783159	2.003169	31.179076
N	2.305960	3.172453	30.743409
C	1.345849	3.170525	29.814989
H	0.999380	4.142242	29.493150
C	-0.253928	2.063933	28.257513
C	-1.177386	1.109143	28.067082
H	-1.279515	0.213923	28.670830
C	-2.103658	1.439694	26.938873
H	-3.057570	1.828567	27.319778
H	-2.347094	0.569940	26.325158
C	-1.282898	2.495147	26.202306
H	-0.503933	1.925298	25.674094
C	-0.530692	3.237596	27.322036
C	-1.426994	4.247108	28.053415
H	-1.786979	5.032869	27.390458
H	-0.891990	4.739998	28.870629
H	-2.299352	3.754870	28.486324
C	0.653291	3.926571	26.656819
H	1.308639	3.162672	26.224454
H	1.259120	4.482157	27.382370
C	0.169114	4.883369	25.566186
H	-0.312268	5.743069	26.040772
H	1.038709	5.277828	25.033389
C	-0.789605	4.261975	24.540749
H	-0.195366	3.560119	23.937177
C	-1.901788	3.408780	25.169852
H	-2.646529	4.059730	25.640989
C	-2.596645	2.611938	24.077856
H	-1.976953	1.745705	23.802638
H	-3.534264	2.191337	24.456775
C	-2.872917	3.429744	22.863844
H	-3.557946	3.002416	22.136502
C	-2.355203	4.630449	22.618442
C	-1.354333	5.302387	23.544977

C	-2.052048	6.462006	24.267879
H	-2.590620	7.095976	23.564265
H	-1.335963	7.103780	24.787474
H	-2.784621	6.097814	24.991011
C	-0.190798	5.850180	22.694423
H	0.466718	6.456364	23.324767
H	0.406487	5.002071	22.338716
C	-0.621489	6.673951	21.492735
H	-1.153506	7.578763	21.804285
H	0.255793	6.988917	20.925168
C	-1.531723	5.852476	20.606565
H	-0.977224	4.993390	20.216913
C	-2.746995	5.388207	21.382330
H	-3.336361	6.275071	21.644992
H	-3.381849	4.764997	20.750139
C	-1.170493	6.734889	18.448921
C	-1.797673	7.528410	17.348096
H	-2.690231	7.013787	16.986926
H	-1.086514	7.649979	16.535442
H	-2.114804	8.503233	17.722171
C	2.985744	6.264923	28.537694
C	2.979157	6.836953	27.214016
H	1.950346	6.924079	26.858861
H	3.534142	6.190368	26.531763
H	3.443333	7.824658	27.234543
N	3.028448	4.166228	33.430011
C	3.049428	3.783114	34.509805
C	3.085151	3.304895	35.870006
H	3.408624	4.109541	36.533174
H	3.785454	2.471276	35.949238
H	2.091476	2.968086	36.171062
O	8.132585	10.785416	44.133065
O	6.287566	10.049621	45.177538
C	5.194712	7.936915	33.763072
C	4.583859	9.137180	33.390806
H	4.918708	10.071336	33.819606
C	3.559453	9.132911	32.460976
H	3.081921	10.055659	32.156821
C	3.148585	7.934394	31.913099
H	2.359740	7.890599	31.171988
N	3.700023	6.769627	32.275705
C	4.681744	6.778501	33.176598
H	5.073096	5.812090	33.470925
C	6.277817	7.848113	34.733815
C	7.229721	6.901305	34.734216
H	7.340878	6.130511	33.977933
C	8.184397	7.065968	35.876167
H	9.113371	7.545613	35.540518
H	8.473487	6.115352	36.330082
C	7.360027	7.959225	36.800141
H	6.607277	7.287868	37.242485
C	6.562299	8.860047	35.840503
C	7.424630	9.983436	35.246311

H	7.762550	10.681188	36.010861
H	6.870439	10.564773	34.505916
H	8.308766	9.581786	34.748592
C	5.390127	9.419893	36.636136
H	4.745721	8.589552	36.949154
H	4.767359	10.095855	36.041085
C	5.898401	10.177728	37.863579
H	6.373094	11.101865	37.527457
H	5.036827	10.488124	38.457979
C	6.870258	9.392141	38.751632
H	6.295540	8.577665	39.218739
C	7.987914	8.696692	37.960329
H	8.695983	9.445770	37.588692
C	8.739665	7.739569	38.870744
H	8.155127	6.814812	38.992892
H	9.679482	7.430768	38.399707
C	9.023041	8.326738	40.210015
H	9.742634	7.798462	40.830091
C	8.472430	9.440853	40.684795
C	7.426640	10.237420	39.922613
C	8.065049	11.542057	39.428240
H	8.595374	12.050284	40.232956
H	7.314440	12.239990	39.051503
H	8.794701	11.353069	38.637850
C	6.264343	10.559639	40.883759
H	5.565954	11.243195	40.394266
H	5.713167	9.632023	41.079970
C	6.693126	11.154183	42.214355
H	7.176994	12.126124	42.074035
H	5.818613	11.313793	42.847805
C	7.660018	10.220470	42.907363
H	7.152835	9.278696	43.137329
C	8.871162	9.966682	42.034031
H	9.416599	10.912378	41.932773
H	9.547806	9.263207	42.522728
C	7.339357	10.629753	45.209293
C	7.945059	11.269437	46.417683
H	8.943939	10.867067	46.593768
H	7.309894	11.091726	47.281382
H	8.052826	12.342875	46.249895
H	2.736213	-0.131284	31.051544
H	3.557177	2.042550	31.935979

9

CARTESIAN COORDINATES (ANGSTROEM)

Zn	-3.084133	6.305858	24.110139
O	-1.795750	4.516977	8.947580
N	-1.750263	5.326792	25.419358
O	-1.398938	2.328633	8.633761
O	-4.167256	4.578522	23.497044
O	3.688634	5.131796	37.827444

O	-4.077743	8.100257	23.436278
O	-4.805147	5.647899	25.240179
N	-4.991741	4.669286	24.470696
O	5.534959	6.401775	37.676816
O	-3.367011	10.033368	24.094947
N	-3.302422	8.835722	24.101937
N	-1.887688	6.229823	22.372700
O	-2.421752	8.214381	24.790159
O	-5.868730	3.866725	24.630028
C	-1.678693	3.412774	8.196549
C	-1.204951	5.979592	26.441569
H	-1.478752	7.023115	26.543164
C	-2.282230	5.536453	21.308567
H	-3.222293	5.005929	21.404502
C	-1.483993	4.030536	25.254828
H	-1.972250	3.543121	24.418545
C	-2.604494	3.469799	19.114071
H	-2.691176	2.922357	20.046148
C	-2.384543	3.863229	16.826380
H	-1.362405	3.456241	16.785686
C	0.025721	6.953346	21.173681
H	0.939738	7.533053	21.156957
C	-2.057330	4.684869	18.997553
C	-1.584074	4.369865	10.360125
H	-0.788559	3.634650	10.511096
C	-3.126835	2.956428	17.804358
H	-4.216927	3.083242	17.748648
H	-2.925490	1.894491	17.645871
C	2.400731	6.576969	34.609167
C	-1.119388	5.968102	16.889000
H	-0.182748	5.407472	16.992952
H	-0.966359	6.949765	17.348826
C	1.269487	6.778560	30.458988
H	2.235005	6.361147	30.134035
C	-1.440045	6.164886	15.406516
H	-0.578215	6.634047	14.925579
H	-2.254786	6.886663	15.324202
C	-0.756097	6.932532	22.312173
H	-0.494200	7.496759	23.200041
C	2.742426	7.631660	33.873928
H	3.315573	8.426311	34.345734
C	0.220065	5.797431	29.916510
C	-1.173027	5.717137	10.900290
H	-1.938077	6.455180	10.639055
H	-0.244961	6.040640	10.421551
C	-1.807714	4.883072	14.648995
H	-0.896475	4.268031	14.594216
C	-2.241159	5.197397	17.572589
C	-0.646534	3.347052	26.114671
H	-0.449825	2.294869	25.953433
C	-1.559366	5.486047	20.117701
C	0.235555	6.182772	28.440397
C	-3.335334	6.207176	13.094925

H	-4.161120	5.962702	13.765844
H	-3.747993	6.267958	12.087749
H	-2.964971	7.201831	13.351290
C	0.657373	4.403044	30.346483
H	1.623571	4.171243	29.882699
H	-0.055052	3.638802	30.019557
C	-1.203794	6.087981	30.411989
H	-1.320742	5.907237	31.480464
H	-1.917958	5.447748	29.887419
H	-1.484646	7.124022	30.214014
C	0.665211	7.442831	28.307727
H	0.684215	7.989571	27.371426
C	-2.833311	4.012446	15.390347
H	-3.817531	4.494440	15.357907
C	1.085896	8.039030	29.618381
H	0.297761	8.695485	30.012579
H	1.992157	8.644671	29.546066
C	1.647733	5.434145	32.499797
H	2.687858	5.244040	32.193890
C	4.750794	5.772593	38.335599
C	-0.378003	6.224400	20.068630
H	0.230549	6.213371	19.173910
C	-0.983846	5.640242	12.406483
H	-0.684092	6.622187	12.780667
H	-0.156051	4.954446	12.622448
C	-2.852715	3.883572	11.036400
H	-3.664903	4.556065	10.733614
H	-3.105207	2.884615	10.678058
C	2.388204	7.816656	32.439743
H	3.291155	7.701349	31.821797
H	2.052974	8.846236	32.273690
C	2.765030	6.518919	36.065394
H	1.863279	6.566842	36.688440
H	3.388483	7.375115	36.326739
C	-3.530149	6.031722	17.590401
H	-3.808018	6.396116	16.601415
H	-3.401048	6.901423	18.240143
H	-4.367886	5.451580	17.981611
C	0.220304	5.388138	34.612461
H	0.221333	5.528867	35.693473
H	-0.293423	4.445979	34.411031
H	-0.368646	6.201542	34.184130
C	1.325006	6.838600	31.968303
H	0.355927	7.167798	32.361759
C	-2.223894	5.152653	13.182485
C	4.817419	5.597562	39.822075
H	3.946224	6.067103	40.283600
H	5.727087	6.055169	40.202248
H	4.789250	4.537451	40.078337
C	1.646660	5.384127	34.047286
C	3.486791	5.228870	36.409091
H	4.467055	5.221758	35.924083
C	-2.711168	3.869644	12.531960

C	-2.940967	2.659907	14.706261
H	-2.082164	2.035524	14.994296
H	-3.824116	2.123124	15.069490
C	-0.067777	4.027851	27.171728
H	0.606891	3.513793	27.843602
C	-2.997726	2.769355	13.222574
H	-3.303513	1.880838	12.675189
C	-1.945136	3.718998	6.754346
H	-2.980431	4.045563	6.636272
H	-1.768576	2.829855	6.154610
H	-1.306007	4.537018	6.418645
C	-0.338251	5.382847	27.356268
C	0.785537	4.333215	31.869422
H	-0.217196	4.361433	32.299726
H	1.195309	3.356251	32.137420
C	2.378356	4.102348	34.494035
H	1.789966	3.228567	34.203491
H	3.325066	4.041834	33.944200
C	2.673466	4.031614	35.983504
H	1.746562	4.004679	36.565163
H	3.218467	3.112533	36.214653

10

CARTESIAN COORDINATES (ANGSTROEM)

O	5.495803	5.342008	23.256117
C	6.084244	2.937691	19.241884
H	6.761263	2.324248	19.830793
C	4.333324	2.837672	15.857627
H	3.532546	2.222113	16.297089
O	3.606340	4.753516	24.314530
C	5.648286	4.081626	19.762775
C	6.117357	4.522756	21.119627
H	6.730230	3.742021	21.574010
H	6.750764	5.413805	21.036245
C	5.726040	2.425092	17.889746
H	5.062132	1.553062	17.993595
H	6.625776	2.048648	17.390456
C	5.049044	3.476629	17.025476
H	5.817443	4.167809	16.661536
C	4.016314	4.245408	17.863067
H	3.379834	3.470878	18.318262
C	3.561699	5.401587	20.037839
H	2.927033	6.166182	19.582778
H	2.924424	4.526710	20.214621
C	3.599274	3.837542	14.946294
C	3.200124	2.892923	13.816248
C	4.671102	4.998173	19.045575
C	4.950801	4.864209	22.023292
H	4.358731	3.968748	22.234383
C	4.067354	5.908093	21.377856
H	4.640190	6.833352	21.258807

H	3.222560	6.129726	22.032653
C	4.709193	5.229819	24.342339
C	4.545993	4.898975	14.367260
H	4.961839	5.538065	15.144665
H	4.029099	5.551107	13.659686
H	5.379073	4.437200	13.834799
C	5.392454	5.773334	25.556501
H	6.358039	5.284448	25.694854
H	4.762955	5.619088	26.428827
H	5.585223	6.839609	25.421924
C	4.061888	1.865303	13.763233
H	4.084945	1.112505	12.981446
C	5.053323	1.905577	14.885802
H	6.014318	2.311214	14.542799
H	5.265313	0.918322	15.302748
C	2.499257	4.472079	15.787073
H	1.791997	3.691026	16.091010
H	1.924685	5.217636	15.228038
C	5.424102	6.249562	18.576493
H	6.005482	6.685635	19.388394
H	4.739058	7.023581	18.224262
H	6.126891	6.013962	17.774425
C	3.099241	5.142563	17.023510
H	2.281768	5.507748	17.648371
H	3.645848	6.032114	16.704018
N	-1.151657	1.366080	8.552375
C	-1.291758	1.885010	7.538229
C	-0.011144	3.349916	11.123720
H	-0.841747	3.400366	10.430466
N	0.449302	2.131612	11.426979
C	1.463372	2.021483	12.285087
H	1.779354	1.014762	12.533727
C	2.100632	3.109047	12.883159
C	1.592694	4.370964	12.562891
H	2.031420	5.257738	12.998741
C	0.535420	4.489551	11.678690
H	0.134131	5.461243	11.420622
C	-1.469870	2.535012	6.264053
H	-1.956145	1.846477	5.569189
H	-0.498165	2.826989	5.861446
H	-2.090610	3.424532	6.385925
Cd	-0.533149	0.239171	10.587958
O	-4.808806	1.197296	-1.845799
C	-5.563011	-2.105667	1.439123
H	-6.126706	-2.607227	0.657052
C	-4.155743	-2.870524	4.901298
H	-3.272429	-3.338087	4.441428
O	-2.759721	1.075885	-2.751263
C	-5.175332	-0.849461	1.235078
C	-5.548741	-0.135227	-0.032338
H	-6.044455	-0.826758	-0.715818
H	-6.264175	0.669775	0.174844
C	-5.294131	-2.893746	2.674269

H	-4.554340	-3.677467	2.454304
H	-6.203042	-3.426490	2.973548
C	-4.792967	-2.029070	3.819473
H	-5.646665	-1.476937	4.228313
C	-3.744766	-1.042389	3.284107
H	-3.019094	-1.659984	2.734930
C	-3.188551	0.621489	1.492033
H	-2.662219	1.294804	2.175327
H	-2.467090	-0.146118	1.187945
C	-3.594632	-2.054211	6.080683
C	-3.262525	-3.194618	7.039849
C	-4.345110	-0.069399	2.241960
C	-4.338221	0.482171	-0.701579
H	-3.651522	-0.298610	-1.042493
C	-3.613288	1.399597	0.258429
H	-4.276652	2.229424	0.523778
H	-2.736446	1.824872	-0.232693
C	-3.907458	1.423605	-2.821239
C	-4.665028	-1.170066	6.737491
H	-5.048105	-0.410159	6.057091
H	-4.266890	-0.651402	7.614621
H	-5.512535	-1.768118	7.075720
C	-4.532900	2.142636	-3.973048
H	-5.315356	1.520707	-4.412369
H	-3.774747	2.364437	-4.719340
H	-5.006193	3.063583	-3.628750
C	-4.048663	-4.248545	6.771947
H	-4.101382	-5.152282	7.368961
C	-4.904795	-4.022052	5.565757
H	-5.927902	-3.752029	5.860522
H	-4.988694	-4.909406	4.934901
O	-2.766191	0.868136	11.216026
H	-3.471951	1.155264	10.630030
H	-3.174588	0.655227	12.059995
C	-2.453120	-1.218183	5.515409
H	-1.676849	-1.890520	5.135080
H	-1.974850	-0.598807	6.283650
C	-5.236751	0.992145	2.899440
H	-5.786119	1.562182	2.150901
H	-4.649984	1.712407	3.476032
H	-5.979043	0.537955	3.559020
C	-2.963358	-0.318891	4.388289
H	-2.109673	0.190818	3.932135
H	-3.597263	0.461825	4.819944
C	-0.110111	-1.426830	15.282001
H	0.254236	-0.584765	15.873479
H	0.604019	-2.249527	15.349882
H	-1.072788	-1.755212	15.677971
N	-0.373442	-0.699199	12.810438
C	-0.259084	-1.023176	13.905123
N	-1.076248	-1.817634	9.739737
C	-0.546031	-2.930589	10.254181
H	0.139592	-2.809152	11.084626

C	-0.853093	-4.180917	9.750178
H	-0.395226	-5.063466	10.178397
C	-1.732962	-4.279864	8.690509
H	-1.959904	-5.248619	8.261981
C	-2.318776	-3.130336	8.149036
C	-1.933154	-1.918898	8.721359
H	-2.335563	-0.988459	8.346928
O	1.724916	-0.324420	9.875129
H	2.431369	0.319024	9.763947
H	1.931726	-1.066403	9.298678

12

CARTESIAN COORDINATES (ANGSTROEM)

Fe	2.388275	2.894991	9.837406
N	1.918231	5.169217	12.252288
O	-3.552787	3.259127	21.412270
N	0.963484	3.283169	11.105873
N	1.343894	1.429461	9.105084
N	-0.619863	1.587056	10.483257
N	3.812156	2.506807	8.567864
N	1.525354	4.175858	8.512976
N	3.252167	1.609458	11.163435
N	5.403758	4.190816	9.201863
C	0.103382	1.052799	9.523940
N	3.433799	4.358810	10.569512
C	-0.303159	4.300519	12.736376
O	-3.065867	1.707818	22.962149
C	-0.219820	2.616134	11.198510
C	3.056969	5.181931	11.587803
C	3.858936	1.072995	13.431359
C	3.815367	1.496694	7.653108
C	-0.358041	-0.072904	8.727864
C	3.522188	1.982054	-0.247193
H	3.245345	1.530736	-1.197634
C	4.388793	-0.428315	11.642635
H	4.804281	-1.354019	11.265547
C	4.421096	-0.122923	12.988288
H	4.846677	-0.814430	13.705883
C	-2.081598	4.835282	14.212486
H	-2.513211	5.456395	14.989216
C	-1.043272	3.228407	12.228712
C	1.469215	3.870095	7.217874
H	1.895666	2.922232	6.927796
C	0.995985	5.340723	8.901017
H	1.066900	5.583603	9.950490
C	-2.817394	3.754882	13.714901
H	-3.804795	3.558006	14.116561
N	2.853025	0.628392	7.412180
C	-1.527943	-0.815337	8.744390
H	-2.313772	-0.592395	9.455749
C	4.594349	3.481553	3.671573

H	4.978308	4.477716	3.444212
H	5.421245	2.940107	4.134277
C	1.923757	2.380240	17.941439
H	2.207069	3.437769	17.989218
C	0.957373	2.216016	20.243965
H	1.097902	2.034956	21.307178
C	3.472290	3.610787	4.698211
H	3.200058	2.615268	5.063238
H	3.831538	4.181265	5.560769
C	0.662053	-0.351257	7.815227
C	2.254869	4.256830	4.058860
C	4.308832	1.499972	17.154678
H	4.792930	2.438765	17.456391
H	4.564495	0.756225	17.913808
C	3.295095	1.900959	12.463385
H	2.858635	2.844295	12.748505
C	0.946526	4.297753	4.837448
C	-0.168656	2.803666	19.846897
C	0.462781	2.256577	17.477934
H	0.204906	1.196878	17.627882
C	5.000142	3.167005	8.482153
C	4.728531	1.088611	15.770955
H	5.677535	0.610664	15.554188
C	-0.498592	3.057989	18.386780
C	1.834405	3.371457	2.880926
H	1.778359	2.357732	3.305907
C	2.803715	1.657026	16.946814
H	2.418960	0.627069	16.895042
C	5.825296	2.551222	7.454815
C	2.059577	1.791755	19.335798
H	2.078017	0.693718	19.269777
H	3.024114	2.072724	19.773015
C	2.866400	3.303742	1.777100
H	3.030721	4.303140	1.356408
C	-2.570903	2.704463	20.521481
H	-2.576134	1.617720	20.643646
C	-0.628788	-2.121747	6.908752
H	-0.758196	-2.933123	6.201767
C	-2.967377	3.084241	19.116212
H	-3.059489	4.173314	19.054270
H	-3.952099	2.669484	18.884574
C	6.443811	7.604285	11.882944
H	7.351254	8.196517	11.908962
C	5.142722	5.849208	10.959906
C	-1.937272	2.569011	18.124343
H	-2.235638	2.854131	17.112587
H	-1.936073	1.473080	18.155003
C	-0.813424	5.120465	13.731233
H	-0.232953	5.951525	14.113802
C	2.679473	2.192524	15.510968
C	4.117655	6.134441	11.865166
C	1.259324	1.897815	15.052610
H	1.110040	0.812023	15.044594

H	1.072541	2.246463	14.034389
C	-1.650066	-1.843605	7.822392
H	-2.551837	-2.444802	7.806369
C	5.418192	7.888079	12.790099
H	5.550210	8.694498	13.502238
C	4.240678	7.156381	12.793122
H	3.440932	7.367726	13.492599
C	4.678605	4.728161	10.159019
C	7.098568	2.828002	6.981417
H	7.669129	3.656354	7.383340
C	6.426883	1.796886	1.861368
H	6.868341	2.235299	2.758765
H	5.956962	0.856078	2.173077
C	3.808246	1.409492	14.852736
C	4.814307	2.155160	0.018773
C	7.526930	1.497547	0.855734
H	8.067554	2.405896	0.588757
H	8.250687	0.801482	1.290166
C	-1.191570	3.242235	20.855879
H	-1.263003	4.336877	20.877764
H	-0.895571	2.913794	21.853266
C	0.541617	-1.378786	6.893261
H	1.338262	-1.586632	6.189266
C	5.589751	0.664574	5.947141
H	5.003742	-0.158413	5.556105
C	0.380743	3.762594	2.616406
H	0.295563	4.642877	1.964258
H	-0.199965	2.965015	2.146883
C	0.892510	4.687459	6.250495
C	1.717951	0.609919	8.083327
C	2.409447	2.382613	0.657998
H	1.947504	1.484074	1.093416
H	1.616011	2.862934	0.074767
C	-3.697080	2.666498	22.604972
C	-2.308711	2.938970	12.715681
H	-2.874475	2.104145	12.320390
C	-0.080765	4.082721	4.013617
H	-1.124339	4.137875	4.302902
C	3.786995	0.452767	10.764253
H	3.725754	0.221944	9.711682
C	-0.413115	4.565293	18.109727
H	-0.958467	5.139582	18.859239
H	-0.838704	4.813520	17.135388
H	0.622515	4.910158	18.131302
C	2.557864	5.707954	3.660754
H	2.719716	6.310881	4.558553
H	1.723148	6.146824	3.110443
H	3.450594	5.794595	3.041655
C	5.079830	1.487461	6.939387
C	0.258597	2.564122	15.990695
H	0.305937	3.640802	15.825744
H	-0.748910	2.269314	15.689889
C	4.181036	2.774173	2.374831

H	3.972859	1.725668	2.638684
C	7.610194	2.005692	5.988873
H	8.604032	2.191680	5.598146
C	0.957259	4.297977	12.016229
C	0.326210	5.882777	6.677062
H	-0.131708	6.549097	5.955397
C	-4.746151	3.360143	23.420191
H	-4.446230	4.394022	23.601657
H	-4.875779	2.839981	24.365692
H	-5.688895	3.386895	22.871334
C	5.857554	1.782415	-0.996840
H	5.385881	1.276463	-1.842630
H	6.349172	2.678478	-1.382511
C	6.867402	0.934479	5.481891
H	7.299291	0.310509	4.708116
C	0.380930	6.208618	8.020811
H	-0.038234	7.134476	8.393388
C	3.003213	3.691243	15.423995
H	2.301142	4.293738	16.000357
H	2.956446	4.055964	14.397448
H	4.007853	3.894042	15.799698
C	5.320947	2.729878	1.329599
C	6.915395	0.880457	-0.383516
H	6.439341	-0.063305	-0.100283
C	5.907171	4.123619	1.067103
H	6.591590	4.116244	0.218551
H	6.467513	4.486614	1.930846
H	5.117128	4.842898	0.841332
C	6.319541	6.580981	10.955751
H	7.109549	6.352953	10.250662
O	8.703834	2.519198	-1.730512
C	9.637375	0.638263	-2.885722
H	9.060437	0.108315	-3.645625
H	10.229719	-0.105709	-2.350131
H	10.290793	1.369382	-3.354694
C	8.709745	1.333217	-1.933360
O	7.896986	0.448041	-1.347271

13

CARTESIAN COORDINATES (ANGSTROEM)

Zn	4.427125	7.533637	3.498325
N	5.921263	6.278064	4.192286
N	5.027807	7.118296	1.565763
N	3.539479	7.456374	5.375352
C	0.073474	8.921058	5.428337
N	2.657753	8.303740	2.759062
C	1.571690	8.703082	3.474436
N	5.574796	9.369373	3.727560
C	5.339188	6.277173	6.579074
C	0.020401	9.873049	6.444907
H	0.943266	10.318674	6.792654

C	6.174913	5.982253	5.495494
C	2.606121	11.420689	11.141090
C	-2.334313	8.738622	5.541977
H	-3.252847	8.283662	5.189150
C	-1.122903	8.358186	4.985895
H	-1.093939	7.606236	4.205766
C	8.324508	5.311627	1.460113
C	4.492804	7.678467	0.447076
C	2.449812	8.674253	1.466741
C	-1.189108	10.255287	7.002547
H	-1.205829	11.001220	7.789444
C	2.288631	7.882805	5.709593
C	2.719357	12.624096	12.060880
C	6.199238	6.528421	1.203555
C	5.526016	12.187841	6.166190
C	4.086985	6.899880	6.490611
C	5.961109	11.314023	5.079235
C	5.364965	7.437431	-0.674274
H	5.198611	7.783945	-1.681830
C	2.030599	7.573379	7.092178
H	1.105534	7.761129	7.613116
C	3.145921	6.975140	7.577274
H	3.305976	6.583323	8.569178
C	3.593253	10.343690	9.035005
H	3.742055	9.381565	9.530183
H	2.577473	10.306768	8.636967
C	-2.372542	9.688980	6.552031
H	-3.320109	9.985157	6.986853
C	0.642303	9.374665	2.601336
H	-0.298266	9.807659	2.902028
C	7.910523	5.156454	4.333539
H	8.819229	4.667627	4.020468
C	1.371123	8.497652	4.846578
C	5.782709	5.870490	7.935372
C	7.434707	5.289151	5.594952
H	7.890740	4.946214	6.509813
C	3.100069	12.088225	6.618358
H	3.041412	13.105286	6.226313
H	2.305744	11.971408	7.355063
H	2.882551	11.409095	5.792197
C	3.715098	11.472725	10.064092
H	4.663285	11.315477	10.600760
C	2.122171	9.925388	-3.440345
H	1.823909	10.310103	-4.408737
C	3.839796	12.873828	9.444206
H	2.867203	13.191846	9.051831
C	7.102990	5.896902	2.068586
C	4.486834	11.812658	7.217371
C	6.959170	5.794283	3.458321
C	6.417819	6.722307	-0.207824
H	7.275158	6.373837	-0.761512
C	3.478509	13.681036	11.782355
H	3.536703	14.482319	12.515365

C	5.207282	10.209779	4.686514
H	4.266450	9.973593	5.163169
C	7.161444	11.516387	4.394683
H	7.809475	12.337414	4.678981
C	5.268611	14.075677	7.560152
H	4.411314	14.706184	7.285686
H	5.957107	14.711768	8.122452
C	2.890757	8.933048	-0.942976
C	2.493902	10.797277	-2.427447
H	2.491817	11.867079	-2.602942
C	1.932147	12.571765	13.339040
H	0.856037	12.606448	13.127832
H	2.171975	13.436241	13.959812
C	6.711722	9.584216	3.074012
H	6.966365	8.867606	2.301268
C	3.299404	8.408735	0.384966
C	7.532154	10.657894	3.377804
H	8.458465	10.801247	2.835783
C	4.849786	12.820569	8.320428
H	5.780068	12.430823	8.760522
C	9.581910	5.852120	1.721557
H	9.659979	6.709602	2.380614
C	10.619738	4.214903	0.300260
H	11.509490	3.789486	-0.149245
C	1.182869	9.351945	1.359117
H	0.759247	9.744631	0.448517
C	5.897223	13.463480	6.342438
H	6.539901	14.019777	5.669295
C	1.948789	10.082890	13.240552
H	0.885232	10.044847	12.984499
H	2.171675	9.171084	13.800962
C	8.235398	4.213099	0.607906
H	7.261158	3.783872	0.403450
C	2.874018	10.304299	-1.188757
H	3.168486	10.984195	-0.397286
C	4.253408	13.860739	10.522628
H	5.327650	13.747674	10.731344
H	4.133529	14.888198	10.161628
C	4.559598	10.431673	7.857480
H	5.588792	10.264435	8.196408
H	4.324175	9.624008	7.162178
C	2.202533	11.291120	14.107660
H	3.234630	11.296821	14.469425
C	2.807460	10.147926	11.987953
H	2.612367	9.267619	11.370888
H	3.862724	10.098177	12.281824
C	10.720591	5.309372	1.146602
H	11.690207	5.746004	1.357539
C	9.373177	3.668171	0.033103
H	9.285375	2.809691	-0.623014
C	2.515498	8.066609	-1.967325
H	2.518424	6.998654	-1.781075
C	1.206224	11.402917	10.510841

H	0.429069	11.539292	11.263348
H	1.004695	10.455120	10.007386
H	1.094404	12.210699	9.784660
C	2.133513	8.558170	-3.206049
H	1.838641	7.869766	-3.989875
C	6.583616	5.138915	10.512642
H	6.893489	4.854317	11.511492
C	6.428826	6.477944	10.184372
H	6.622108	7.245930	10.924941
C	6.337269	4.166027	9.554804
H	6.447468	3.117049	9.805217
C	5.940215	4.528971	8.276935
H	5.735112	3.767952	7.532755
C	6.030543	6.838980	8.906974
H	5.912273	7.884573	8.646846
O	1.330375	11.197810	15.245154
C	1.703980	11.851359	16.353695
O	2.716642	12.492152	16.448479
C	0.691765	11.672147	17.443942
H	-0.260459	12.107400	17.134057
H	1.042878	12.158263	18.350503
H	0.519777	10.610061	17.625493

5. NMR studies

5.1. General experimental details

^1H and ^{13}C NMR spectra were recorded on a 300 MHz (300.1, 75.5 MHz, respectively) and 400 MHz (400.1, 100.6 MHz, respectively) spectrometers in $CDCl_3$, CD_2Cl_2, TCE-d_2 (1,1,2,2-tetrachloroethane), DMSO-d_6, and methanol-d_4 solutions using 0.05% Me_4Si as the external or internal standard. Determinations/studied of structures and stereochemistry of obtained compounds and assignments of ^1H, ^{13}C signals were made with the aid of 2D COSY, TOCSY, NOESY, ROESY, edited-HSQC, HMBC spectra, as well as using diffusion-ordered NMR studies (DOSY-LED).

5.2. Comparison of key NMR spectra for structural studies

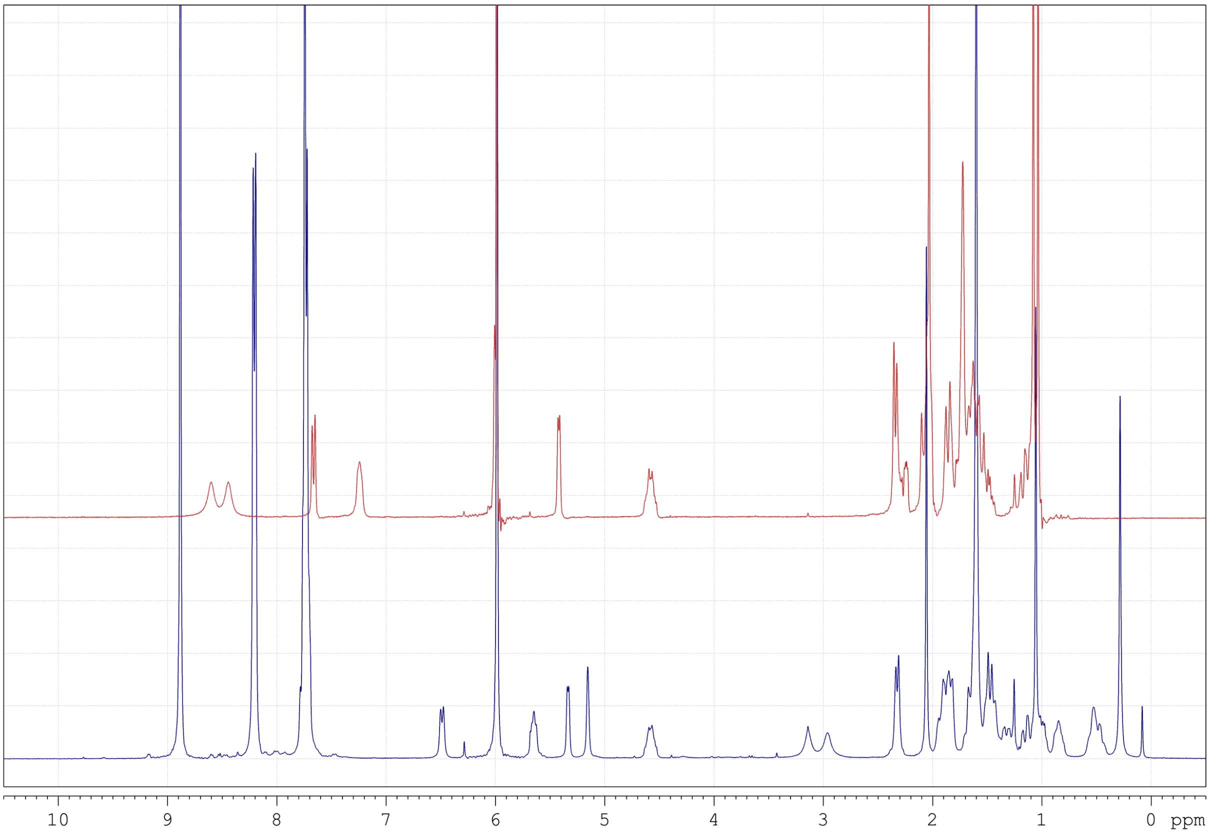

Figure S10. Comparison of ^1H spectra in 1,2-TCE-d_2 (blue – complex, red –abiraterone)

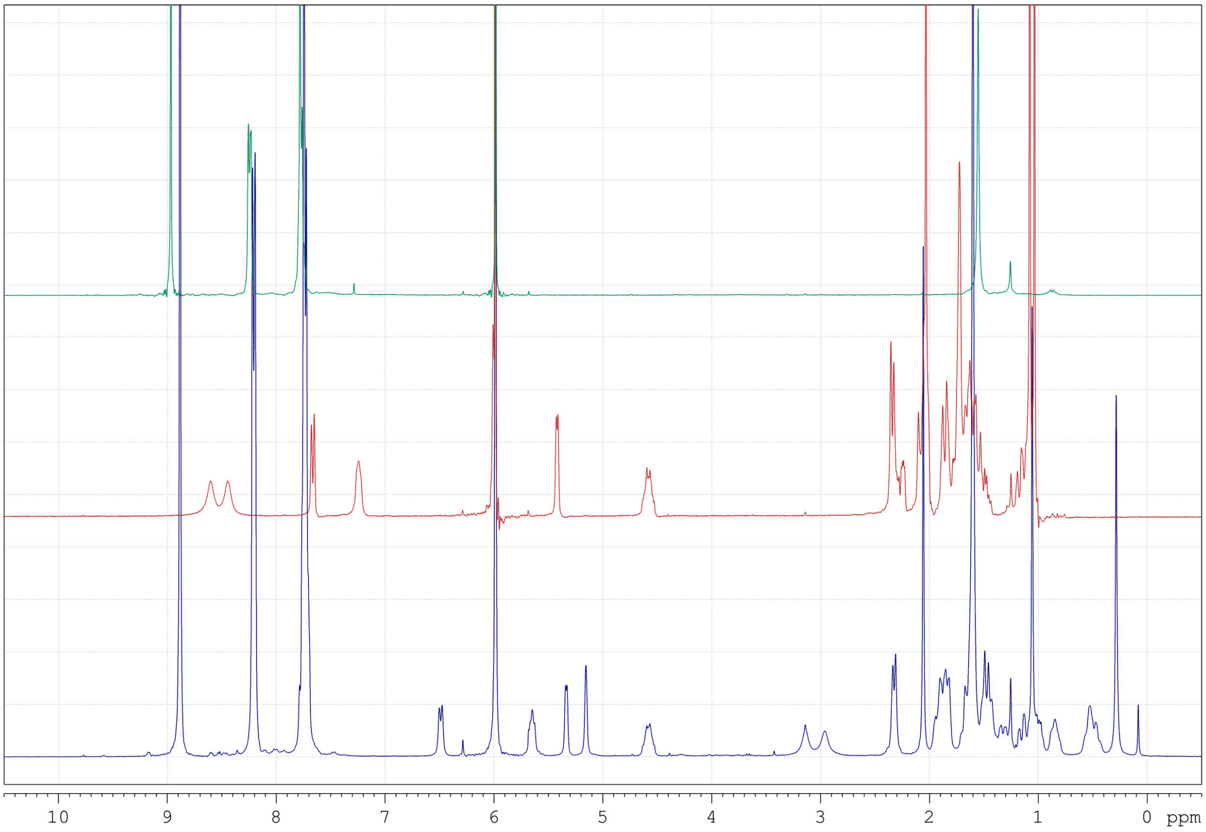

Figure S11. Comparison of ^{1}H spectra in 1,2-TCE-d_2 (blue – complex, red –abiraterone, green – TPPZn)

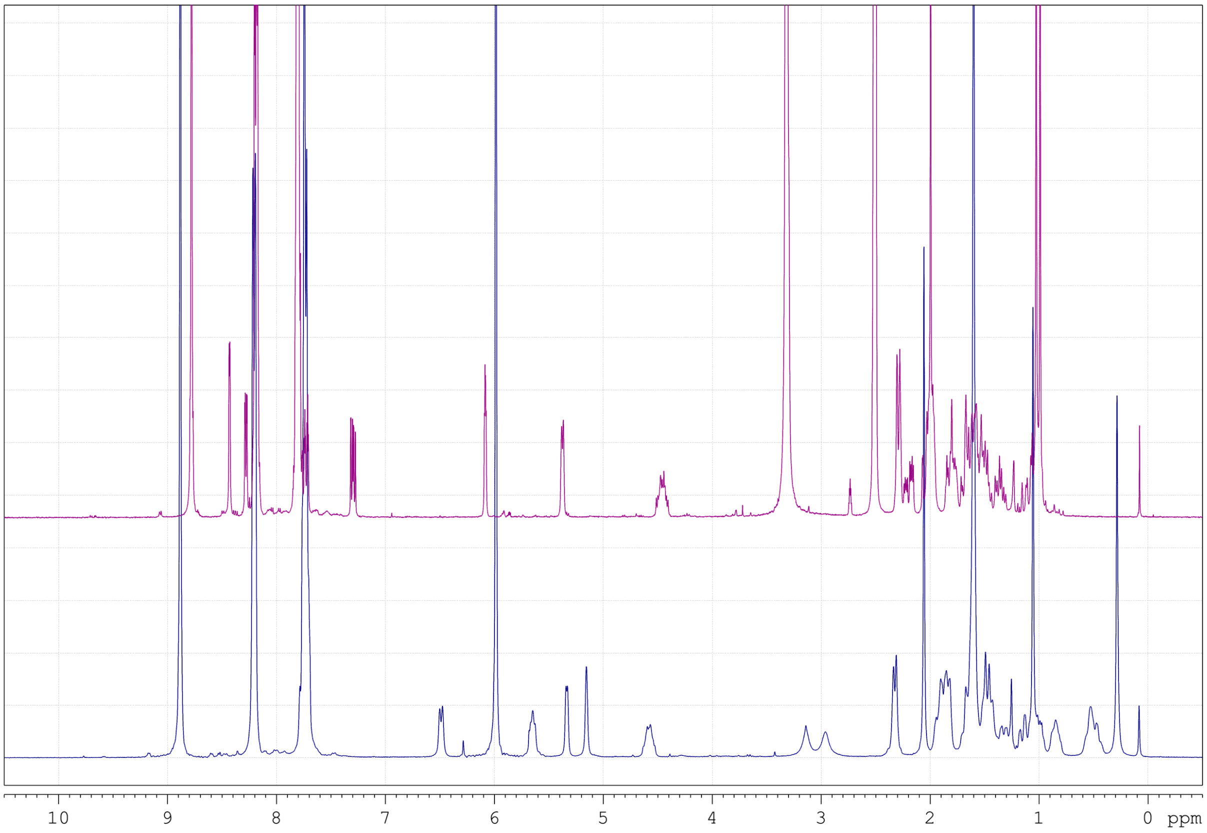

Figure S12. Comparison of ^{1}H spectra of complex (blue – in 1,2-TCE-d_2, violet – in DMSO-d_6)

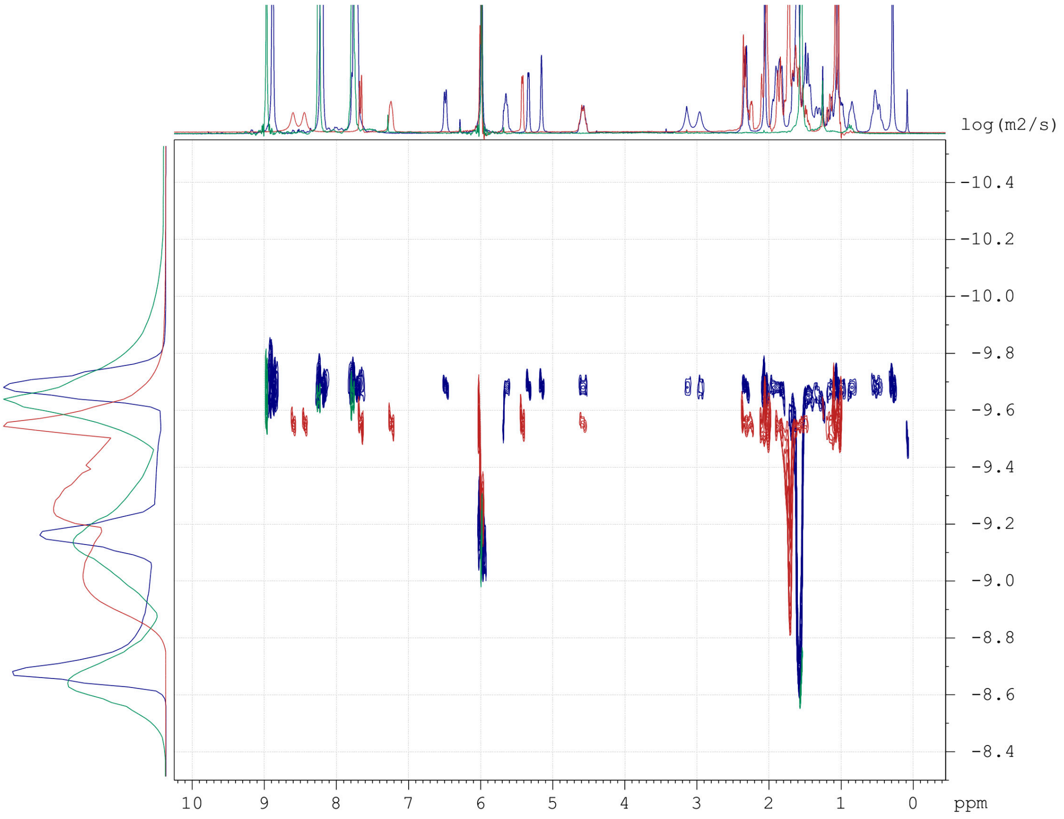

Figure S13. Comparison of 2D ^{1}H DOSY spectra in 1,2-TCE-d_2 (blue – complex, red –abiraterone, green – TPPZn)

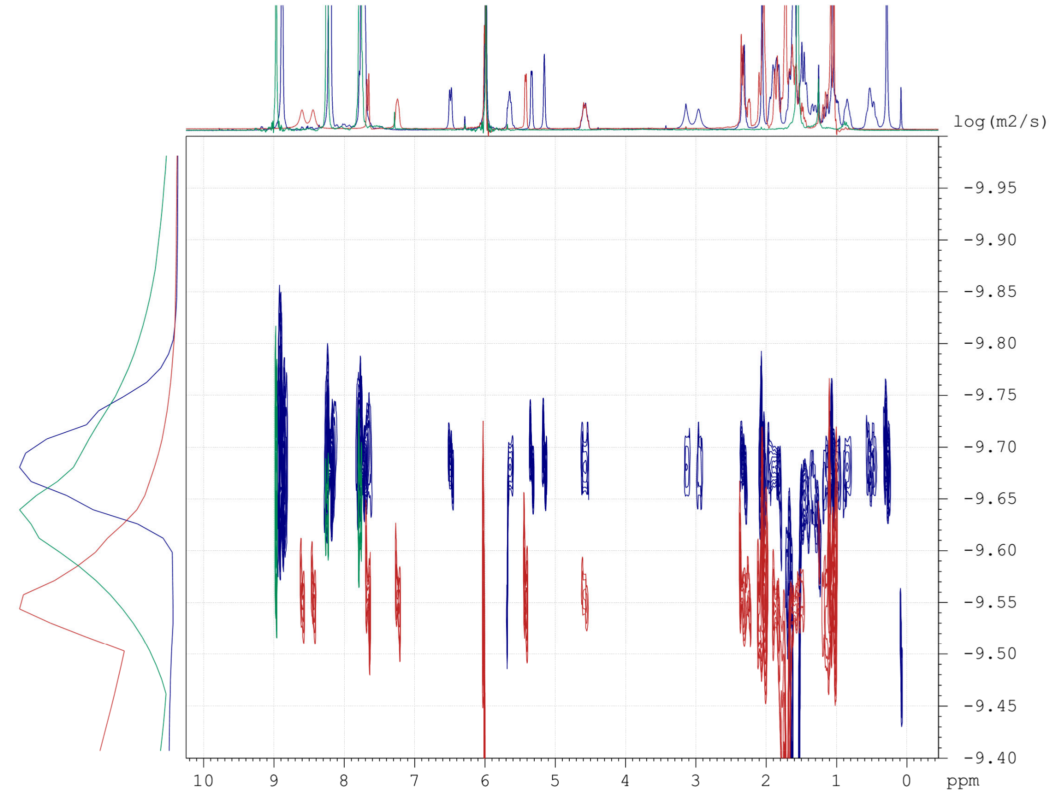

Figure S14. Comparison of 2D ^{1}H DOSY spectra in 1,2-TCE-d_2 (blue – complex, red –abiraterone, green – TPPZn)

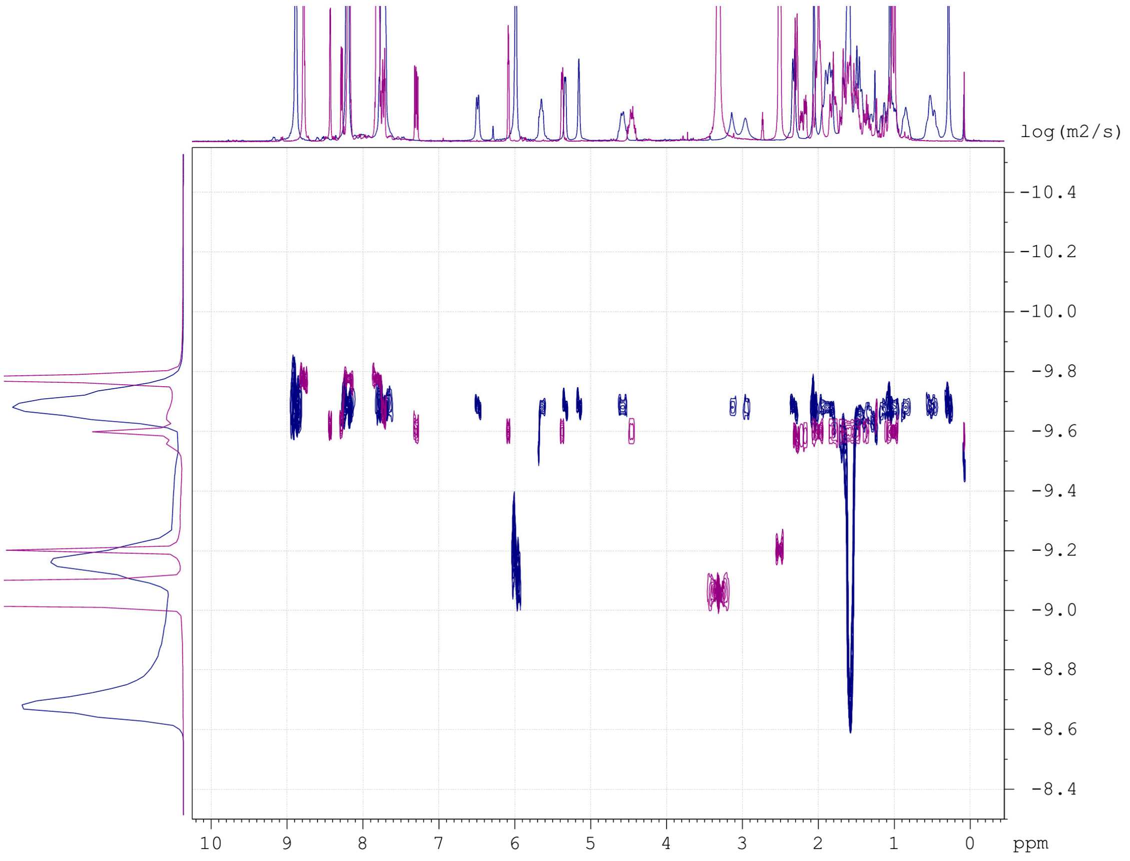

Figure S15. Comparison of 2D ^{1}H DOSY spectra of complex (blue – in 1,2-TCE-d_2, violet – in DMSO-d_6)

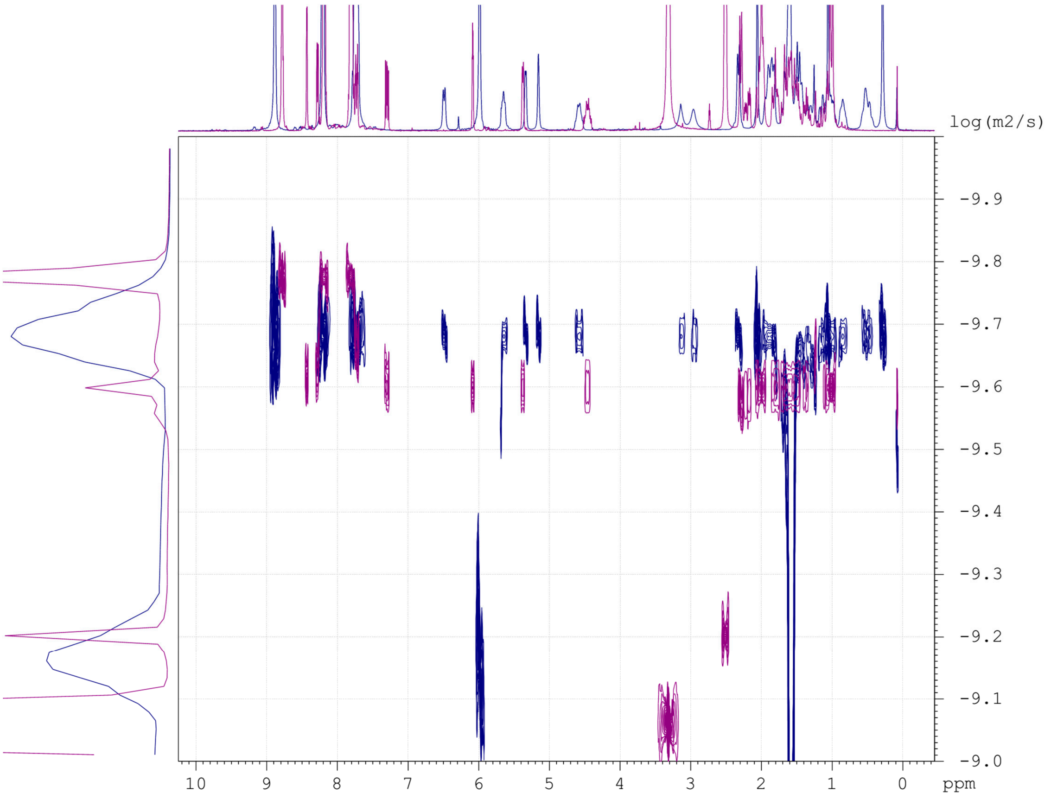

Figure S16. Comparison of 2D ^{1}H DOSY spectra of complex (blue – in 1,2-TCE-d_2, violet – in DMSO-d_6)

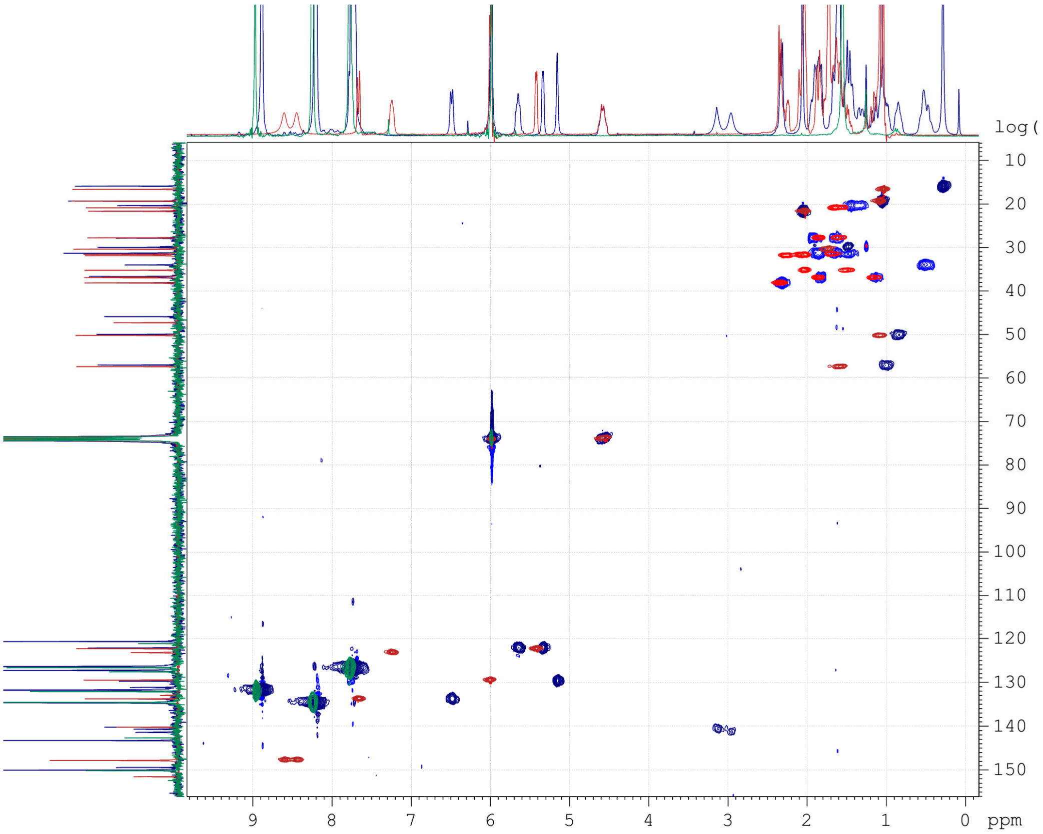

Figure S17. Comparison of 2D ^{1}H–^{13}C edited-HSQC spectra in 1,2-TCE-d_2 (blue – complex, red – abiraterone, green – TPPZn)

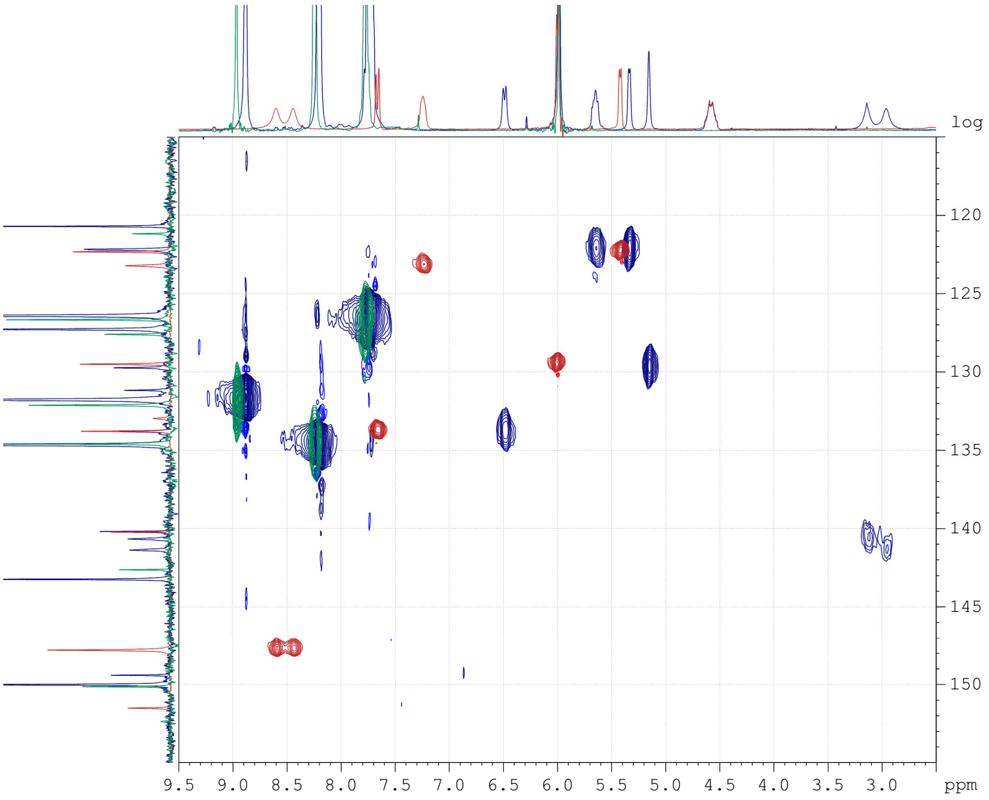

Figure S18. Comparison of 2D ^{1}H–^{13}C edited-HSQC spectra in 1,2-TCE-d_2 (blue – complex, red – abiraterone, green – TPPZn)

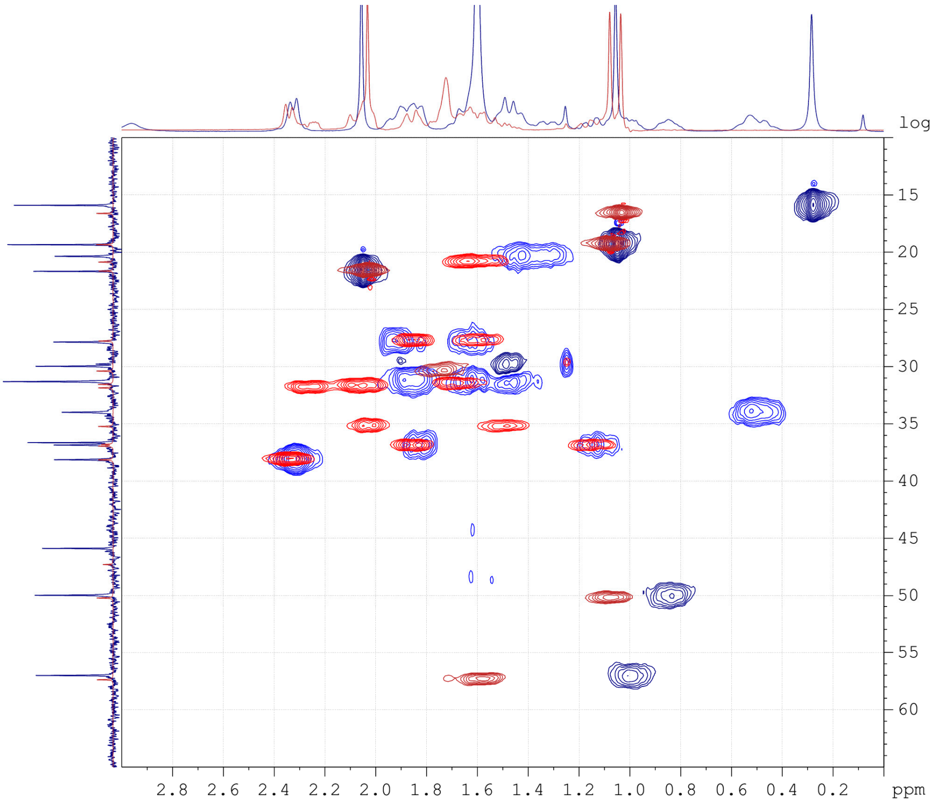

Figure S19. Comparison of 2D ^{1}H–^{13}C edited-HSQC spectra in 1,2-TCE-d_2 (blue – complex, red – abiraterone, green – TPPZn)

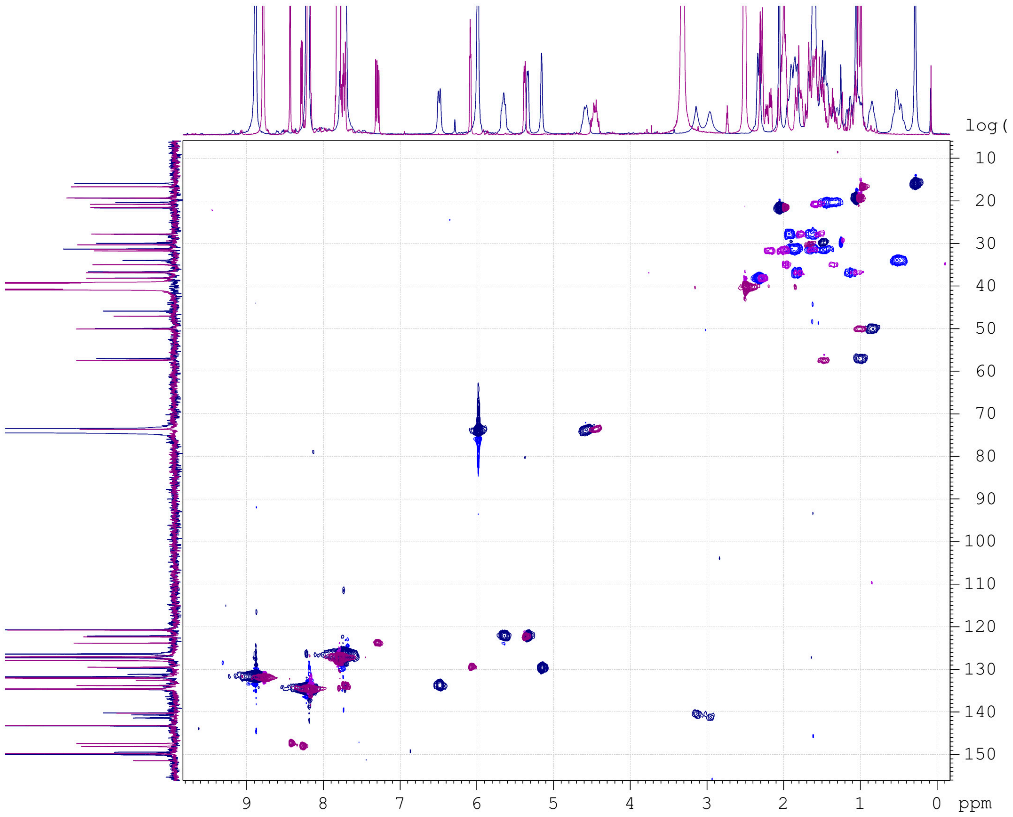

Figure S20. Comparison of 2D ^{1}H–^{13}C edited-HSQC spectra of complex (blue – in 1,2-TCE-d_2, violet – in DMSO-d_6)

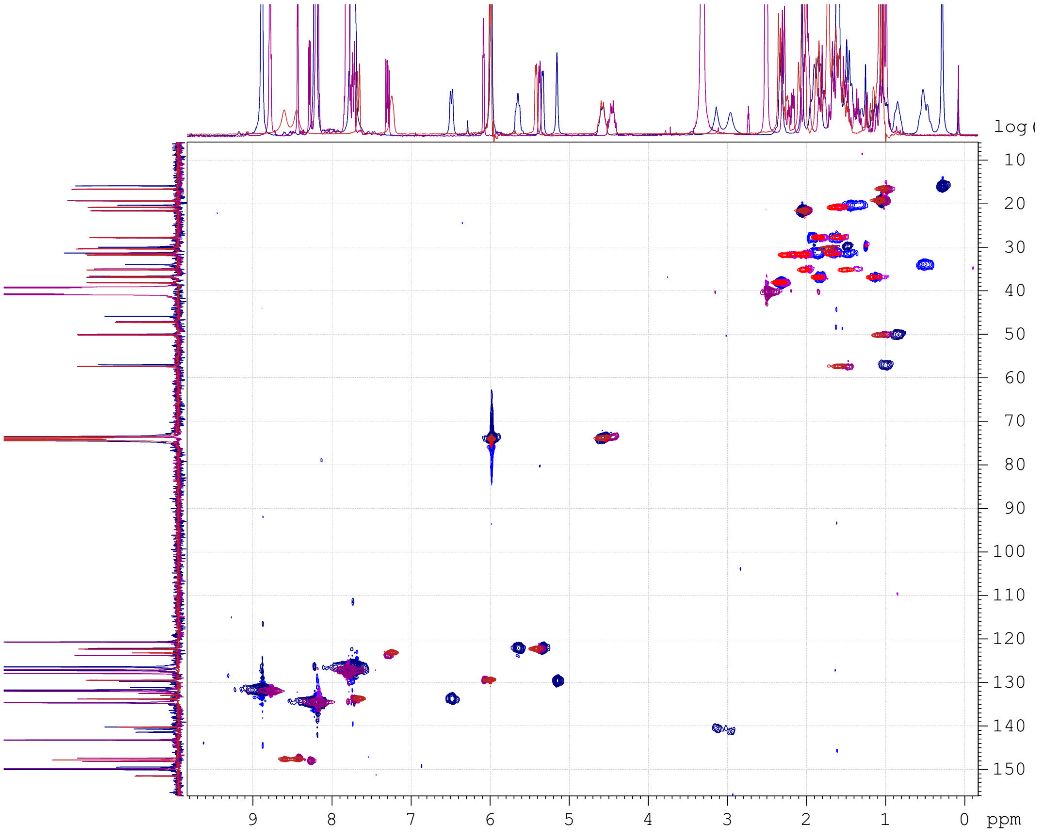

Figure S21. Comparison of 2D ^{1}H–^{13}C edited-HSQC spectra of complex (blue – in 1,2-TCE-d_2, violet – in DMSO-d_6) and abiraterone in 1,2-TCE-d_2 (red)

5.3. NMR data for complex in 1,2-TCE-d_2

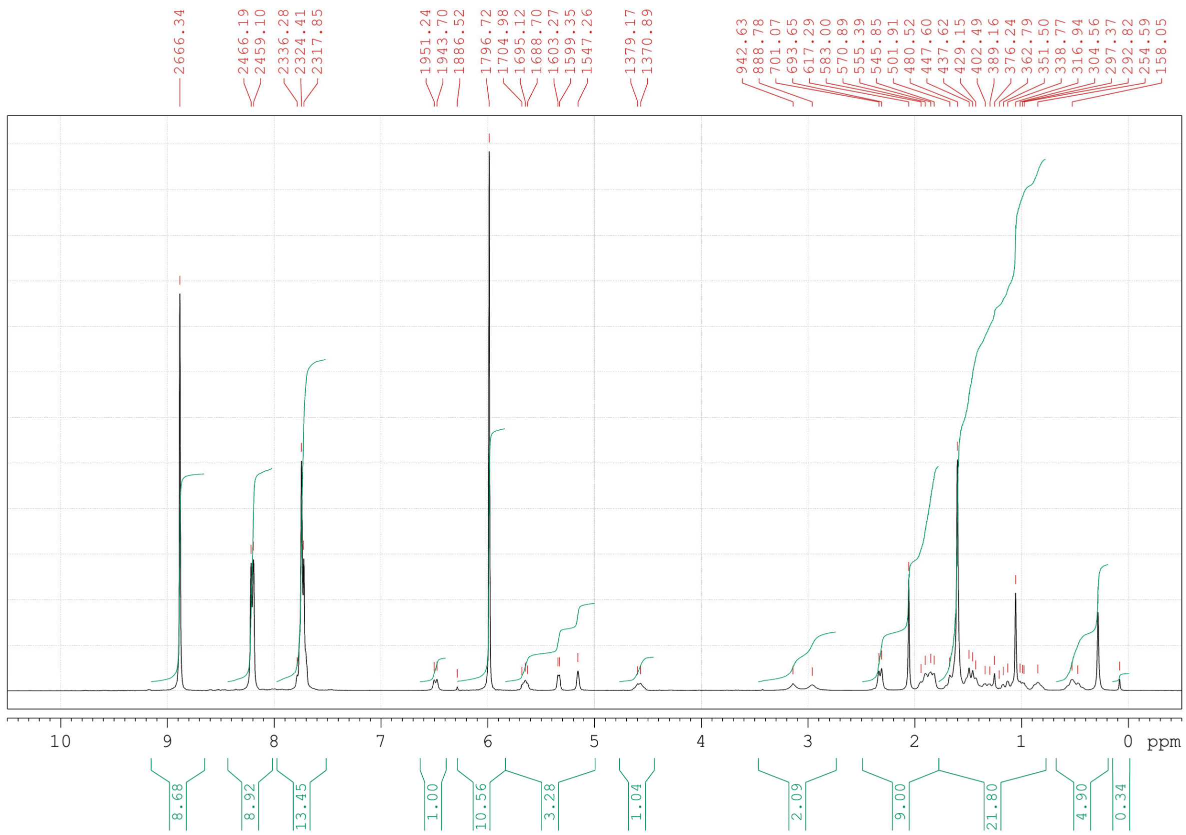

Figure S22. ^1H NMR spectrum

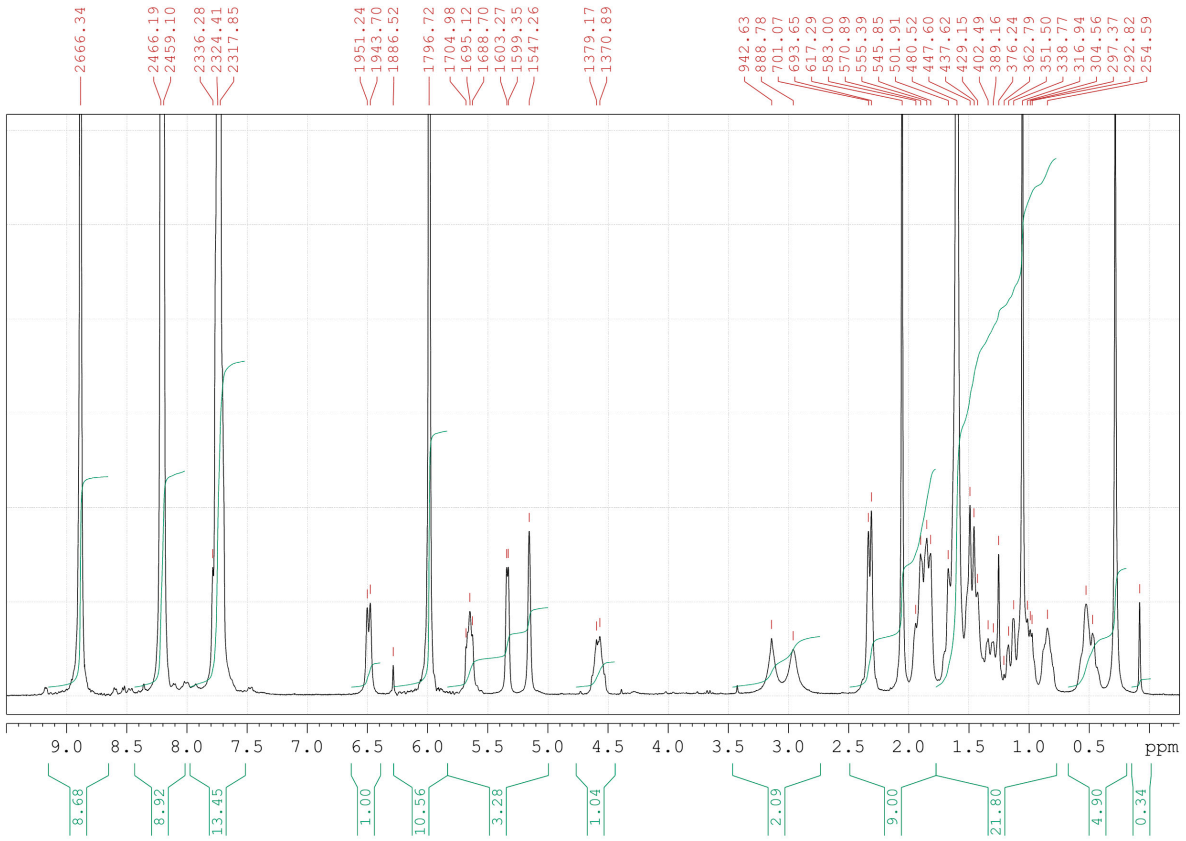

Figure S23. ^1H NMR spectrum

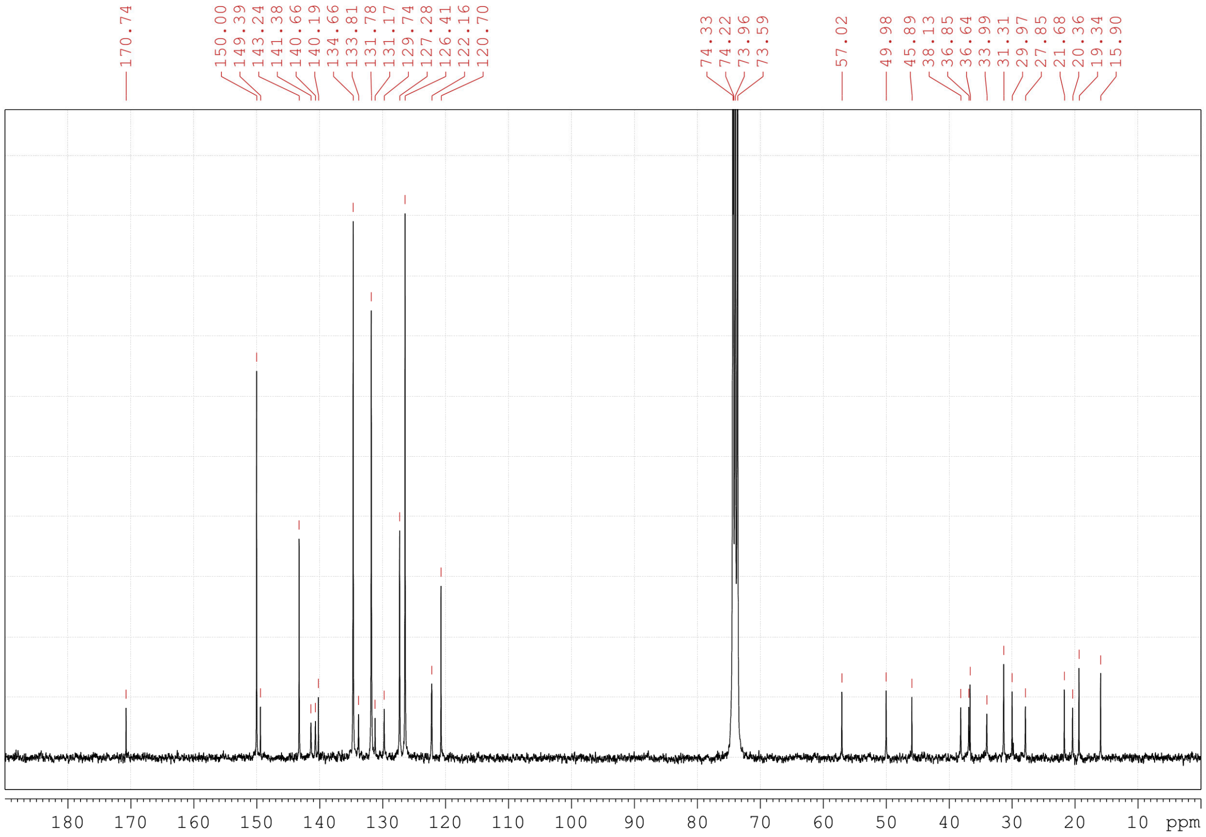

Figure S24. $^{13}C\{^{1}H\}$ NMR spectrum

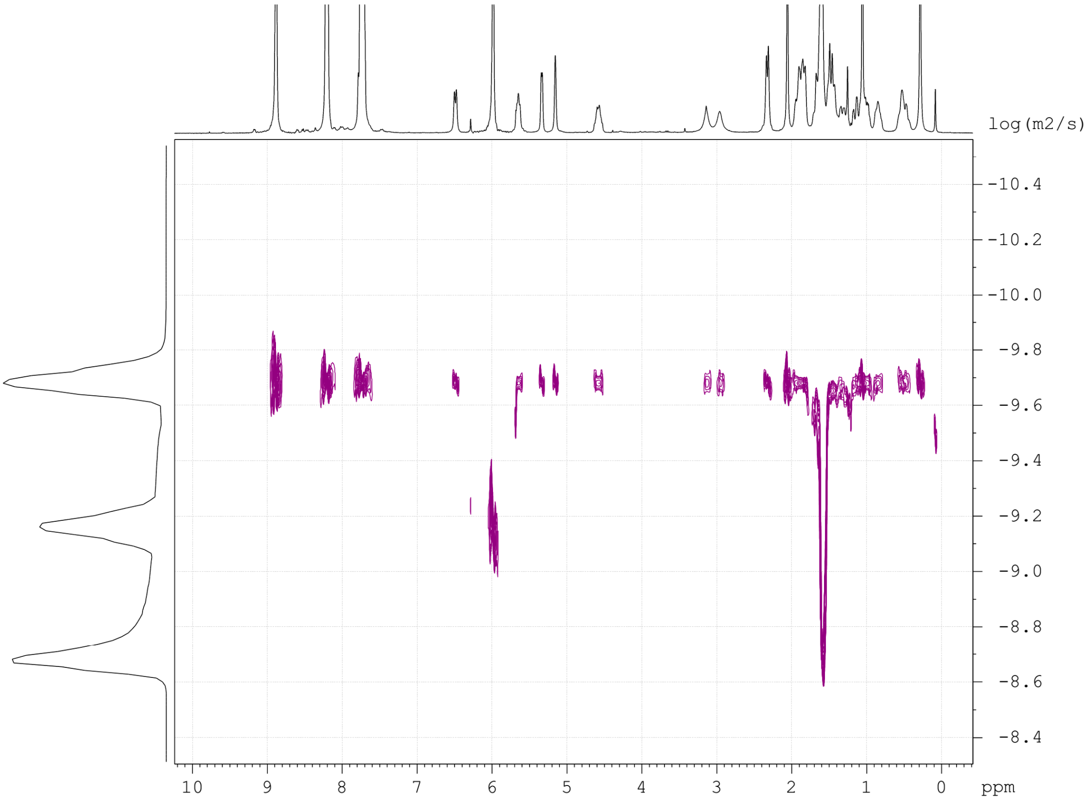

Figure S25. 2D ^{1}H DOSY NMR spectrum

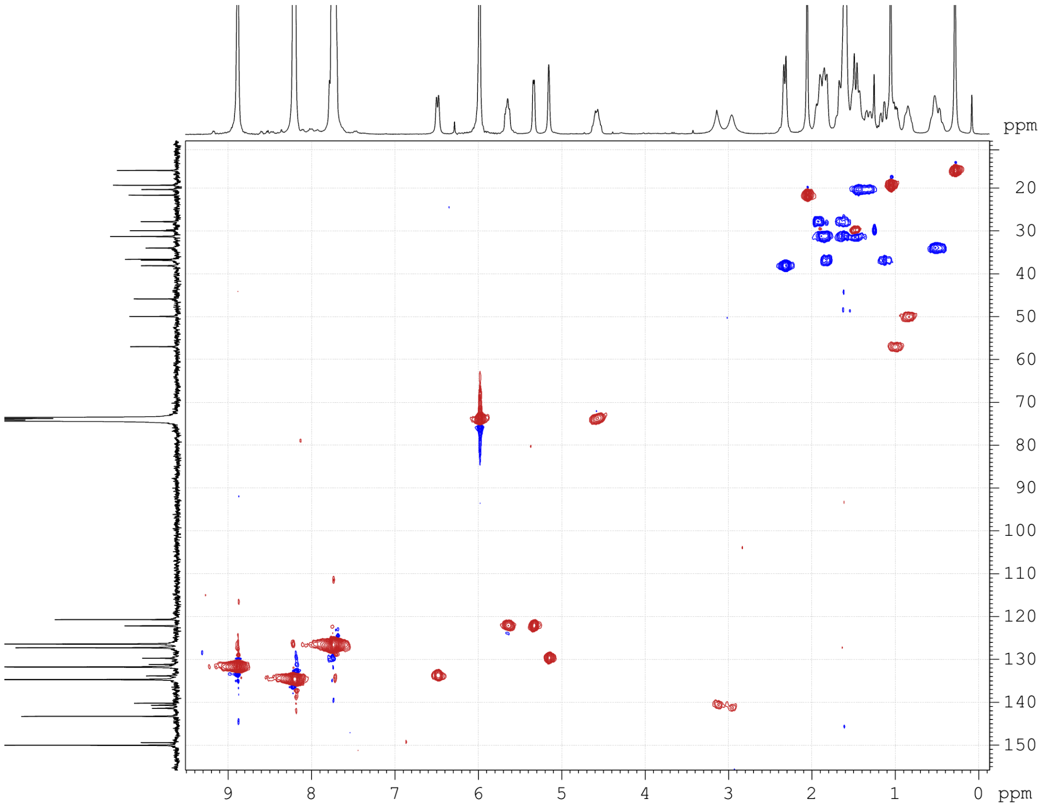

Figure S26. 2D ^{1}H–^{13}C edited-HSQC spectrum

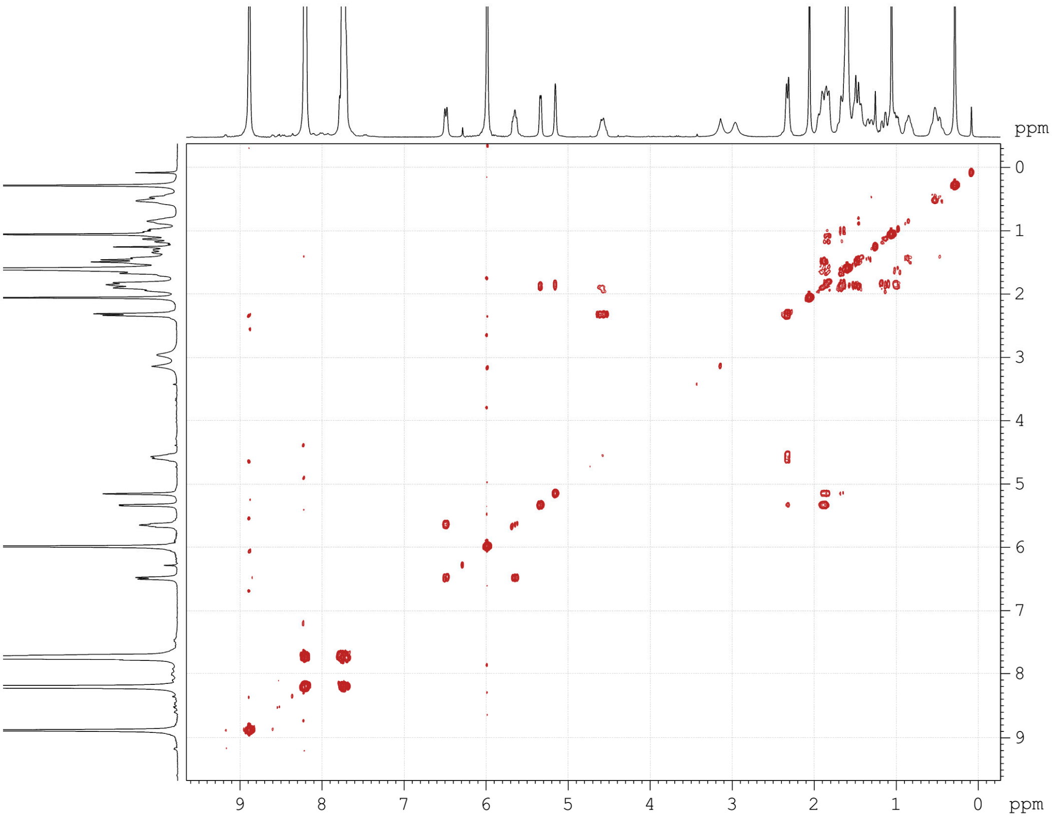

Figure S27. 2D ^{1}H–^{1}H COSY spectrum

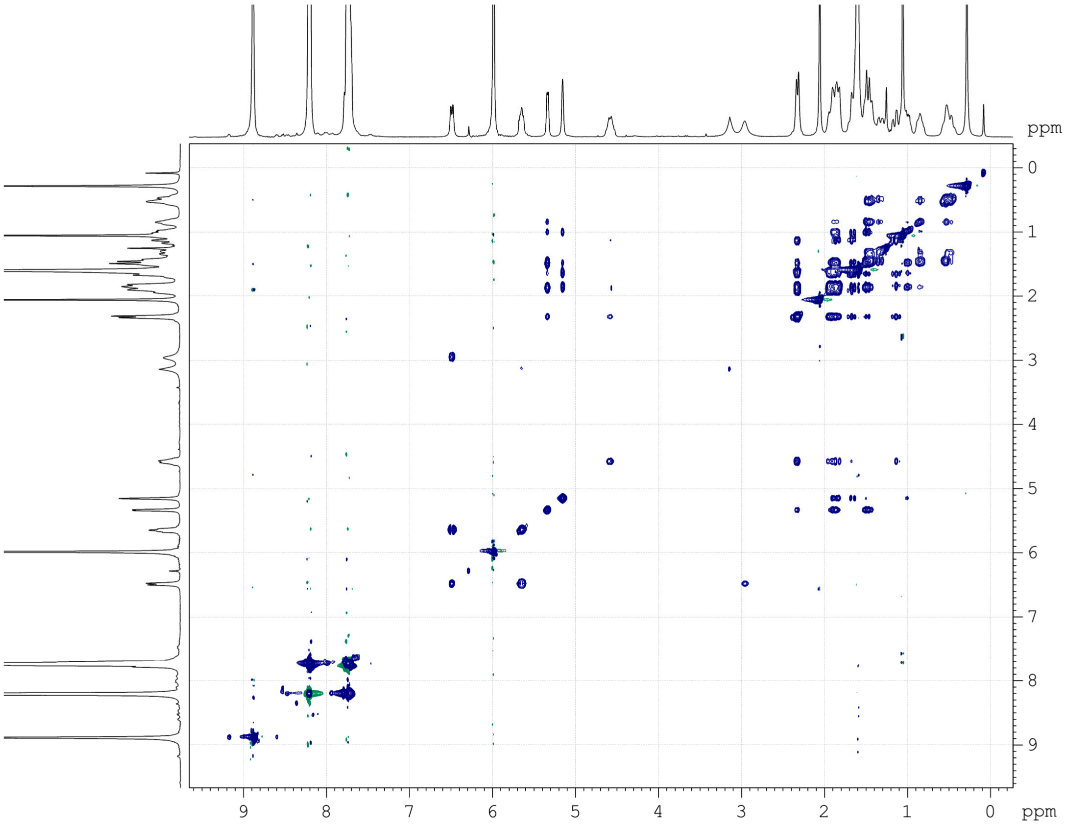

Figure S28. 2D ^{1}H–^{1}H TOCSY spectrum

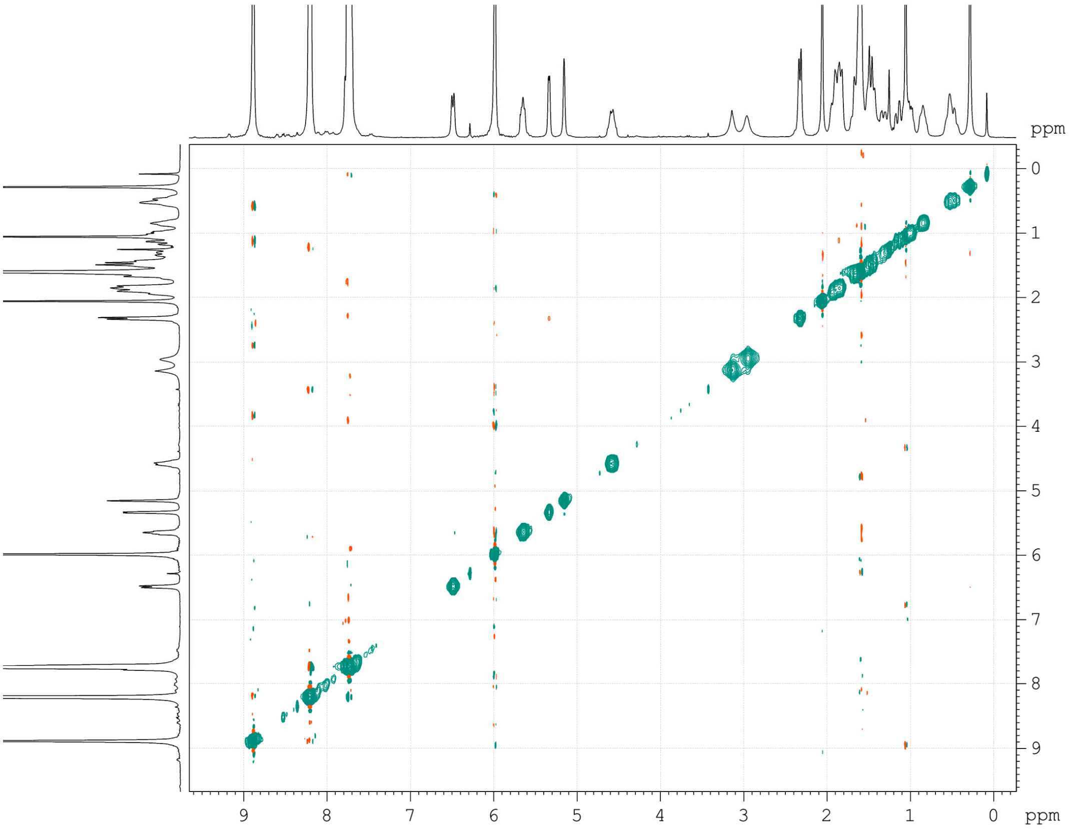

Figure S29. 2D ^{1}H–^{1}H NOESY spectrum

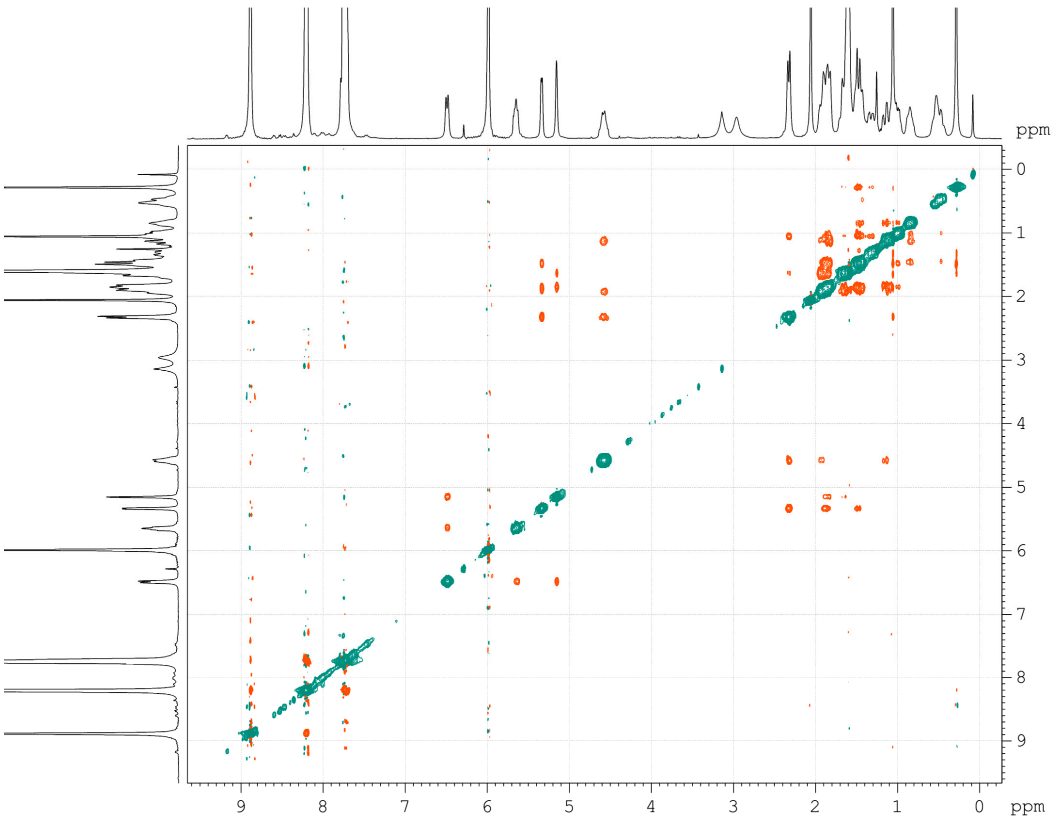

Figure S30. 2D ^{1}H–^{1}H ROESY spectrum

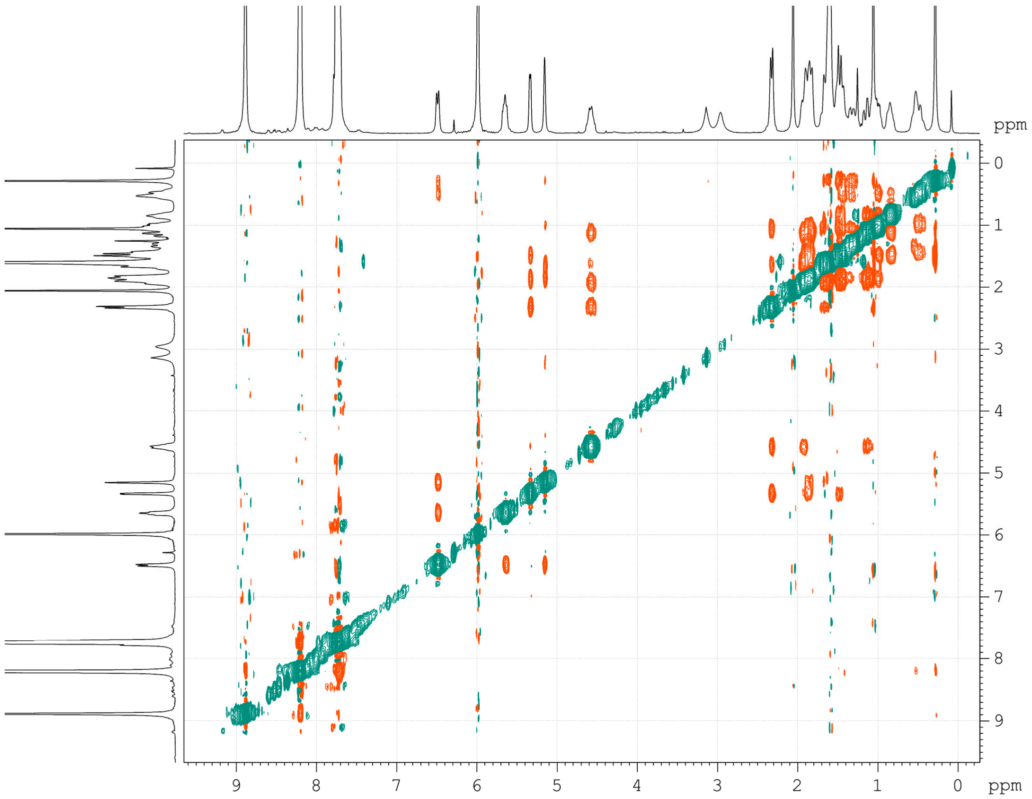

Figure S31. 2D ^{1}H–^{1}H ROESY (x,-x) spectrum

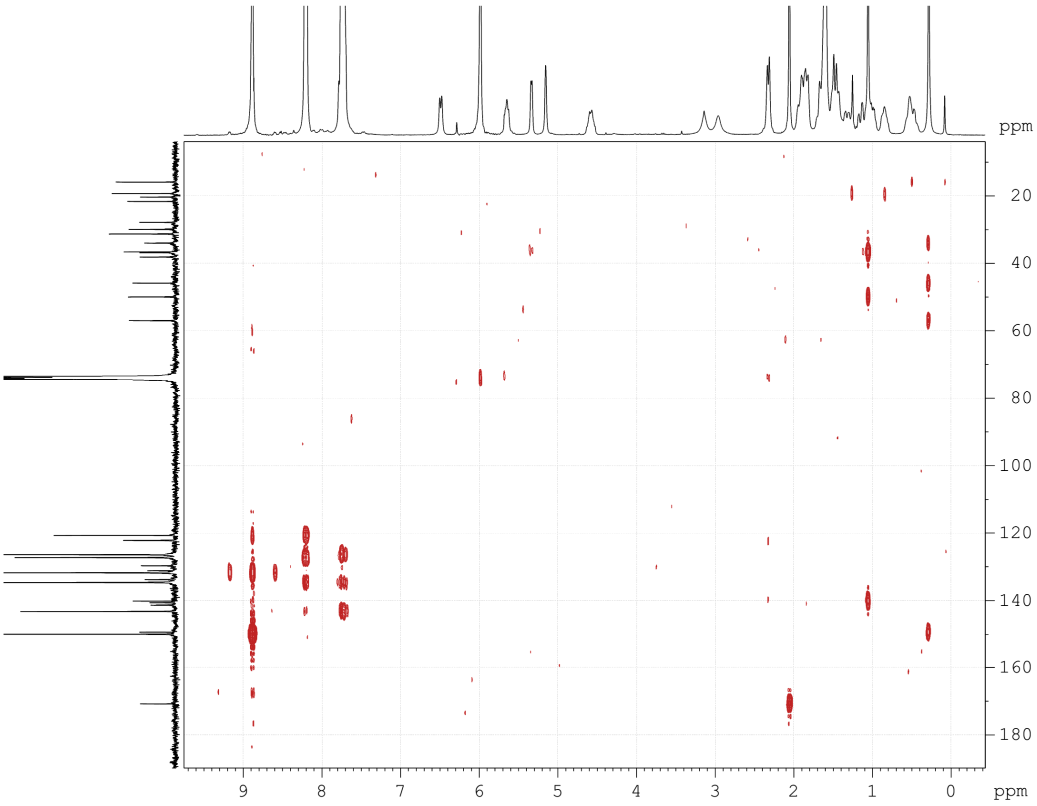

Figure S32. 2D ^{1}H–^{13}C HMBC spectrum

5.4. NMR data for abiraterone in 1,2-TCE-d_2

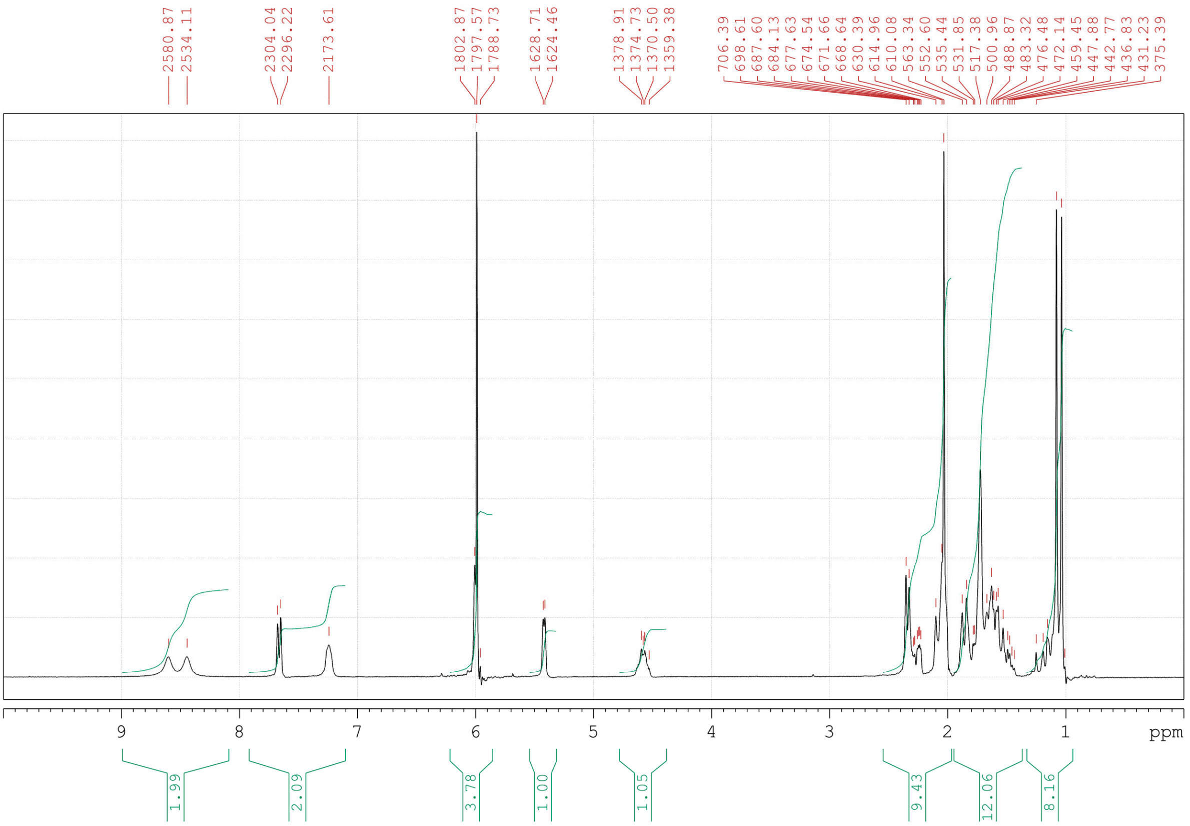

Figure S32. ^{1}H NMR spectrum

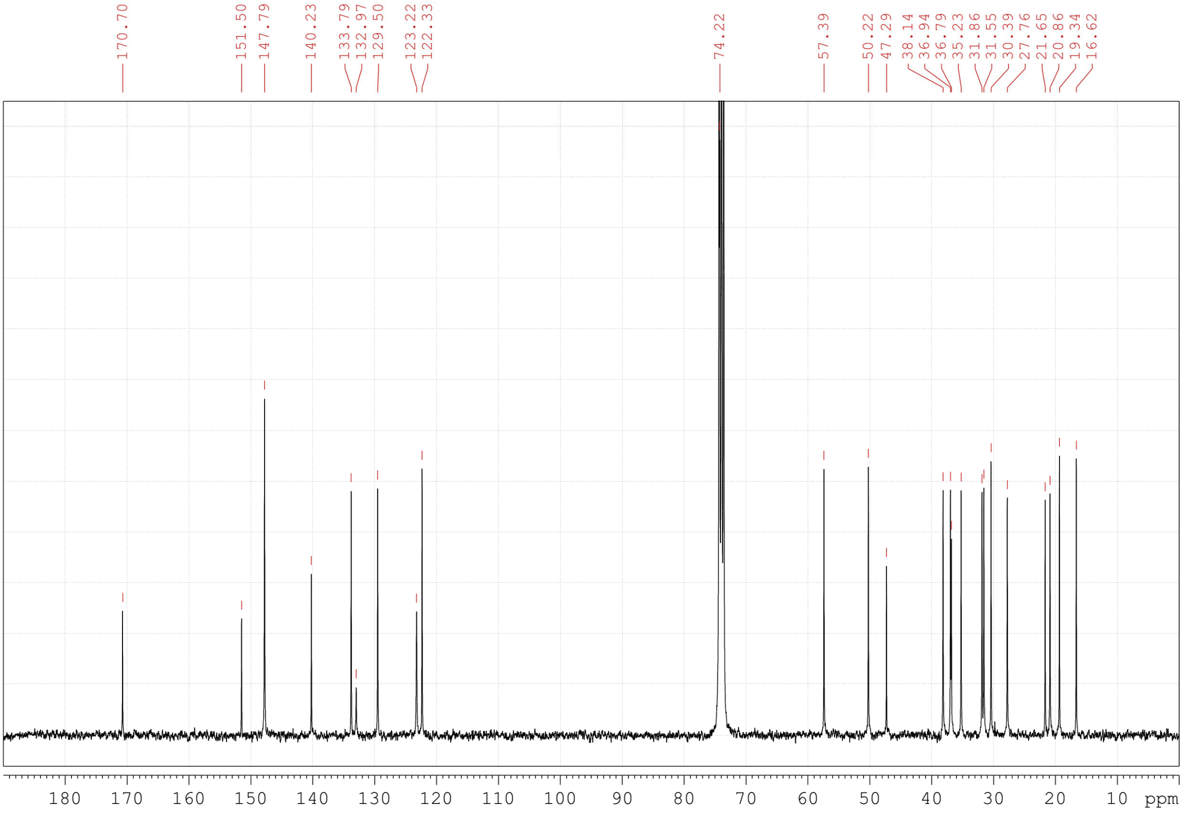

Figure S33. ^{13}C{^{1}H} NMR spectrum

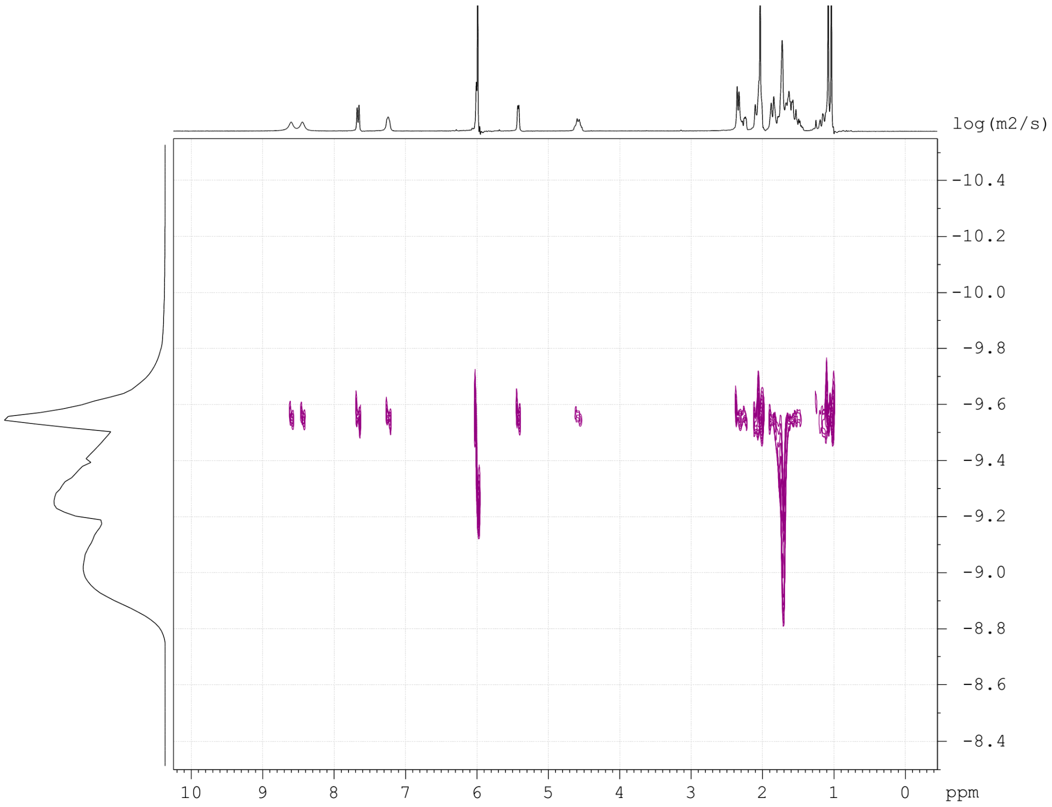

Figure S34. 2D ^{1}H DOSY NMR spectrum

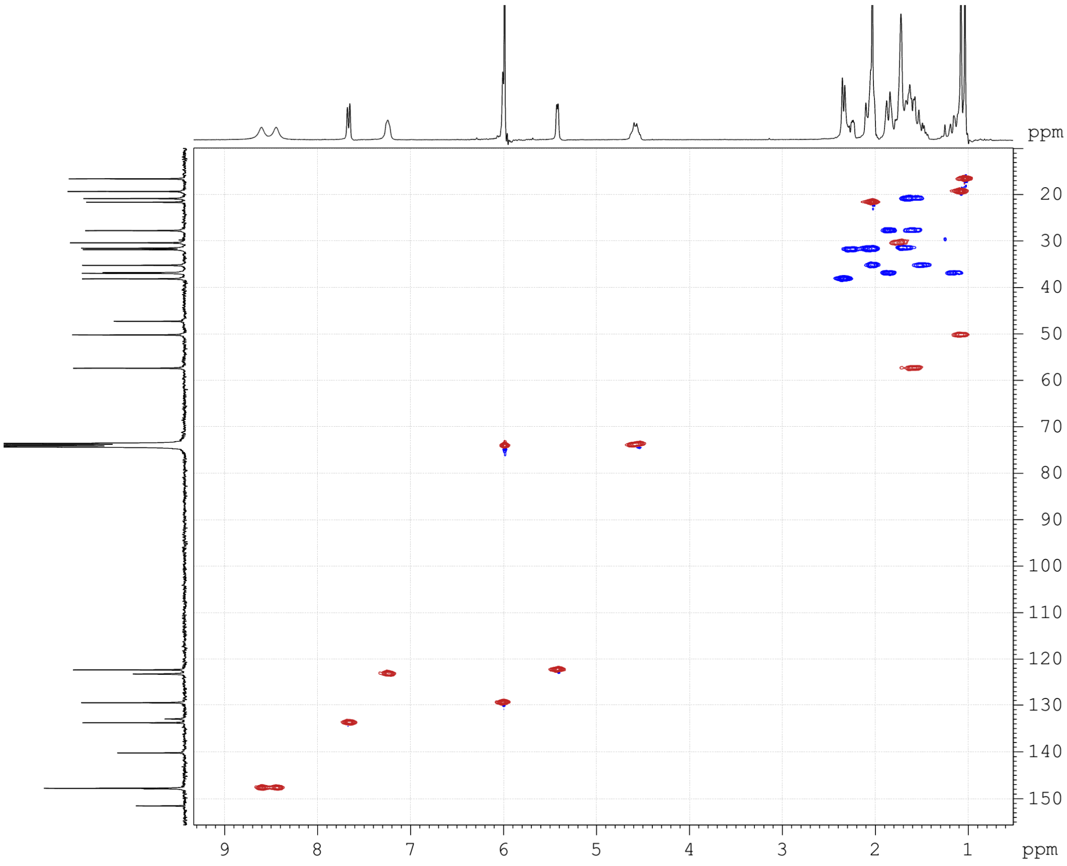

Figure S35. 2D ^{1}H–^{13}C edited-HSQC spectrum

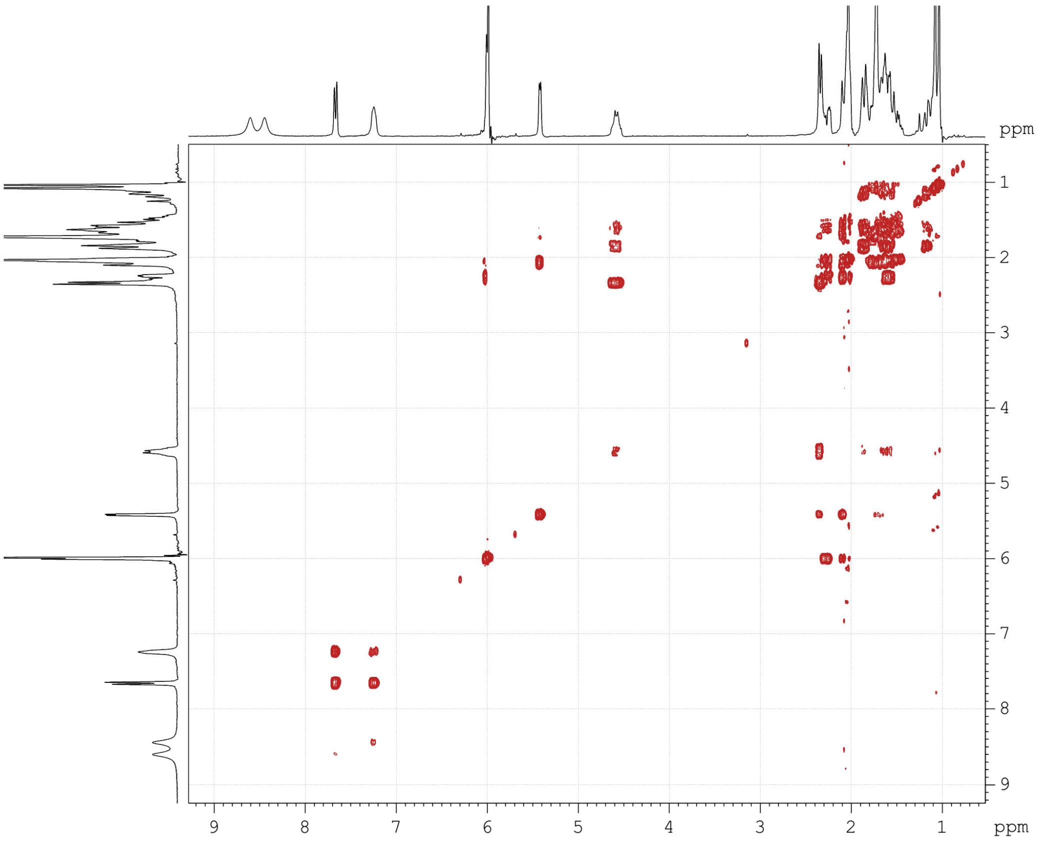

Figure S36. 2D ^{1}H–^{1}H COSY spectrum

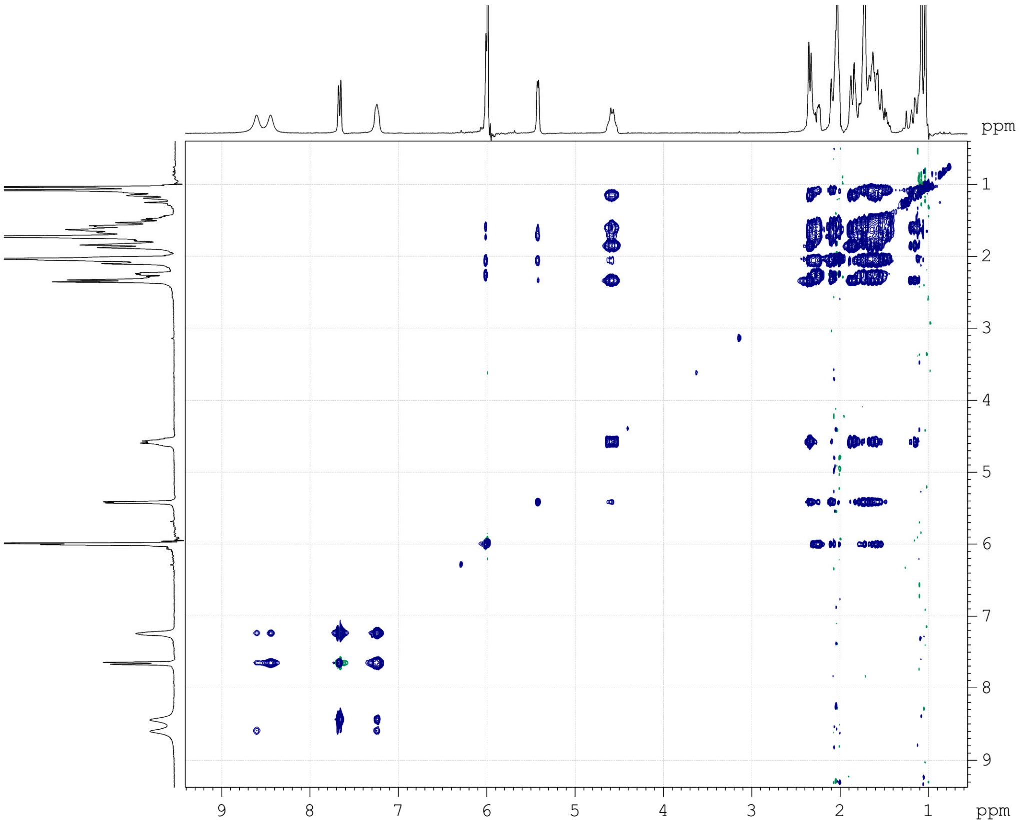

Figure S37. 2D ^{1}H–^{1}H TOCSY spectrum

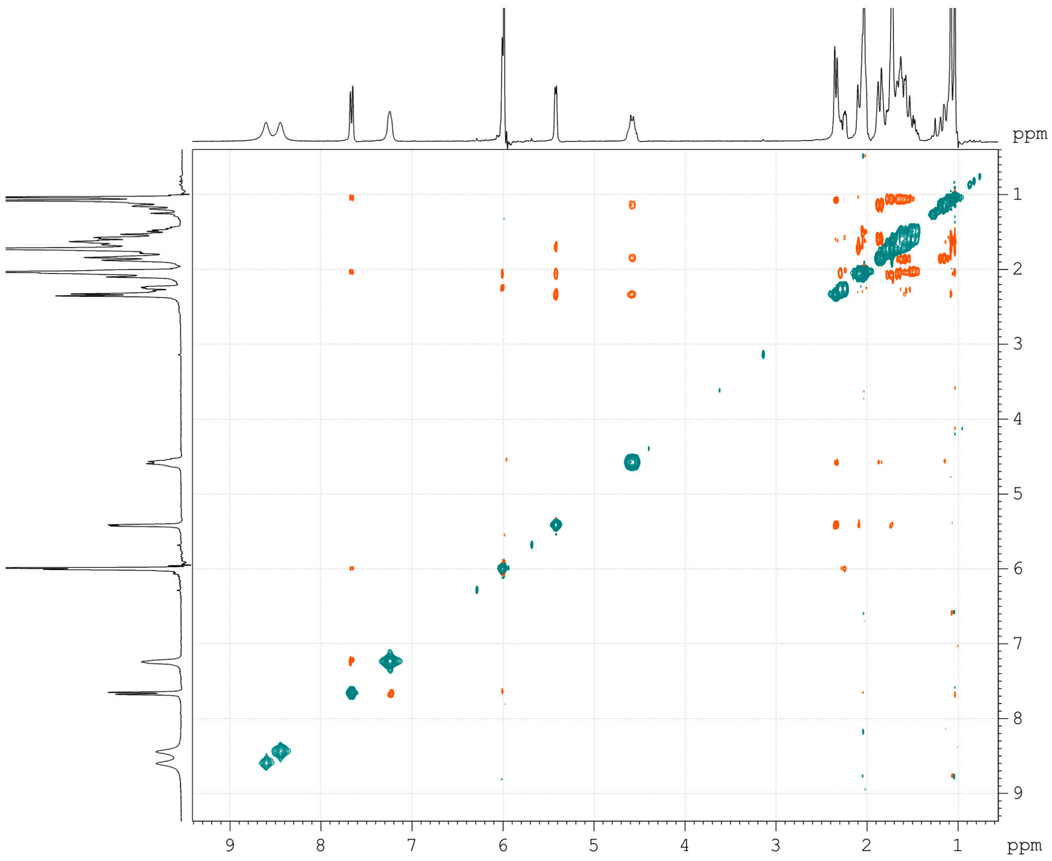

Figure S38. 2D ^1H–^1H NOESY spectrum

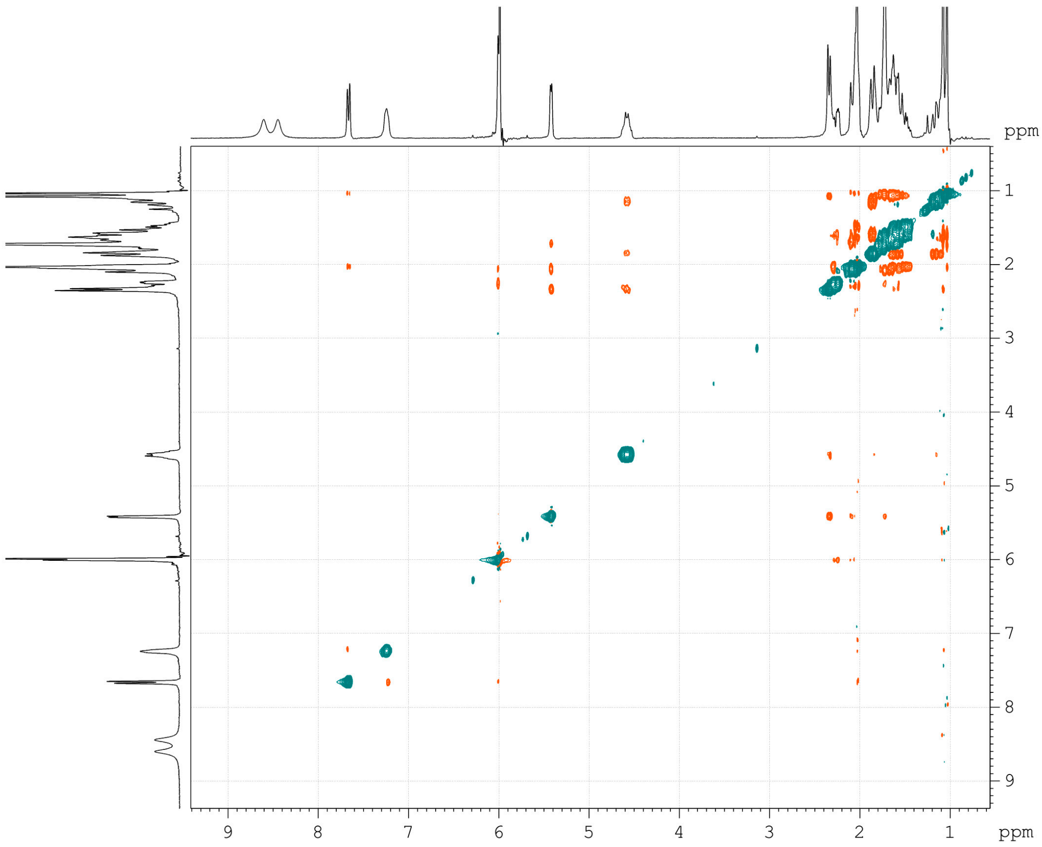

Figure S39. 2D ^1H–^1H ROESY (x,-x) spectrum

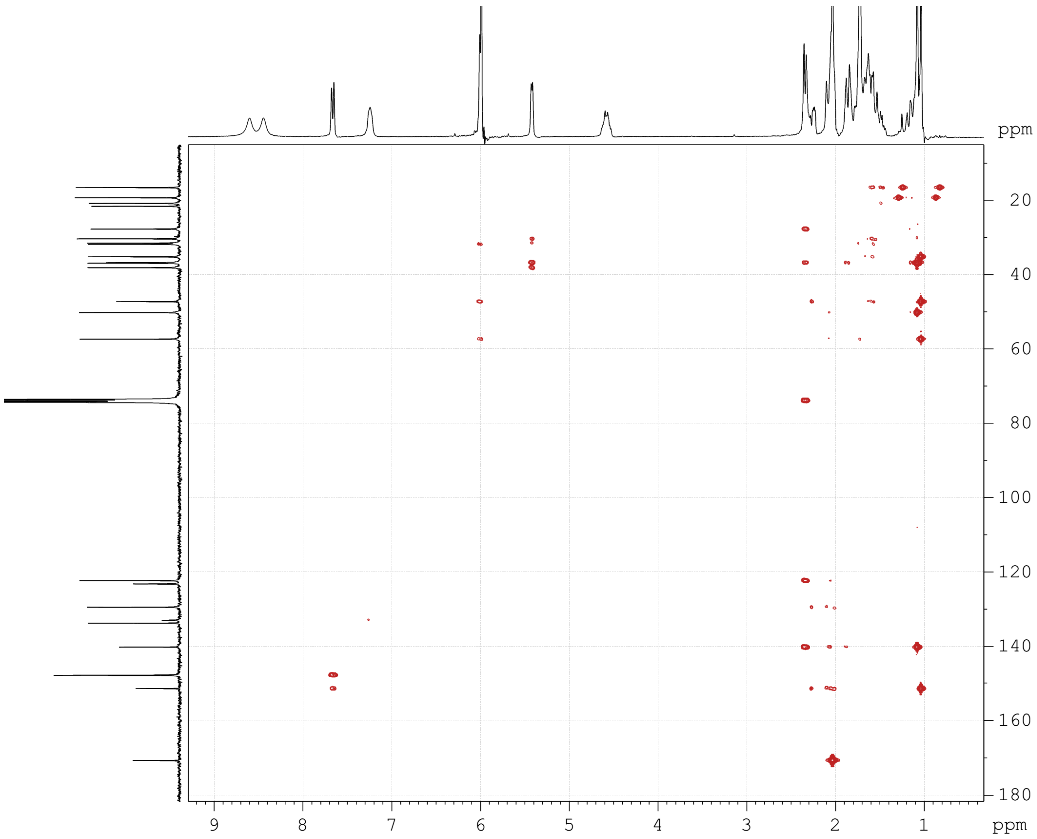

Figure S40. 2D ^{1}H–^{13}C HMBC spectrum

5.5. NMR data for TPPZn in 1,2-TCE-d_2

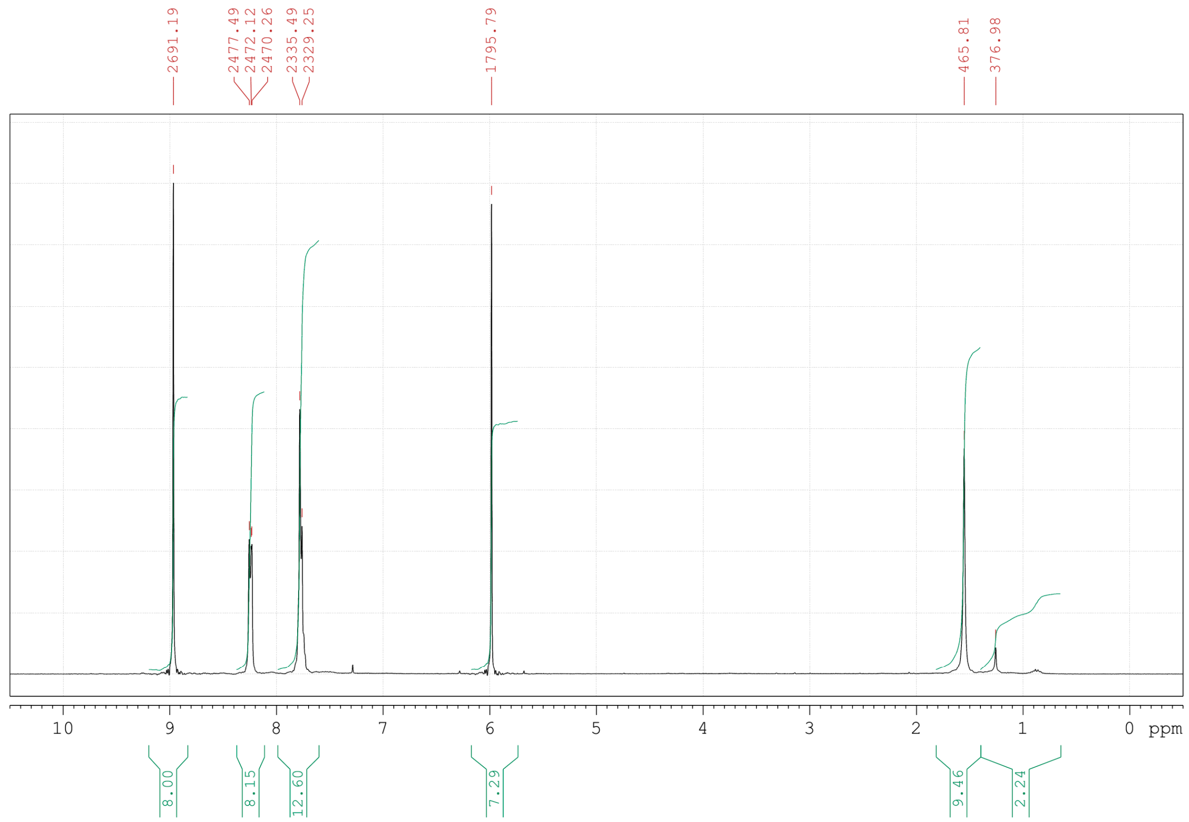

Figure S41. ^{1}H NMR spectrum

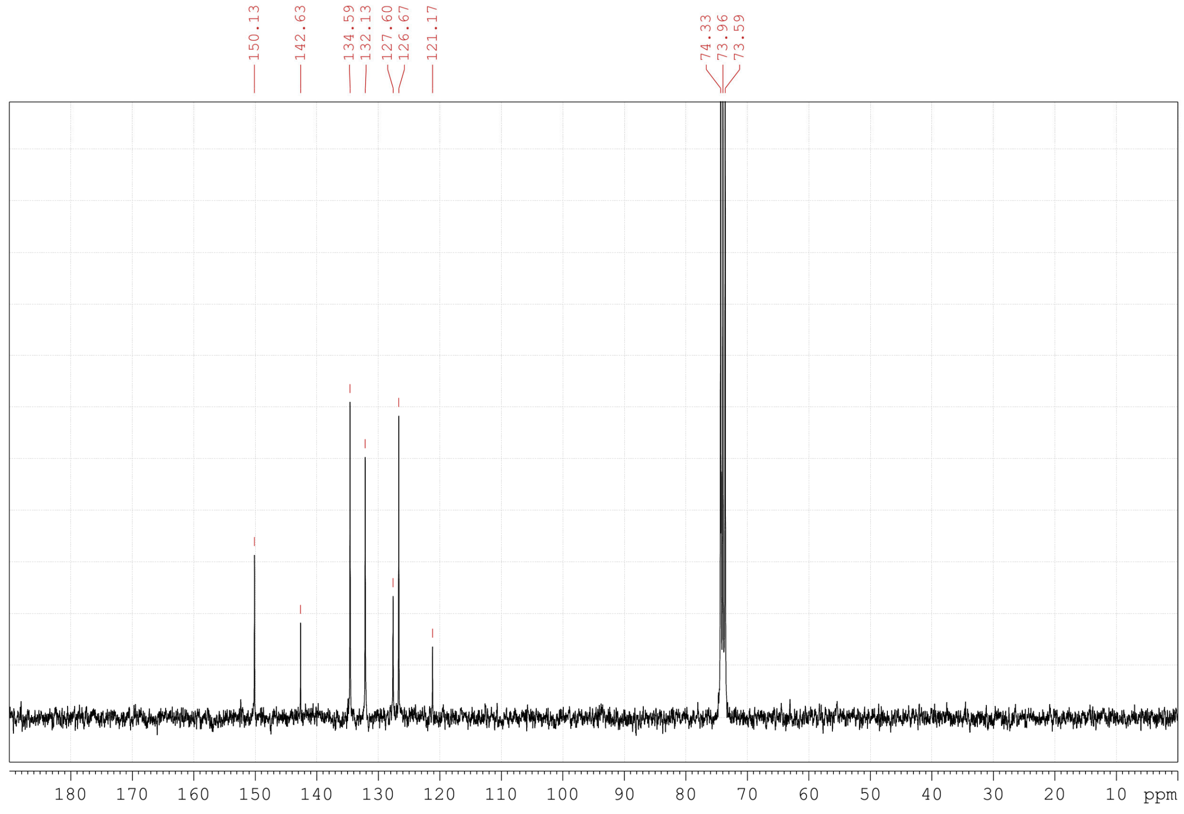

Figure S42. $^{13}C\{^{1}H\}$ NMR spectrum

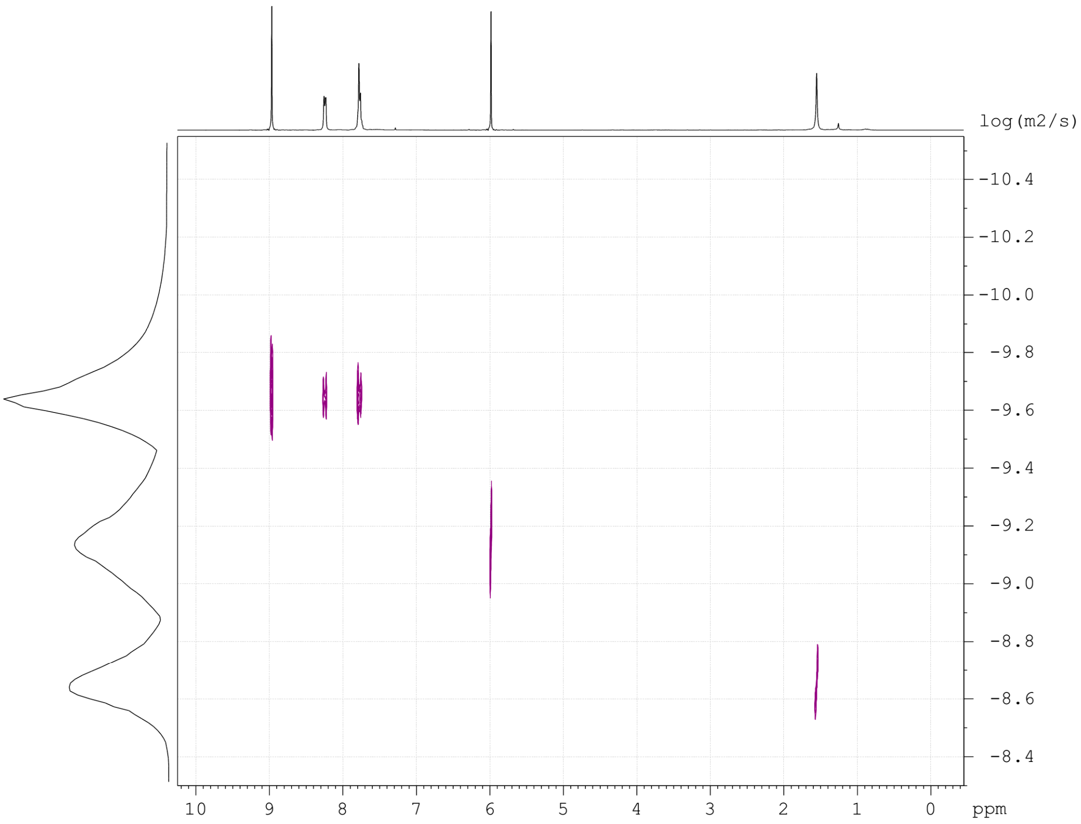

Figure S43. 2D ^{1}H DOSY NMR spectrum

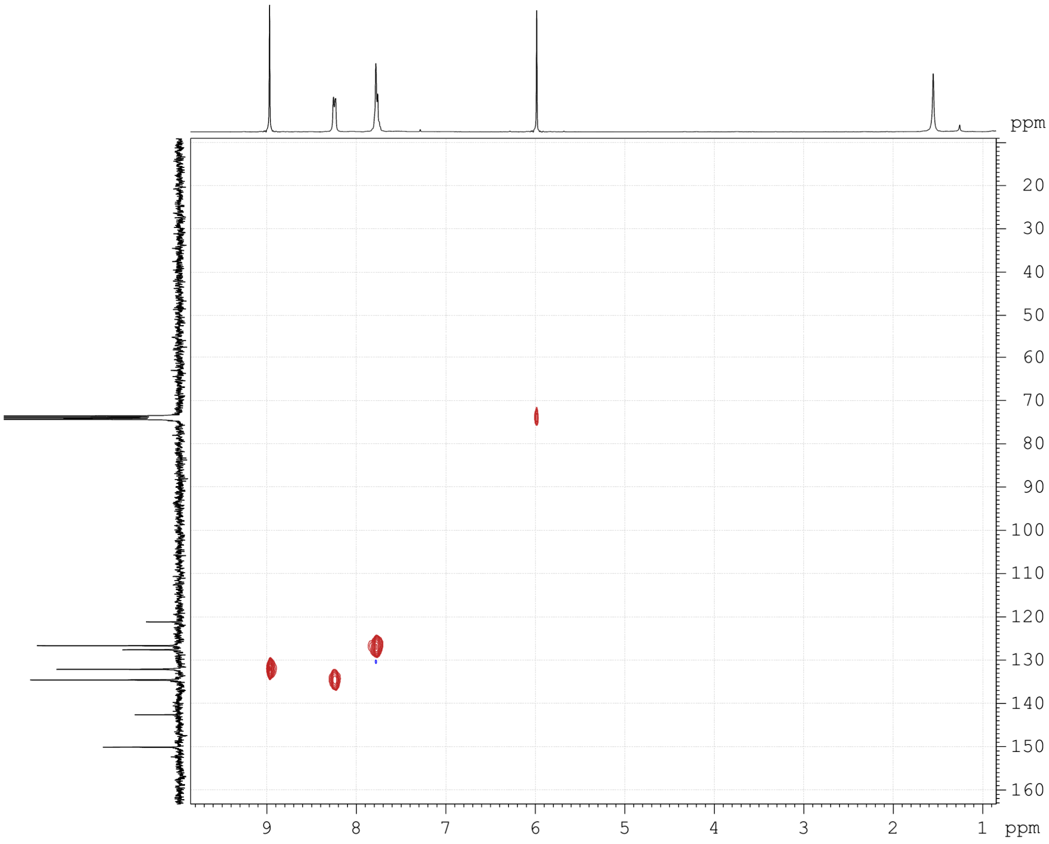

Figure S44. 2D ^{1}H–^{13}C edited-HSQC spectrum

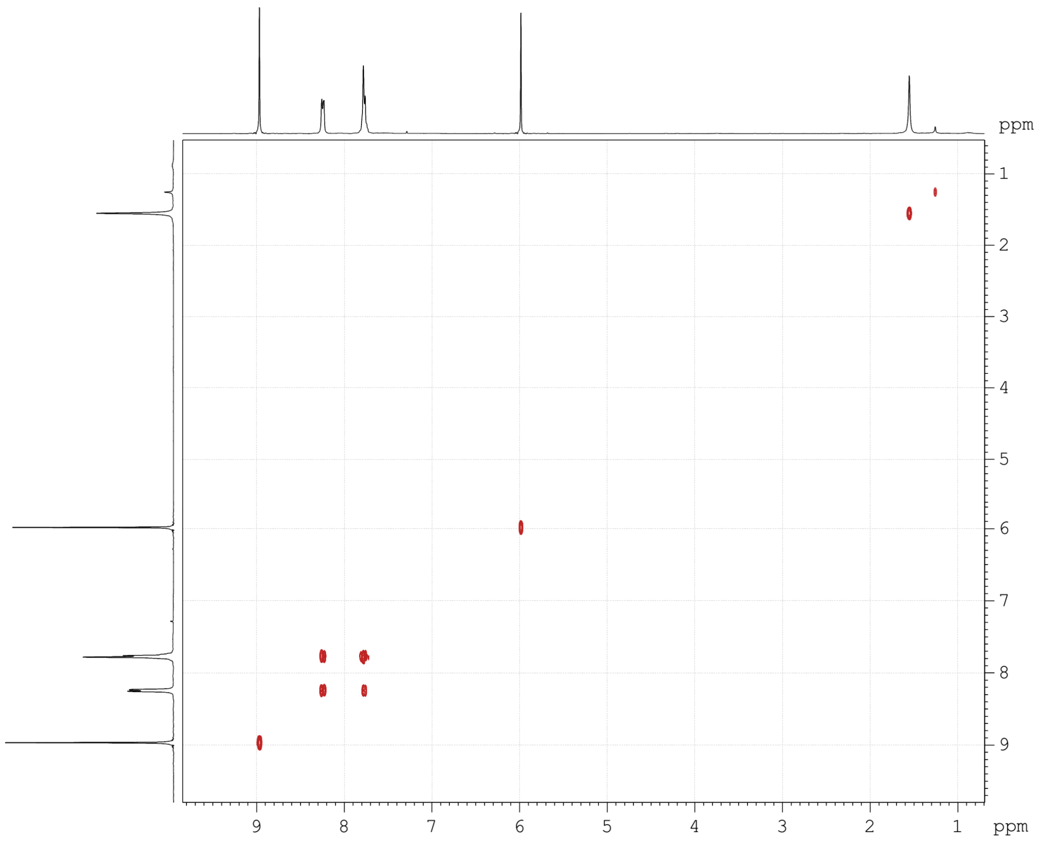

Figure S45. 2D ^{1}H–^{1}H COSY spectrum

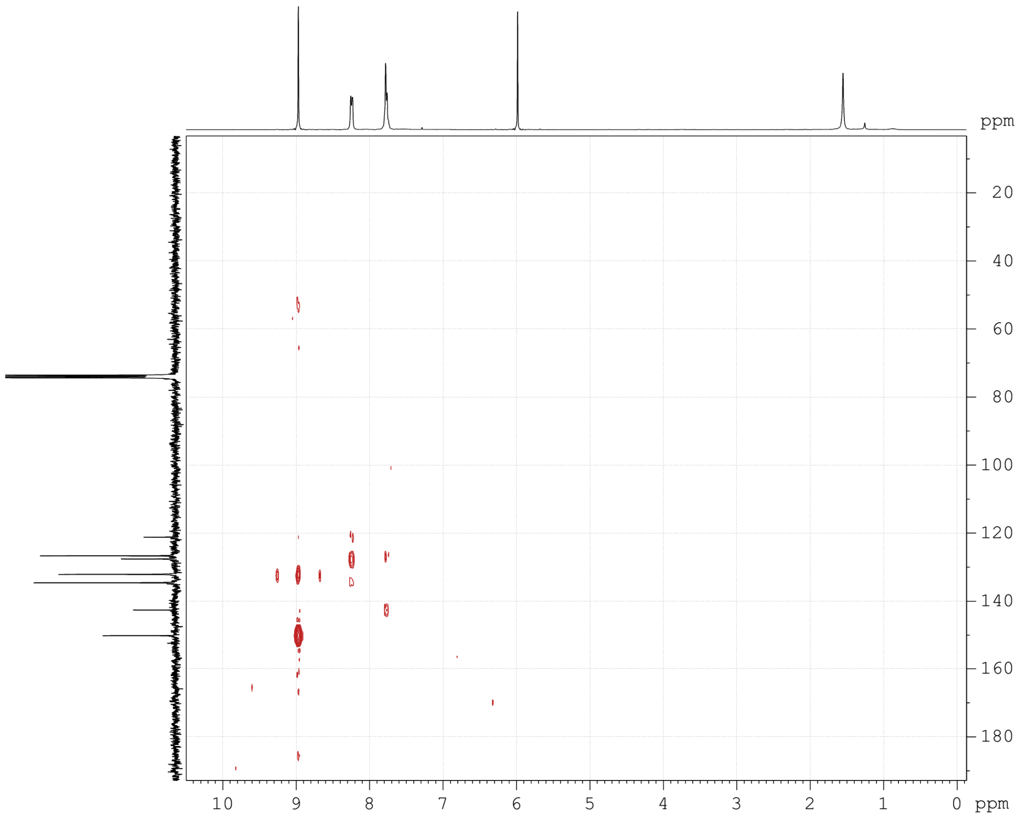

Figure S46. 2D ^{1}H–^{13}C HMBC spectrum

5.6. NMR data for complex in DMSO-d_6

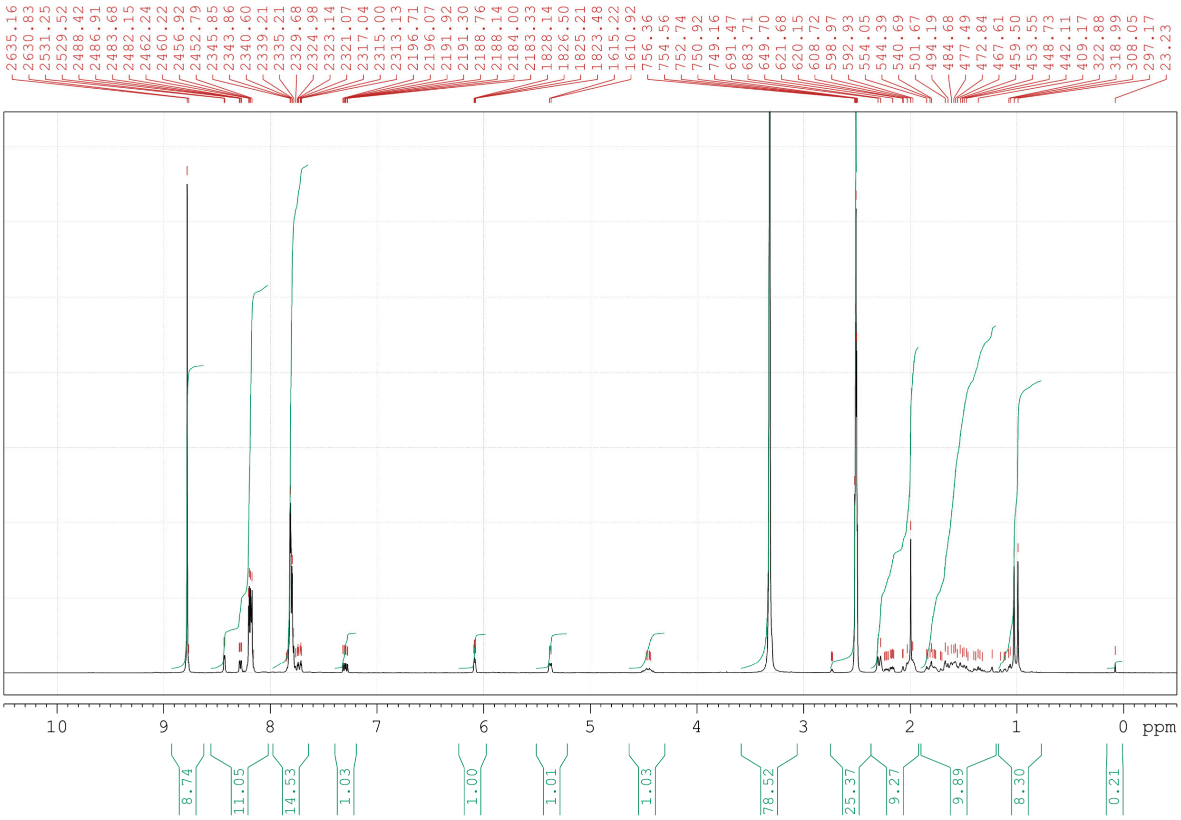

Figure S47. ^{1}H NMR spectrum

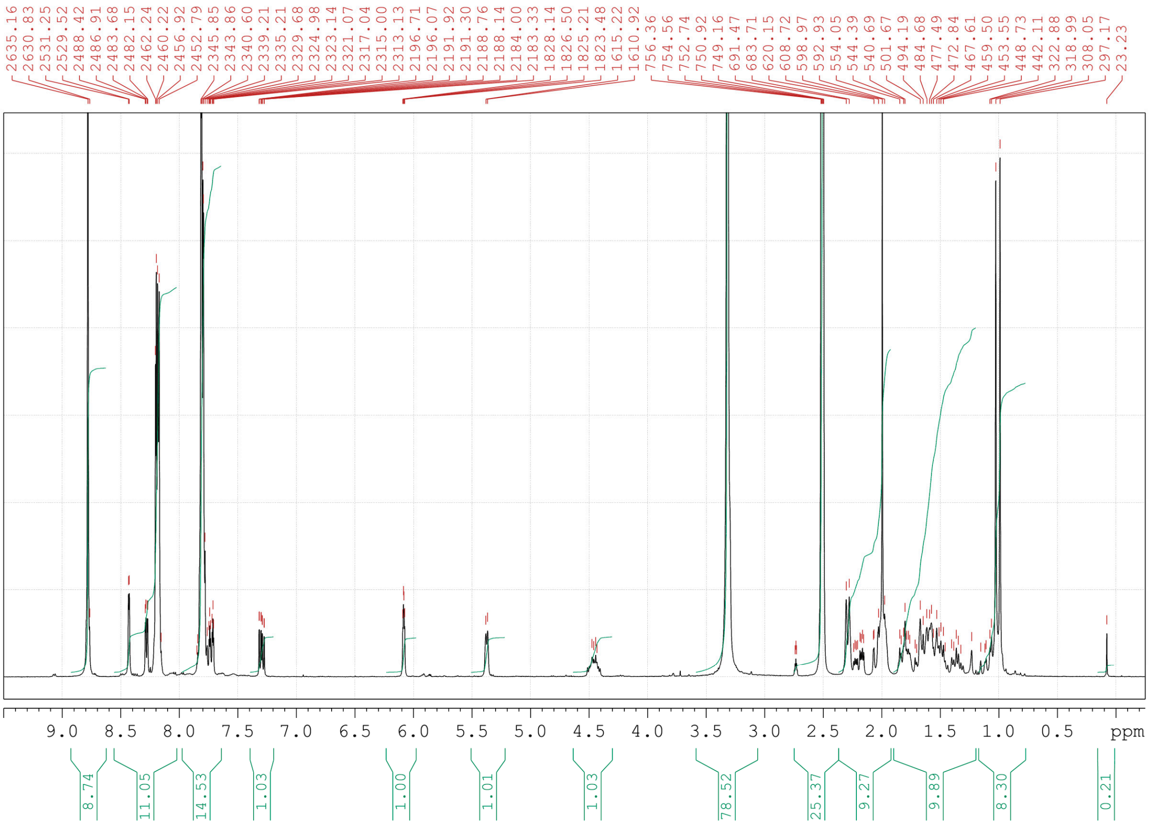

Figure S48. ^{1}H NMR spectrum

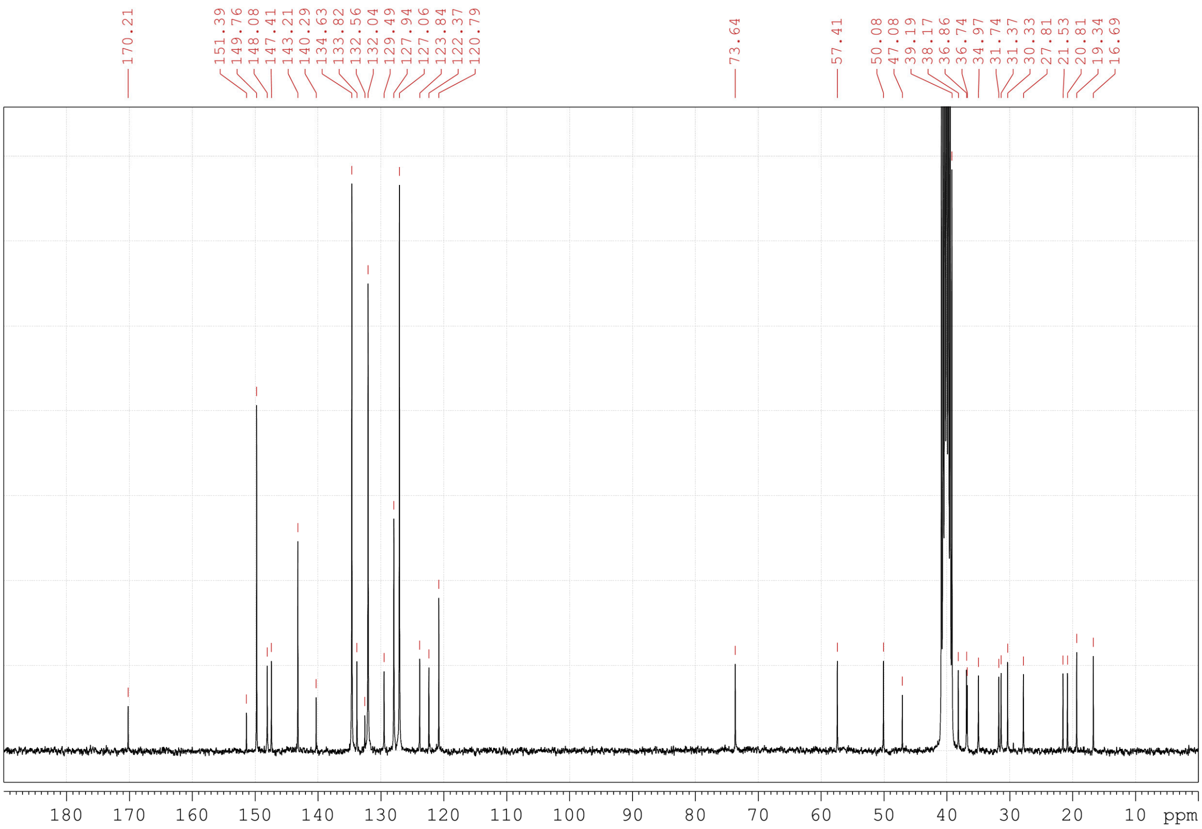

Figure S49. $^{13}C\{^{1}H\}$ NMR spectrum

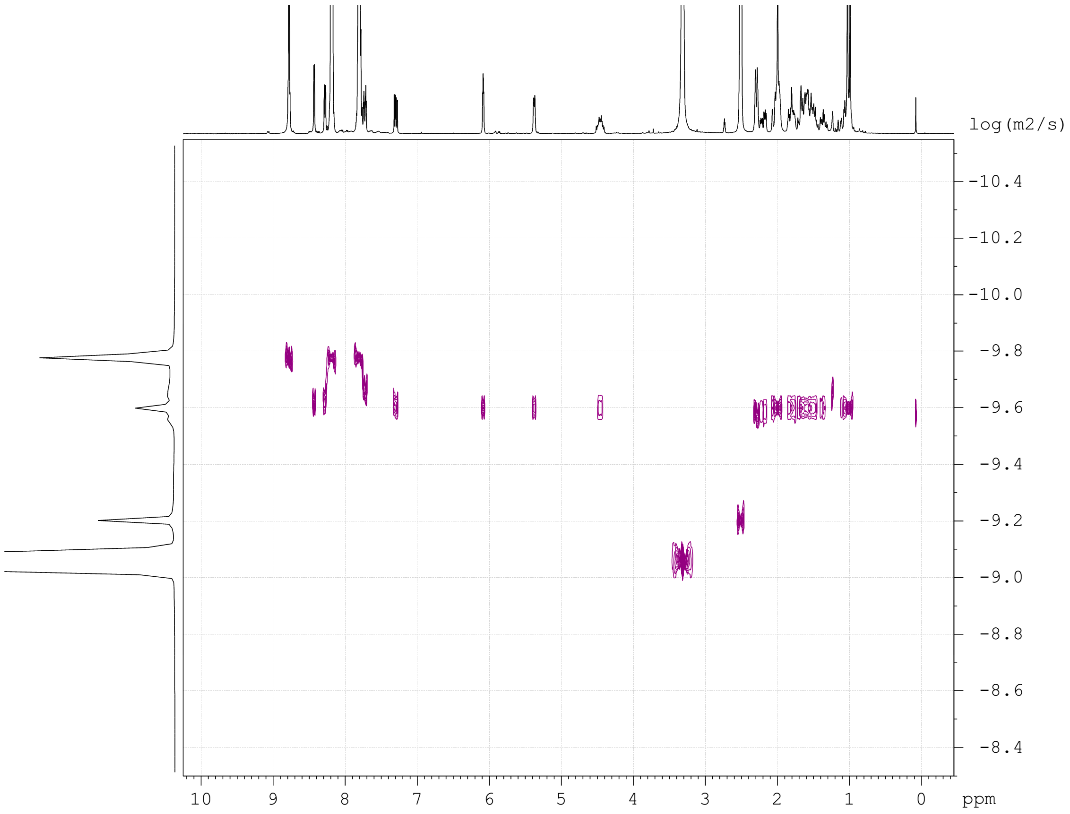

Figure S50. 2D ^{1}H DOSY NMR spectrum

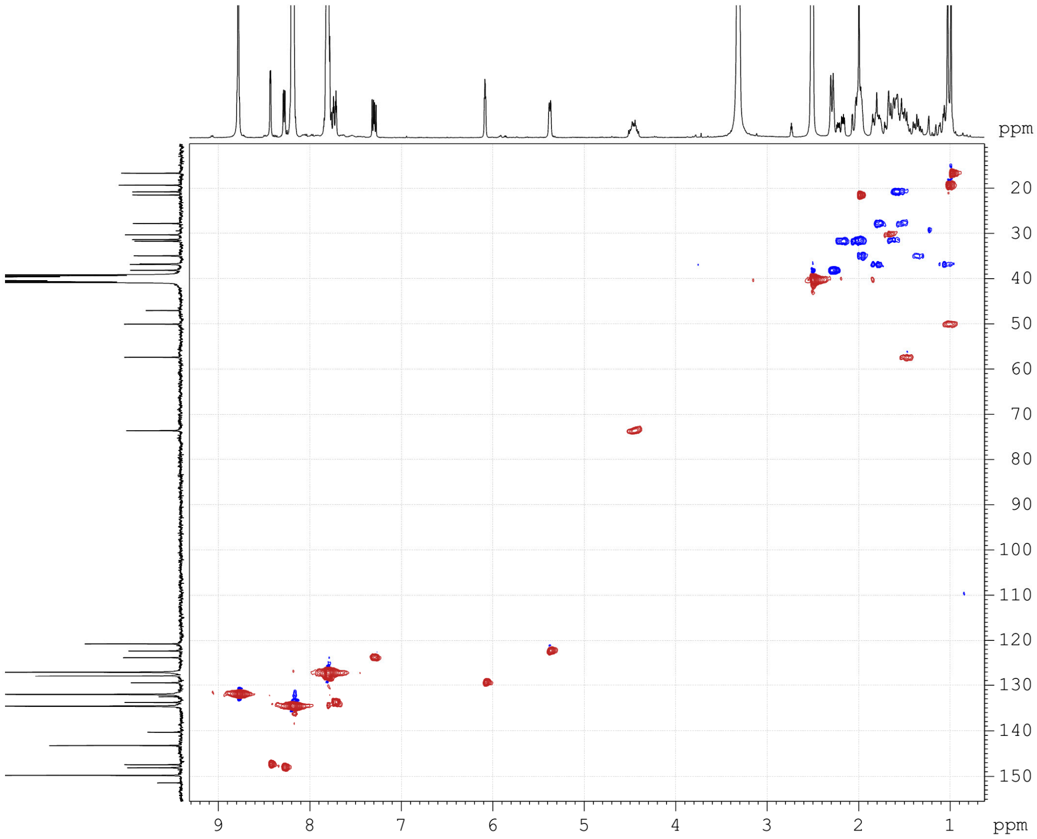

Figure S51. 2D ^{1}H–^{13}C edited-HSQC spectrum

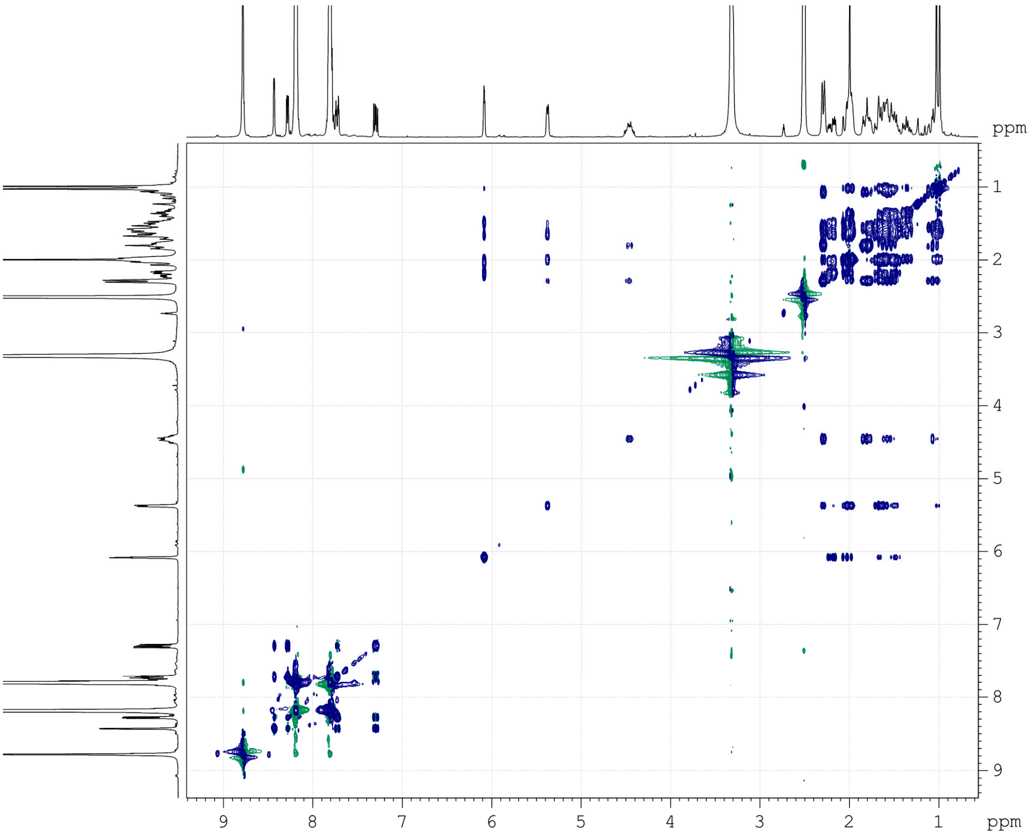

Figure S52. 2D ^{1}H–^{1}H TOCSY spectrum

6. References

S1. Bruker TOPAS 5 User Manual, Bruker AXS GmbH, Karlsruhe, Germany, 2014.

S2. M. Järvinen, J. Appl. Crystallogr. 1993, 26, 525–531.
